# Multimodal X-ray imaging reveals hierarchical fibre mechanics

**DOI:** 10.1038/s41586-026-11148-5

**Published:** 2026-11-04

**Authors:** Elis Newham, Alissa L. Parmenter, Jishizhan Chen, Catherine M. Disney, Tim Snow, Joseph Brunet, Valentin Vinci, Jakub Drnec, Michael J. Sherratt, Judith A. Hoyland, Alexandre Bellier, Brian K. Bay, Nick Terrill, Peter D. Lee, Himadri S. Gupta

**Affiliations:** 1 School of Engineering and Materials Science, Queen Mary University of London, London, UK; 2 UCL Mechanical Engineering, Roberts Engineering Building, University College London, London, UK; 3 UCL Medical Physics and Bioengineering, University College London, London, UK; 4 Research Complex at Harwell, Rutherford Appleton Laboratory, Didcot, UK; 5 Division of Cell Matrix Biology and Regenerative Medicine, School of Biological Sciences, Faculty of Biology, Medicine and Health, University of Manchester, Manchester, UK; 6 Science Division, Diamond Light Source, Didcot, UK; 7 ESRF—The European Synchrotron, Grenoble, France; 8 Department of Anatomy (LADAF), Grenoble Alpes University, Grenoble, France; 9 School of Mechanical, Industrial, and Manufacturing Engineering, Oregon State University, Corvallis, OR USA

**Keywords:** Tissues, Nanoscale biophysics, Biomedical engineering, Imaging techniques

## Abstract

Fibrous materials, ranging from connective tissues^1–4^ to engineered composites^5–8^, are vital to many biological and man-made systems, optimized to withstand complex in operando or in vivo loading. The load-bearing capacity of intervertebral discs (IVDs) of the spine depends on a hierarchical extracellular matrix, where plywood-like lamellae of collagen fibres in the annulus fibrosus contain nanometre-scale fibrils built from staggered triple-helical monomers^4,9,10^. How intact IVDs couple fibril-scale mechanics to fibre-scale organization under load remains unresolved. Here we introduce TomoSAXS, a full-field 3D small-angle X-ray scattering tomography that maps fibril-to-fibre mechanics across an intact tissue. We show that intrafibrillar molecular pre-strain (D-period stagger) is lamellar textured and tightly correlated with microscale fibre strain. Pre-strain is inversely related to fibril strain and its variability, consistent with load-sharing through molecular unwinding. Radial strain bridges and high-curvature zones at the annulus fibrosus–nucleus pulposus interfaces emerge as critical regulators of local mechanics. These findings reveal concerted fibril–fibre interactions that sustain mechanical equilibrium in the IVD, preserving elasticity and shape. More broadly, TomoSAXS establishes a platform to visualize nanoscale-to-microscale matrix mechanics across biological and synthetic fibrous materials, with applications in ageing and disease, therapeutic evaluation and the design of bio-based and bio-inspired materials.

## Abstract (web summary)

The X-ray visualization platform TomoSAXS, demonstrated here on intervertebral spinal discs, can visualize nanoscale to macroscale mechanics in intact biological organs and fibrous materials.

## Main

Fibrous materials provide critical functions across biological and engineered systems. Biological tissues achieve remarkable properties through hierarchical structuring from the nanoscale or molecular scale to whole-organ scales, offering design principles for advanced synthetic materials^5,6^, such as nanostructured polymer composites^7^. Collagen-rich tissues such as tendon, cartilage and bone underpin vertebrate biomechanics, with function emerging from complex, hierarchically structured assemblies of collagen fibrils (typically type I or type II)^9–11^ and non-collagenous components, including hydrated glycosaminoglycans^12^. Distinct features at each hierarchical level, such as nanoscale pre-strain gradients^1^ and microscale variations in fibre orientation and curvature^4^, contribute to tissue performance. However, the complex architecture across scales presents major challenges in isolating the respective effects of each hierarchical element and understanding how they collectively support tissue function.

A clinically important example is the IVD, a sophisticated composite structure in the spine that must balance flexibility with efficient distribution of load^13^. IVD injury and age-related degeneration disrupts the mechanical environment and is a key driver of back pain^14^, the leading global cause of years lived with disability and a major economic burden^15^. Although emerging therapies such as synthetic disc replacements hold promise^16^, their development is hindered by an incomplete understanding of IVD structure–function relationships across length scales.

Our aim is to develop a technique to measure full-field molecular-to-macroscale biophysics of fibrous tissues and uncover new insights into IVD biomechanics. Here we introduce TomoSAXS, a correlative, multiscale X-ray imaging approach that integrates phase-contrast computed tomography (pCT)^17^ at the microscale with small-angle X-ray scattering (SAXS)^18^ at the nanoscale. We applied this technique to intact rat IVDs under physiologically relevant loading to investigate structure–function relationships across multiple length scales. We then extended the technique to intact human IVDs in static conditions.

**Fig. 1:**
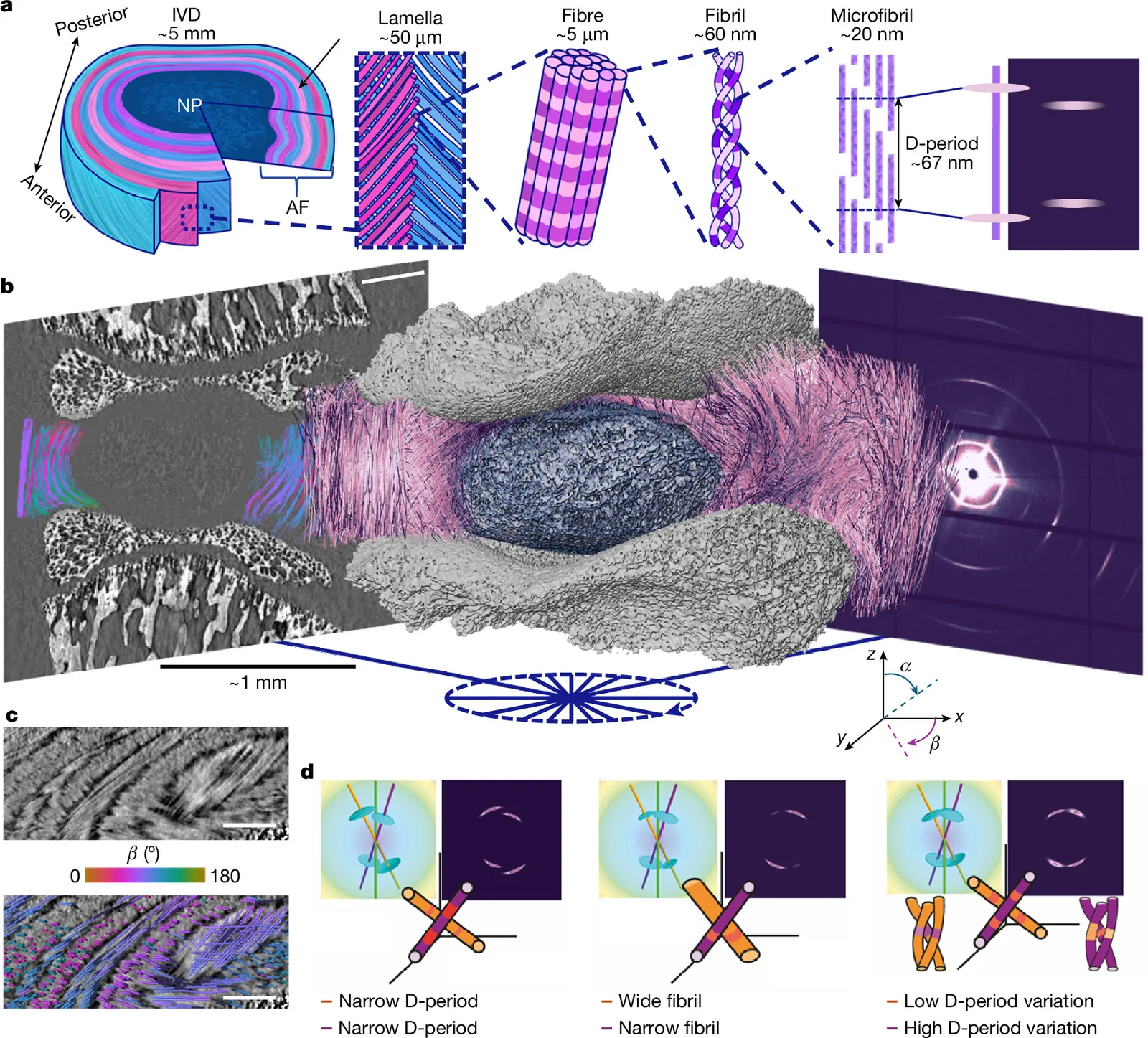
Hierarchical structure of the IVD and correlative pCT and SAXS measurements. **a**, Illustration of the hierarchical structure of collagen in the IVD (from left to right): macroscale structure of the annulus fibrosus (AF) and nucleus pulposus (NP); lamellar plywood-like structure of collagen fibres in the AF; a collagen fibre consisting of multiple fibrils; a collagen fibril formed of helically wound microfibrils; a microfibril of staggered tropocollagen molecules with the D-period labelled; and a schematic of the X-ray scatter produced by collagen D-period. **b**, Multimodal X-ray imaging of the IVD: a slice through the pCT image with traced fibres overlayed and coloured by lateral angle *β* (left; scale bar, 0.6 mm); 3D rendering of the IVD as captured by pCT, with AF fibres rendered in pink, NP rendered in blue and the vertebral endplates rendered in grey (middle); and the resultant SAXS intensity pattern from an X-ray beam path through the IVD (right). **c**, Fibre tracing of individual collagen fibres: a greyscale pCT slice showing a clear fibrous microstructure (top); and the traced fibres overlayed on the pCT image coloured by *β* angle (bottom). Scale bars, 300 µm. **d**, Schematic examples of nanoscale fibril properties reflected in SAXS patterns: D-period, related to peak position along the radial axis (left); fibril diameter, inversely related to the peak width along the circumferential axis (middle); and D-period variation, inversely related to the peak width along the radial axis (right).

The IVD consists of a central nucleus pulposus—a hydrated proteoglycan-rich matrix that redistributes mechanical loads—surrounded by the annulus fibrosus, a fibrous outer ring that provides structural support and stability^19^ (Fig. 1a, first panel). Within the annulus fibrosus, concentric lamellae tens of microns thick are composed of collagen fibres (1–20 μm in diameter) with alternating orientations between adjacent lamellae (Fig. 1a, second panel), forming an architecture believed to convert nucleus pulposus-derived hydrostatic pressure into circumferential tensile stress^13,19^. An inter-lamellar network, composed of a non-fibrillar matrix, elastic fibres and trans-lamellar cross-bridges, further stabilizes this structure by anchoring adjacent lamellae^20^.

Imaging of this fibrous microstructure across the whole organ is challenging due to the similar density of fibres and the surrounding matrix, and fibre analysis of the IVD has generally been limited to small (less than 0.5 mm) regions of interest^4,21^. The phase information revealed by pCT, combined with digital volume correlation (DVC)^4,21–24^, is uniquely suitable to resolve the low-contrast fibre structure (Fig. 1c) and volumetric in situ deformation and strain at the microscale. Recent applications of the DVC have revealed intricate, region-specific strain patterns, with tissue compliance linked to fibre curvature and orientation^4,21^. These complex patterns depend crucially on the intrinsic structural properties of the fibres.

At the nanoscale, collagen fibres are composed of fibrils (approximately 20–100 nm; Fig. 1a, fourth panel), which are in turn formed of microfibrils of staggered tropocollagen molecules with a mean periodicity (D-period) of approximately 65–67 nm (ref. ^9^) (Fig. 1a, fifth panel). This axial staggering gives rise to characteristic SAXS diffraction patterns with Bragg peaks at integer multiples of 1/*D* (Fig. 1a, sixth panel), which act as an internal gauge of fibrillar strain, orientation and diameter (Fig. 1d). In other fibrous tissues—such as bone, tendon and cartilage—two-dimensional (2D) transmission-based SAXS has been used to identify nanoscale deformation mechanisms, including fibrillar pre-strain gradients and hydration-induced alteration in diameter in cartilage^1^, nanoscale response to in situ loading in tendons^25^ and altered mineral-collagen shear-transfer in ageing bone^26^. 2D SAXS analysis of the IVD has demonstrated that energy dissipation occurs through fibril reorientation and stretching^27^, but interpretation has been limited due to a lack of 3D information.

Linking 3D deformation of fibrous structures across scales using SAXS and pCT poses major challenges. Standard SAXS analysis is based on 2D convolutions of combined 3D reciprocal scattering from multiple fibres encountered along individual X-ray beam paths. As fibre scattering is anisotropic, the 2D pattern depends on fibre orientation at each scattering voxel^1^, invalidating traditional scalar tomographic methods for macroscopic specimens with complex fibre texture^28^. To overcome this, tensor tomographic methods have been developed that use multi-axis rotation and spherical basis functions to reconstruct 3D nanostructure in a voxel-wise manner in undeformed structures^29–32^. The number of angular projections required for reconstruction increases scan times and limits the scalability of the technique to large samples, and the need for multi-axis rotation makes in situ experimentation complex. There remains an unmet need for methods to reconstruct both microstructure and nanostructure of individual fibres and track their heterogenous deformation within the broader context of microscale-to-macroscale analysis.

The main experiment presented here uncovers nanoscale-to-macroscale biomechanics of rat IVD during in situ mechanical loading. Here TomoSAXS uses fibre tracing of collagen fibres in the annulus fibrosus from pCT reconstructions and their volumetric registration with tomographic SAXS data to allow deconvolution of the 3D scattering from individual fibres in each SAXS beam path. The IVD is an excellent biological exemplar due to its lamellar fibrous texture allowing deconvolution of contribution from different fibrils under rotation. Instances through the SAXS tomography where fibres are predicted to provide sufficient portions of independent scattering intensity are sampled and fitted with 3D fibre-diffraction models to estimate their individual nanoscale properties (mean and variation in D-period and fibril diameter). Registration between SAXS and pCT volumes allow these to be volumetrically mapped onto pCT reconstructions, alongside DVC measurements of tissue and fibre deformation. In addition to the in situ experimentation, we have provided proof of concept of the technique through reconstruction of simple tendon (uniaxial fibre orientation) constructs, and demonstrated the scalability of the TomoSAXS technique to large samples through analysis of an intact human IVD.

The main advantages of this technique are: (1) the isolation of nanoscale properties of individual collagen fibres; (2) the estimation of per-fibre nanoscale strain through tracking the location of fibres in their unloaded and loaded states; and (3) the spatial mapping of nanoscale properties facilitating hierarchical structural and mechanical analysis from the nanoscale to macroscale. In addition, the low number of angular projections required for SAXS reconstruction enables scalability of the technique to intact human organs, and major advantages in terms of acquisition speed and X-ray dose compared with existing techniques.

Our analysis reveals new insights into hierarchical IVD mechanics, providing an important case study for the coupled nanoscale–microscale characterization of other collagenous tissues, such as arteries and cartilage, and engineered fibrous materials, such as carbon-fibre composites. TomoSAXS opens new avenues for linking collagen structure to function in health, disease, ageing and tissue engineering, and for advanced characterization of next-generation composites from biomedical to aerospace applications.

## Methodological overview of TomoSAXS

**Extended Data Fig. 1:**
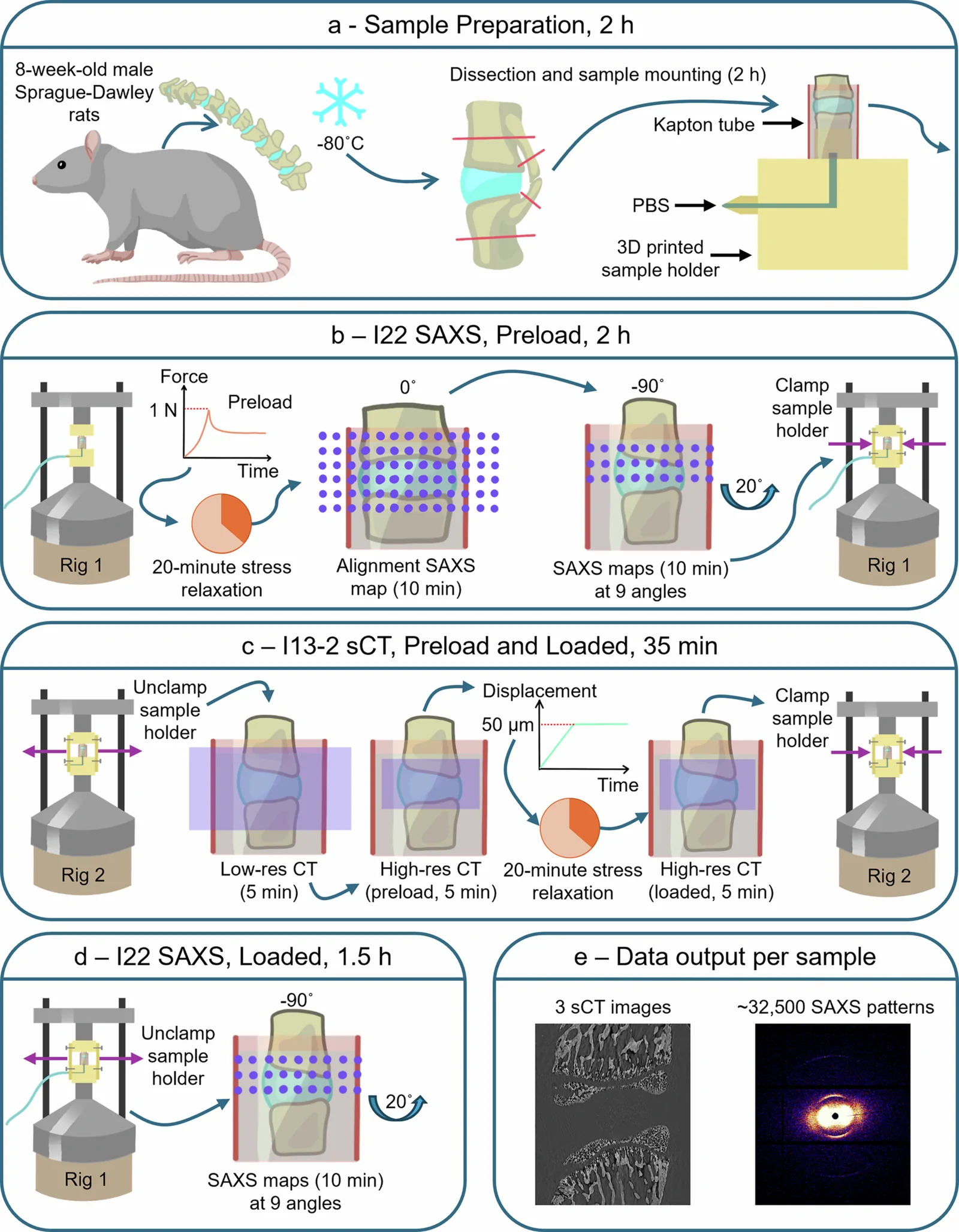
In situ *TomoSAXS* experimental method. Illustrations of: **a**, sample preparation and sample environment; **b**, preload application and SAXS scanning; **c**, pCT scanning before and after compressive displacement; **d**, post-loaded SAXS scanning; and **e**, summary of experimental data output per sample.

To obtain multiscale biomechanical analysis of IVDs under physiologically relevant load levels, correlative pCT and single-axis tomographic SAXS were performed at the Diamond Light Source synchrotron on three intact rat IVD samples before and after the application of 50-μm compressive displacement (Methods). An illustration of the experimental method is shown in Extended Data Fig. 1. TomoSAXS requires the indexing of fibres and characterization of their orientations from a 3D image, achieved here through automated fibre tracing of the initial pCT image^4^ (Methods; Fig. 1c). This orientation data, alongside rotational SAXS sampling and spatial registration of pCT and SAXS datasets (Methods), is key to deconvoluting the individual contributions of fibres across SAXS X-ray beam paths, as SAXS signals are orientation dependent. Unlike SAXS tensor tomography (SAXS-TT)^28^, in which multiple tomographic axes are required for a full reconstruction, TomoSAXS uses a single axis for SAXS tomography. The isolation of individual fibres from pCT enables the reconstruction of SAXS parameters on a per-fibre basis, allowing spatial mapping of nanoscale properties at the resolution of the pCT images (here 1.6-µm voxel size and 5.5-µm resolution^22^) as opposed to being limited to the SAXS beam size (here 20 µm). This is vital for complex fibrous materials such as the annulus fibrosus, where rapid angular changes occur at the microscale. Fibre tracing also minimizes the need for repeated angular sampling during SAXS scanning (resulting in considerable X-ray doses to biological samples^33^), and a single axis of rotation is applied to maximize the angular independence of scattering from different fibres rather than to estimate their orientation.

**Fig. 2:**
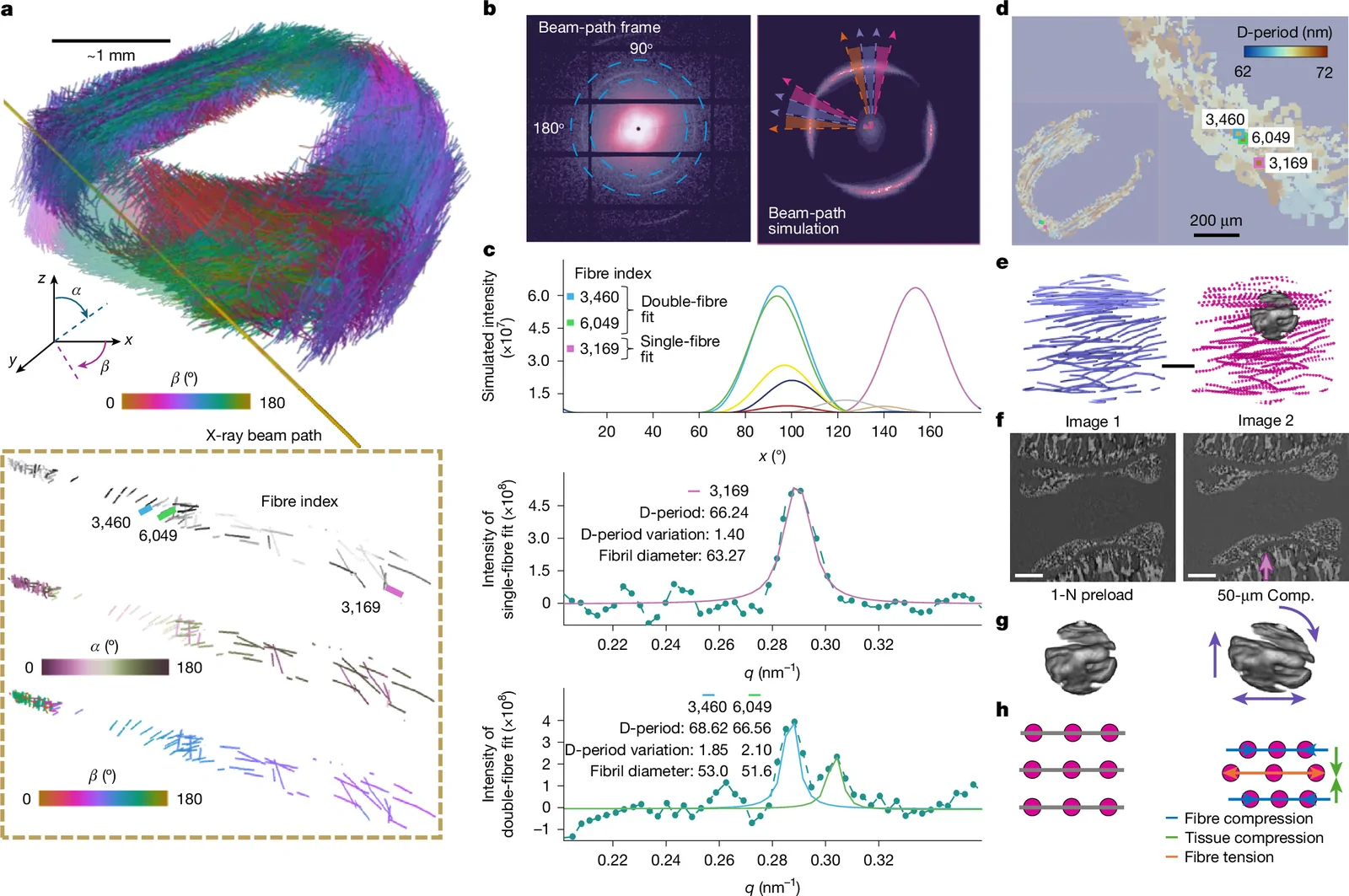
TomoSAXS reconstruction and fibre-based DVC. **a**, Complete fibre tracing for an IVD sample, with fibres coloured by orientation and one SAXS beam path rendered in gold (top); the fibre index for the three fibres predominantly contributing to the SAXS signal along the beam path (upper bottom); the *α*-orientation angles for the fibres within the beam path (middle bottom); and *β*-orientation angles for the fibres within the beam path (lower bottom). **b**, Resultant real (left) and simulated (right) SAXS frames for the beam path represented in panel **a**. **c**, Fitting and estimation of nanoscale fibril properties from isolated scattering regions. **d**, Spatial reconstruction of the per-fibre nanoscale properties in the pCT cross-section; the locations of the three fibres from panels **a**–**d** are indicated. **e**, Point cloud generation from fibre tracing, with the size of one sub-volume rendered in grey. Collagen fibres, purple, left; point cloud, pink, right. Scale bar, approximately 65 µm. **f**, pCT images after 1-N preload (left) and 50-µm compressive displacement (Comp.; right). Scale bars, 300 µm. **g**, 3D rendering of a sub-volume used for DVC tracking between images (left), and sub-volume after translation, rotation and the first order strain to match the deformed image (right). **h**, Illustration of the point cloud before (left) and after (right) compression, demonstrating different modes of tissue and fibre deformation.

A typical SAXS beam path contains multiple collagen fibres with varying orientation (Fig. 2a); however, owing to their 3D orientation and anisotropic diffraction, as well as their distance from the centre of the Gaussian beam profile, only a subset of these fibres will satisfy the Ewald diffraction condition and visibly contribute to the SAXS pattern (Fig. 2b,c). The first step of the TomoSAXS reconstruction uses singular-value decomposition of 3D fibril diffraction modelling to estimate the scattering amplitude of individual fibres in each beam path (Fig. 2c), using predicted mean values for physical properties to provide an initial amplitude estimation (Methods).

Next, the 3D orientation and estimated scattering amplitude of fibres are used to perform an iterative simulation of the SAXS tomography and isolate angularly independent scattering instances (Methods; Fig. 2c, top panel). By fitting SAXS profiles with 3D diffraction models^1^, the physical parameters fibril D-period, D-period variation and fibril diameter (Fig. 1d) can be obtained for each fibre (Methods; Fig. 2c, middle and bottom panels). Reconstruction follows a cascading pattern, in which the solution of individual fibres enables further deconvolution of neighbouring fibres, allowing an iterative sweep through the sample until every possible fibre has been fitted. Fibril properties can then be mapped onto the pCT image, enabling assessment of spatial variation and gradients in nanoscale properties (Methods; Fig. 2d).

The measurement of strain from per-fibre nanoscale properties requires accurate tracking of fibre deformation during in situ experimentation; achieved here with DVC analysis^4,21,22,24^. DVC analysis was performed on pCT datasets to measure the deformation of collagen fibres in the annulus fibrosus following axial compression using a local, sub-volume based approach, with measurement points placed along the traced fibres (Methods; Fig. 2e,f). Application of DVC-measured displacements to traced fibres in their undeformed state allows calculation of their location and orientation following loading, while preserving fibre index. This information is used to reconstruct SAXS data of loaded fibres and compare resultant fibrillar parameters across loading to generate nanoscale strain values. Fibre-level and tissue-level strains can also be obtained from the DVC-measured displacements (Methods; Fig. 2f).

## Validation and uncertainty of TomoSAXS

**Extended Data Fig. 2:**
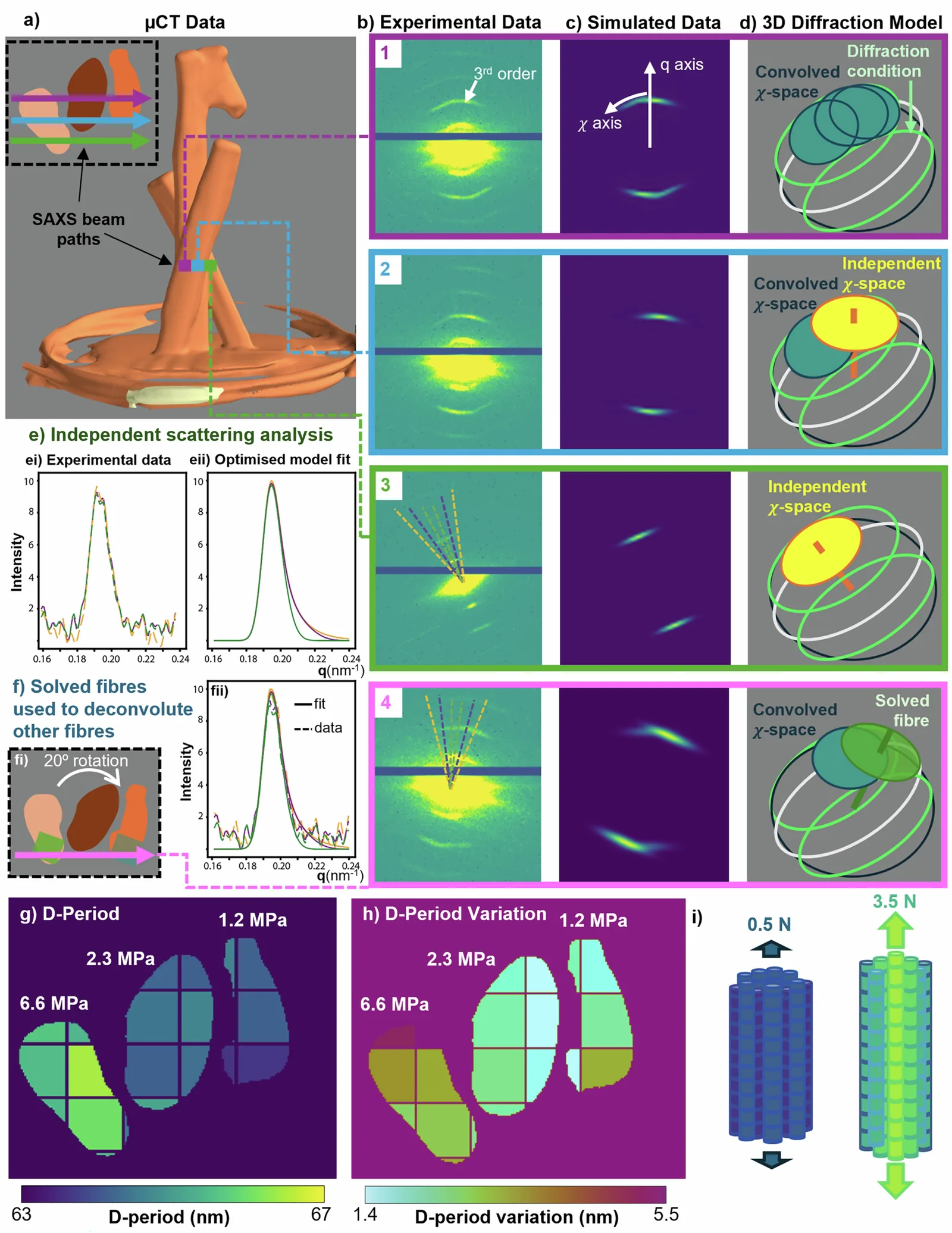
TomoSAXS validation on a simple, known system. **a**, 3D rendering of laboratory microCT data from 3 dried chicken foot tendons placed at different angles, with locations of example SAXS beam paths 1, 2, and 3 shown. **b**, Experimental laboratory SAXS data. **c**, Simulated 3rd order collagen peak SAXS data. **d**, 3D diffraction modelling for 4 X-ray beam paths. **e**, Independent scattering analysis of beam path 3; **ei**, experimental intensity data along the q axis; **eii**, optimized 3D diffraction model fit to the experimental data (accounts for peak skew along q). **f**, Results from the independent fibre in beam path 3 used to deconvolve information for the second fibre in beam path 4; **fi**, illustration of beam path 4 which is taken after rotating the sample 20°; **fii**, fit to experimental data for the convolved fibre. **g**, Collagen D-period and **h**, collagen D-period variation mapped across the 3 tendons. **i**, Illustration of collagen fibres in tendons dried under different tare loads - the tendon dried under 6.6 MPa tensile stress has a higher D-period and higher D-period variation compared to the tendon dried under 1.2 MPa stress.

To establish and validate the fundamental principles of the TomoSAXS reconstruction method, initial validation experiments were performed on a simple construct of dried chicken foot tendons using laboratory X-ray sources. Three tendons were dried under different levels of tensile load to create uniformly oriented bundles of collagen fibres with different D-periods (Methods; Extended Data Fig. 2). The results showed an increased D-period and D-period variation in the tendon dried under the highest tensile strain (Extended Data Fig. 2g,h), fibril strains of 0.6–2.8% and an effective fibril modulus of approximately 0.2 GPa, in the same range as previous single-fibril measurements^34^, demonstrating the ability of TomoSAXS to resolve variations in nanoscale collagen properties in a known system.

**Extended Data Fig. 3:**
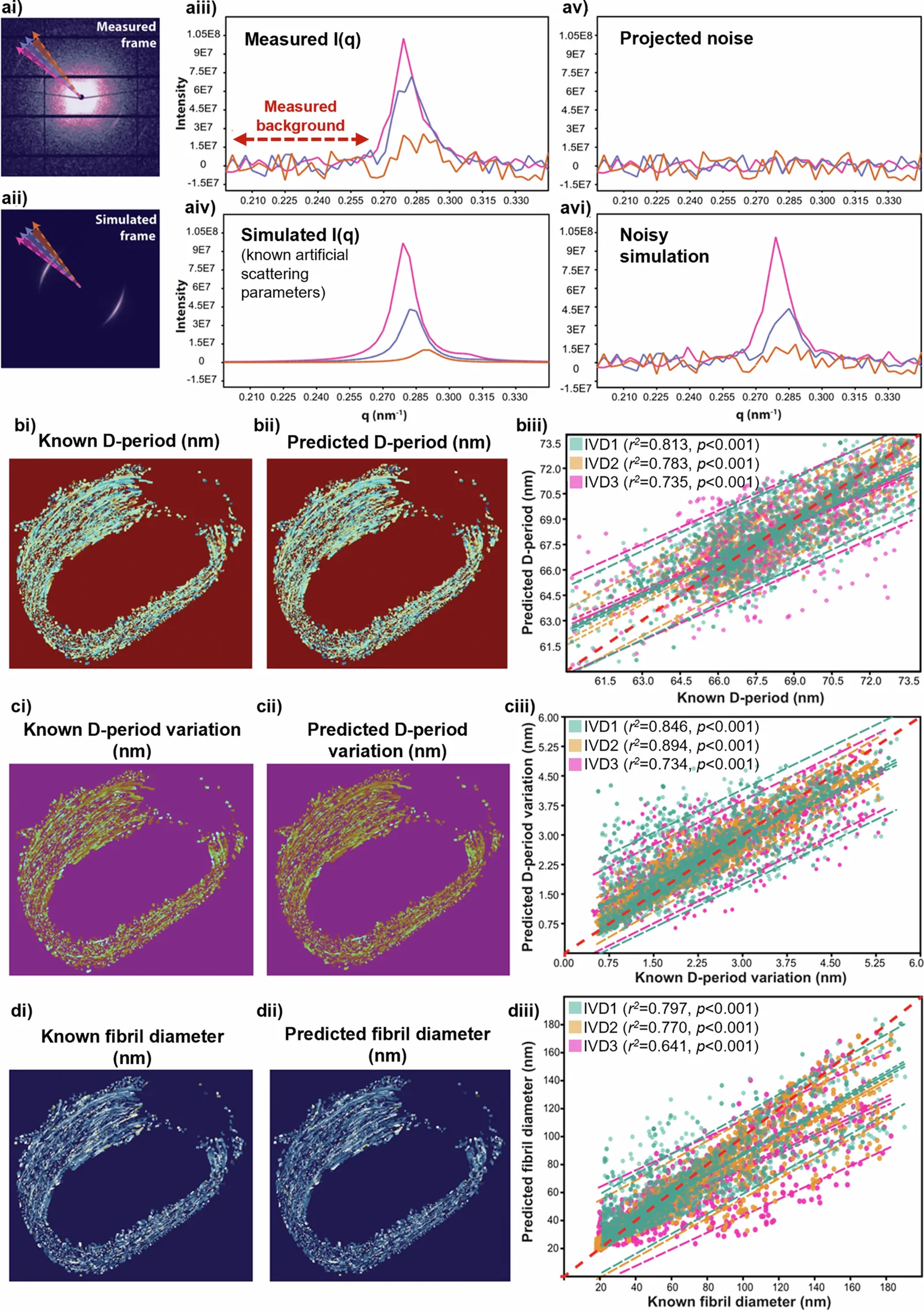
Application of digital phantom data to sample geometries for estimation of accuracy and precision of the *TomoSAXS* technique. **a**, Schematic illustrating simulation of scattering in a measured frame using known, artificial scattering parameters (retaining fibre index, orientation and estimated scattering amplitude) and application of noise from measured frame. **b**, D-period, **c**, D-period variation, and **d**, fibril diameter, **di**, 2D maps of known values, **dii**, 2D maps of predicted values, and **diii**, two-sided linear regression plots displaying the spatial and statistical accuracy of reconstructions. **biii**, Known D-period versus predicted D-period (nm) for IVD1 (*n* = 1806; *r*^2^ = 0.813; *p* = 0.0001); IVD2 (*n* = 654; *r*^2^ = 0.783; *p* = 0.0001); and IVD3 (*n* = 211; *r*^2^ = 0.735; *p* = 0.0001). **ciii**, Known D-period variation versus predicted D-period variation (nm) for IVD1 (*n* = 1806; *r*^2^ = 0.846; *p* = 0.0001); IVD2 (*n* = 654; *r*^2^ = 0.894; *p* = 0.0001); and IVD3 (*n* = 211; *r*^2^ = 0.734; *p* = 0.0001). **diii**, Known fibril diameter versus predicted fibril diameter (nm) for IVD1 (*n* = 1806; *r*^2^ = 0.797; *p* = 0.0001); IVD2 (*n* = 654; *r*^2^ = 0.770; *p* = 0.0001); and IVD3 (*n* = 211; *r*^2^ = 0.641; *p* = 0.0001). No adjustments were made for multiple comparisons. See Supplementary Note 18 for further discussion and presentation of alternative phantoms.

The accuracy of TomoSAXS for estimating nanoscale parameters in the rat IVD has been tested using digital phantoms (Extended Data Fig. 3). We created a series of digital IVD phantoms using indexed fibre geometries from three real samples and applying synthetic nanoscale fibrillar properties (Extended Data Fig. 3ai–ii). By applying noise in measured background scattering intensity from real SAXS frames (Extended Data Fig. 3aiii,av) to simulated signals under analysis (Extended Data Fig. 3aiv–vi), we estimated how accurately the TomoSAXS reconstruction reproduces the known parameters. We found significant correlation between reconstructed and ground-truth data (*P* < 0.001, *r*^2^ = 0.641–0.894). Accuracy, measured as the mean absolute error (Methods), was 1.032 nm for the mean D-period (6.8% of the 6σ width), 0.280 nm for D-period variation (4.2% of the 6σ width) and 8.532 nm for fibril diameter (6.9 % of the 6σ width). Precision, measured as the standard deviation of absolute error (Methods), was 1.37 nm for the mean D-period (9.05% of the 6σ width), 0.34 nm for D-period variation (5.1% of the 6σ width) and 15.32 nm for fibril diameter (6.9% of the 6σ width). Comparison of mapped known versus estimated properties also suggest that spatial patterns in known properties are reconstructed (Extended Data Fig. 3b–d). When provided noise-free data on identical geometries, TomoSAXS reconstruction accuracy improved, with *r*^2^ = 0.874–0.915, which demonstrates the theoretical limitations of the technique (Supplementary Fig. 8).

## Hierarchical fibre analysis

**Fig. 3:**
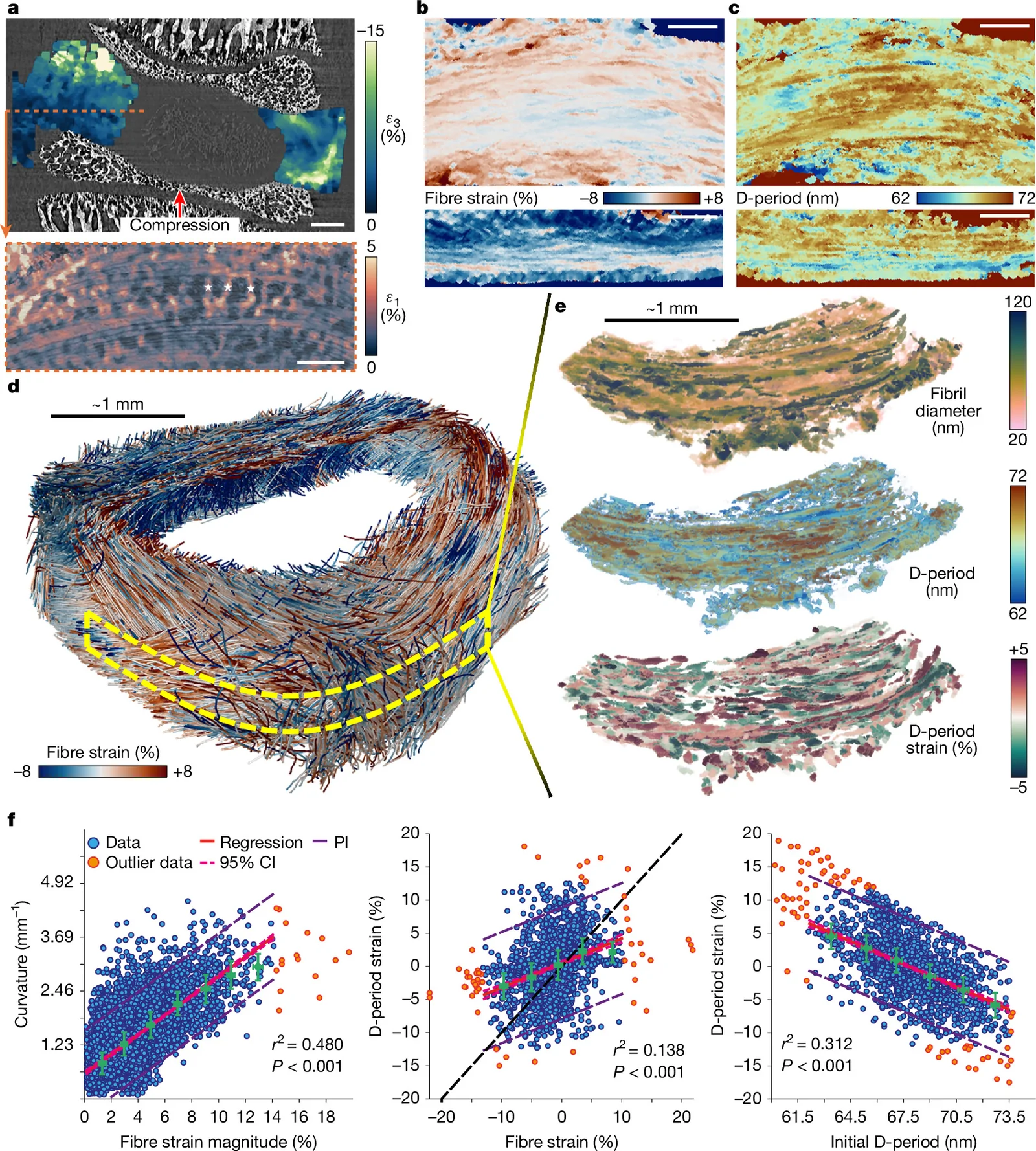
TomoSAXS reveals multiscale collagen structure–function relationships in whole IVDs. **a**, Tissue-level patterns in compression (third principal strain *ε*_3_; top) and tension (first principal strain *ε*_1_; bottom). **b**, Microscale fibre strain patterns in the anterior (top) and posterior (bottom) annulus fibrosis. **c**, Nanoscale patterns in the initial D-period showing anticorrelation with fibre strain patterns in panel **b**. Scale bars represent 300 µm. **d**, 3D reconstruction of fibre strain patterns through the entire annulus fibrosis. **e**, Maps of nanoscale properties found within the region in panel **d** marked with yellow dashed lines. **f**, Two-sided linear regression analysis of fibre curvature versus fibre strain magnitude (left; *n* = 9,326; *r*^2^ = 0.480; *P* = 0.0001); the fibrillar D-period strain versus the fibre strain (middle; *n* = 1,826; *r*^2^ = 0.138; *P* = 0.0010); and the fibrillar D-period strain versus the initial D-period (right; *n* = 1,826; *r*^2^ = 0.312; *P* = 0.0001). The pink dashed line denotes the 95% confidence interval (CI), and the purple dashed line indicates the prediction interval (PI). The green box plots represent standard deviations across both axes for evenly spaced subsamples across the *x* axis, centred around the mean of each respective subsample across both axes (**f**). No adjustments were made for multiple comparisons. All images are from a single representative IVD (IVD1).

**Extended Data Fig. 4:**
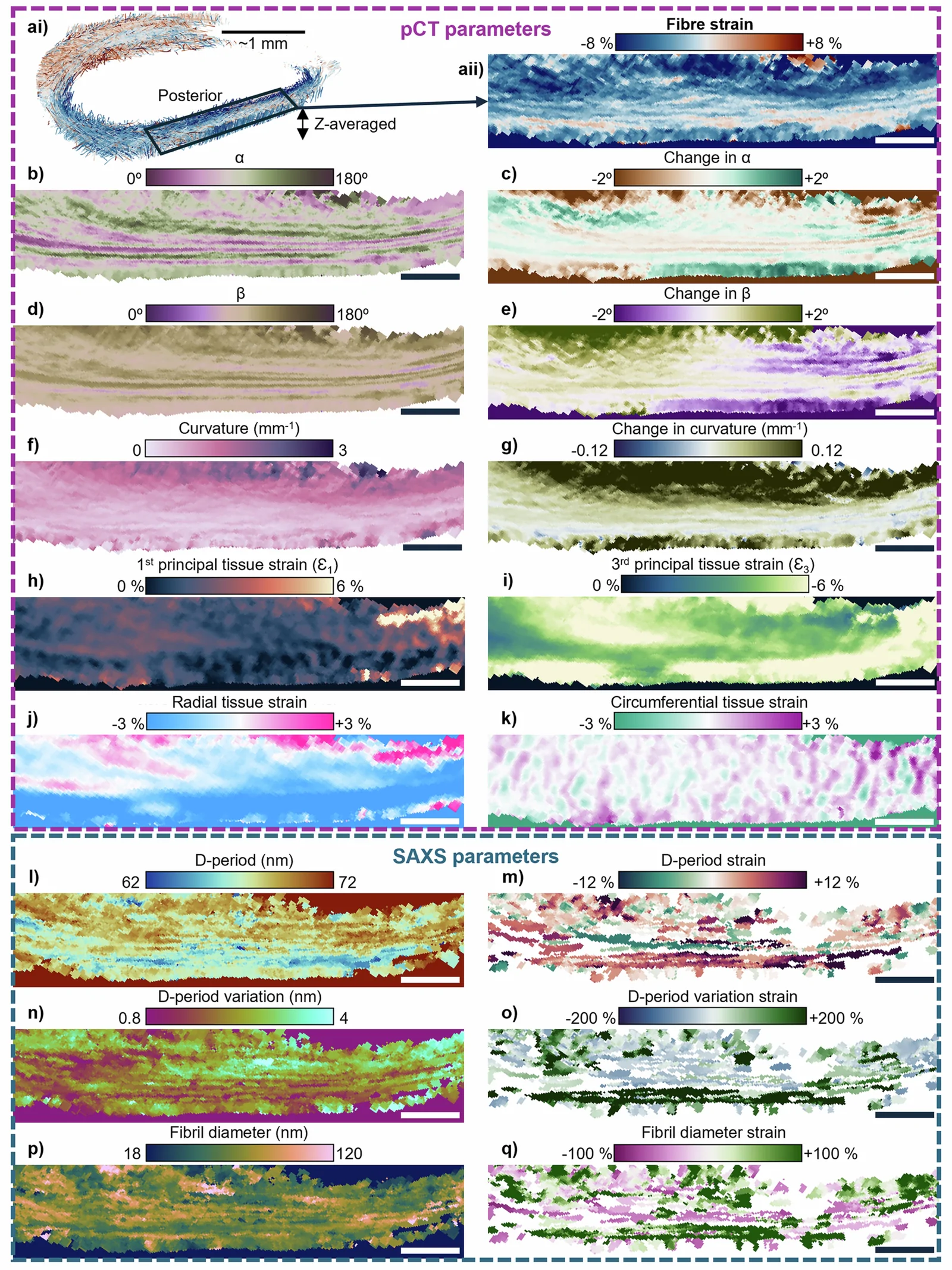
*TomoSAXS* enables correlative spatial mapping of nano- to microscale structure and mechanics. pCT parameters. **ai**, Fibre strain in the AF; **aii**, z-averaged fibre strain in the posterior region as highlighted in **ai**. Z-averaged spatial mapping of: **b**, vertical angle α; **c**, change in α with load; **d**, lateral angle β; **e**, change in β with load; **f**, fibre curvature; **g**, change in curvature with load; **h**, 1^st^ principal (maximum tensile) tissue strain; **i**, 3^rd^ principal (maximum compressive) tissue strain; **j**, radial tissue strain; **k**, circumferential tissue strain; **SAXS parameters:** **l**) per fibre mean D-period; **m**, per fibre mean D-period strain – the percentage change in D-period measured before and after loading; **n**, per fibre D-period variation; **o**, per fibre D-period variation strain; **p**, fibril diameter; **q**, fibril diameter strain. All data shown are from a single IVD (IVD1).

**Extended Data Fig. 5:**
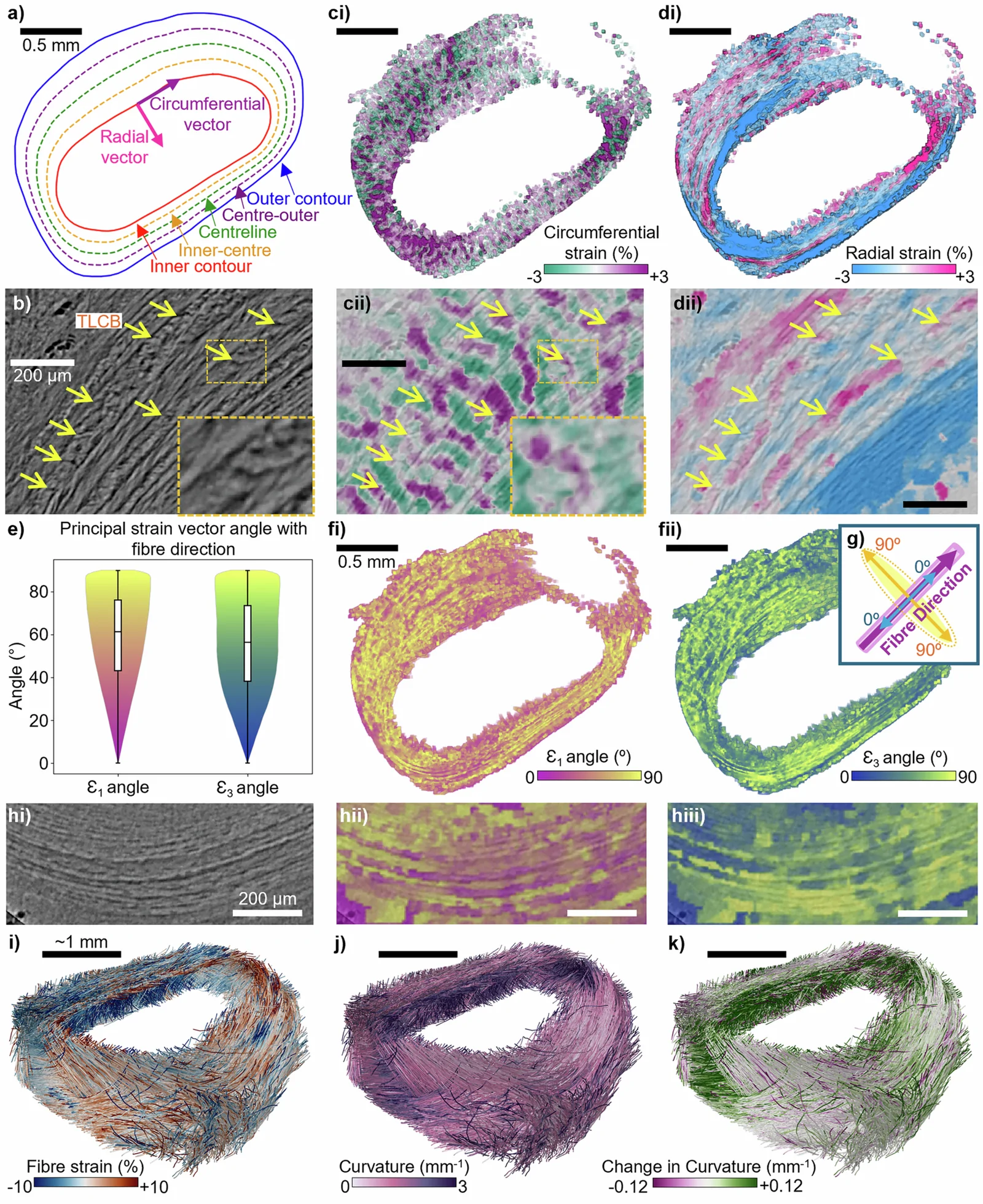
Tissue level strain analysis and the link between fibre strain and curvature. **a**, Contour lines used for calculating tissue-level radial and circumferential strains (Supplementary Note 6). **b**, Greyscale image of lamellae in the anterior AF, with trans-lamellar cross-bridges (TLCBs) highlighted by yellow arrows. **c**, Circumferential and **d**, radial strain in the AF. **e**, Violin plots of 1^st^ and 3^rd^ tissue level principal strain angles to the local fibre direction. **f**, 3D renderings of i) 1^st^ and ii) 3^rd^ principal strain angle with the local fibre direction. **g**, Illustration of the angles used for defining principal strain vector angle with the local fibre direction; **hi**, greyscale image of the posterior-lateral AF with **hii**, 1^st^ principal strain angle and **hiii**, 3^rd^ principal strain angle displayed as a colourwash, showing clear lamellar texturing. 3D renderings of fibres in the AF coloured by **i**, fibre strain; **j**, curvature; and **k**, change in curvature upon compressive loading. All data are from a single IVD (IVD1).

TomoSAXS correlates 3D microscale imaging with nanoscale measurements from SAXS, enabling 3D mapping and direct statistical comparison of per-fibre microscale and nanoscale properties in macroscopic samples. Here application of TomoSAXS resulted in 3D maps of structural and mechanical properties specific to individual collagen fibres across the spatiostructural hierarchy of the annulus fibrosus (Fig. 3 and Extended Data Figs. 4 and 5). The orientational texture of our samples and applied experimental parameters (Methods) permitted nanoscale reconstruction for more than 55% of all fibres within the scanned volume (Supplementary Fig. 6). This provided sufficient resolution to highlight complex textural patterns in both structural and mechanical nanoscale properties (Fig. 3c,e and Extended Data Fig. 4l–q).

An estimated 100-nm accuracy in DVC fibre-displacement tracking (Methods) allowed confident estimations of displacement under load for individual fibres, enabling precise per-fibre nanoscale D-period and fibril diameter strain measurements (Fig. 3e, bottom panel, and Extended Data Fig. 4m,o,q). The nanoscale measurements of structure and mechanical response can then be directly compared with those at the fibre and tissue levels (Fig. 3 and Extended Data Fig. 4).

## Matrix mechanics in the annulus fibrosus

At the highest level (Fig. 3a), local tissue strains measured from the DVC were overall higher than the bulk strain applied to the IVD, measured as the percentage change in disc height (Methods; Supplementary Note 8). A reduction in IVD height of 2.2% resulted in tissue-level principal compressive strains of 4.2 ± 3.3% in the annulus fibrosus^28^ (Fig. 3a, top panel); local tissue strains higher than the overall applied strain have been observed in other hard-soft tissue junctions^2^.

Maps of tissue-level first principal (tensile) strain reveal both lamellar and trans-lamellar patterns (Fig. 3a, bottom panel). Projecting tissue-level strain along the circumferential direction (Extended Data Fig. 5a) shows alternating trans-lamellar bands of tension and compression (Extended Data Fig. 5c). We hypothesize that these strain patterns may be related to the trans-lamellar cross-bridging network of the annulus fibrosus (Extended Data Fig. 5b), as previously suggested^21^, which is thought to aid in returning annulus fibrosus tissue to its original shape after deformation^20^. Because strains were recorded after stress relaxation, these trans-lamellar patterns may be a result of elastic recoil from the inter-lamellar matrix. Such patterns suggest that trans-lamellar cross-bridges may be an initiation site for annular tears, providing support for previous hypotheses^35^. Spatial mapping of strain in the radial direction shows clear radial compression of the annulus fibrosus due to bulging of the nucleus pulposus (Extended Data Fig. 5d), with some lamellae exhibiting radial tension, possibly due to swelling induced by fluid flow out of the nucleus pulposus into the annulus fibrosus^36^.

Tissue-level principal strain direction, measured as the angle between the strain eigenvector and the local fibre tangent (Extended Data Fig. 5g), averaged 55 ± 22° for compression and 58 ± 21° for tension (Extended Data Fig. 5e). Fibres resist tensile strains aligned with their orientation but provide little resistance to strain in other directions. The lack of alignment between tissue-level principal strains and fibre orientation therefore indicates substantial strain accommodation by the extrafibrillar matrix and inter-fibre motion. Spatial mapping of principal strain directions shows clear lamellar texturing, with alternating lamellae demonstrating tissue-level principal strains acting parallel or orthogonal to the fibre direction (Extended Data Fig. 5f,h).

## Fibre curvature correlates with strain

Fibre-level strain magnitude showed a significant positive correlation with fibre curvature (*r*^2^ = 0.480, *P* < 0.001; Fig. 3f, left panel, and Extended Data Fig. 5i,j). Fibres with the greatest curvature were typically located at the transition zone between the nucleus pulposus and annulus fibrosus in the posterior and lateral regions of the IVD (Extended Data Fig. 5j), with these fibres showing a tendency to increase in curvature under compressive loading of the IVD (Extended Data Fig. 5k). The link between fibre curvature and fibre strain may partially explain why the posterior and posterior–lateral regions of the IVD are most prone to herniation^37^.

## Fibril pre-strain drives fibre mechanics

Fibre strain measured from the DVC showed a significant positive correlation with fibrillar D-period strain measured from SAXS (*r*^2^ = 0.138, *P* < 0.001; Fig. 3f, middle panel). A mean fibre strain magnitude equal to 3.70% (±3.19%) and mean fibrillar strain magnitude equal to 3.60% (±3.36%) suggests that most of the strain at the fibre level is passed down to the fibril, with limited inter-fibril sliding at this level of loading. However, the slope of the regression line deviates from one due to a distinct subpopulation of fibres with near-zero D-period strain, which experience a considerably higher fibre strain magnitude (Fig. 3f, middle panel). This may be evidence of previously reported lags in fibril strain response to low-strain conditions due to molecular tilting in twisted collagen fibres^38^.

Fibrillar D-period strain showed a significant negative correlation with the initial fibrillar D-period (*r*^2^ = 0.312, *P* < 0.001; Fig. 3f, right panel). This can be interpreted as IVD tissues with fibres under initial tensile pre-strain undergoing compression upon loading, and those with an initial compressive pre-strain undergoing tension. Although it seems counterintuitive, at the nanoscale, where strain is measured over lengths of approximately 67 nm in fibrils with diameters of 20–120 nm, collagen fibrils can support compressive loading, with the surrounding extrafibrillar matrix providing lateral support that suppresses buckling.

The initial fibrillar D-period covered a broad range of values: from 60 nm to 74 nm. Assuming a zero-strain state of approximately 67 nm, this represents residual fibrillar strains ranging from −10% to +10% in the IVD. Given a Young’s modulus of approximately 1–2 GPa for hydrated type I collagen fibrils^34^, our data translate to a measured pre-stress of 100–200 MPa. The measured failure stress of single collagen fibrils from rat tendons range from 70 to 200 MPa^39^, indicating that residual stress in the IVD may result in fibrillar strains close to failure. This may help to explain why the annulus fibrosus is susceptible to microdamage at mechanical loads well below those required to induce failure in the disc^40^. Similar high pre-stresses have been reported before for tendon collagen and mineralized collagen fibrils^41,42^, and may be required to adapt material stiffness to meet the challenging mechanical requirements of the IVD. The regression line for the initial D-period versus the D-period strain shows that D-period strain values typically return the fibril D-period towards its zero-strain state of 67 nm.

**Extended Data Fig. 6:**
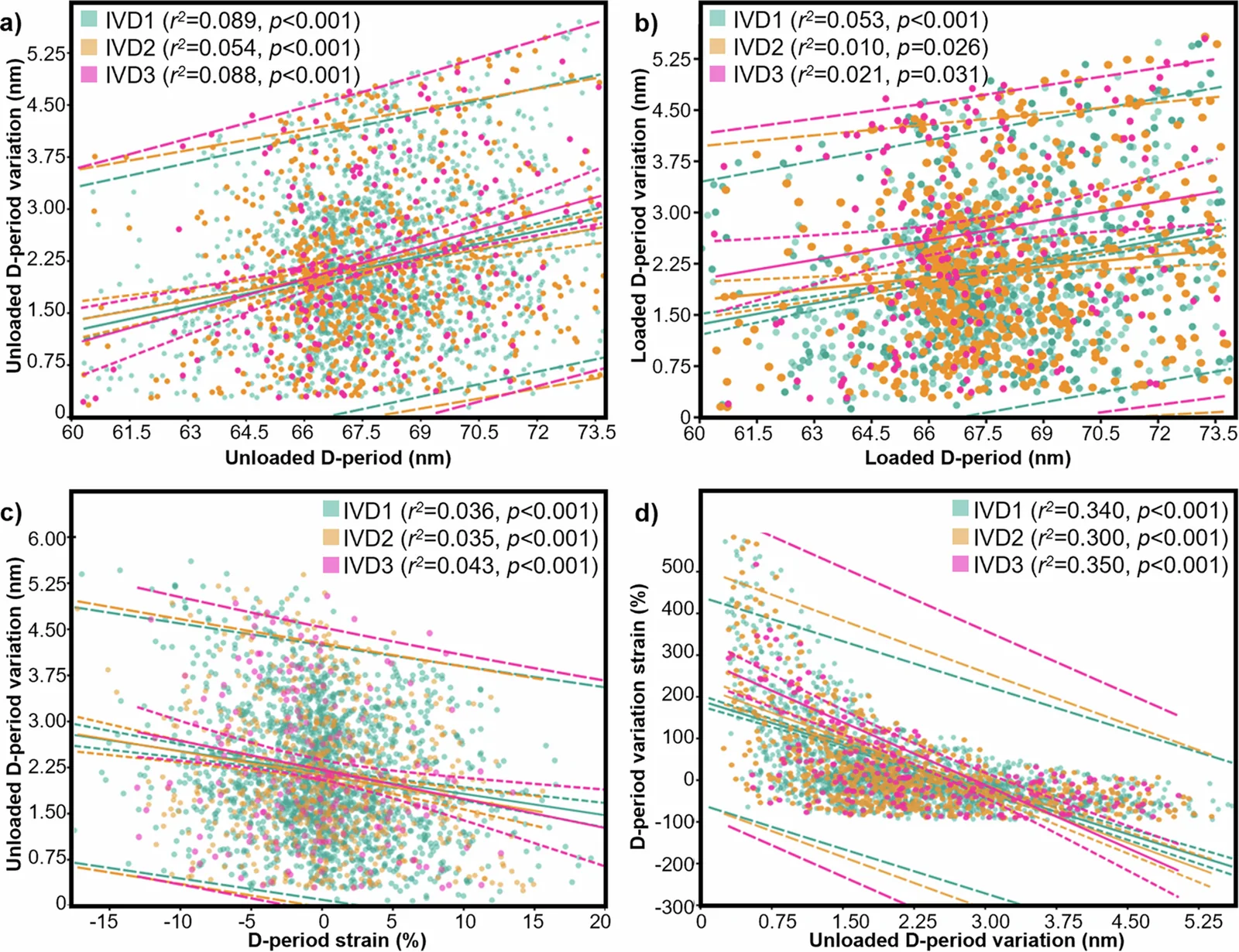
Statistical relationships for three IVD samples (Supplementary Table 1), characterized using two-sided linear regression. **a**, Unloaded mean per-fibre D-period versus variation in unloaded per fibre D-period (nm) for IVD1 (*n* = 1826; *r*^2^ = 0.089; *p* = 2.227*10^−23^), IVD2 (*n* = 670; *r*^2^ = 0.054; *p* = 8.917*10^−7^), and IVD3 (*n* = 220; *r*^2^ = 0.088; *p* = 7.722*10^−6^). **b**, Loaded per-fibre D-period versus variation in loaded D-period (nm) for IVD1 (*n* = 1826; *r*^2^ = 0.053; *p* = 2.713*10^−19^), IVD2 (*n* = 670; *r*^2^ = 0.010; *p* = 0.026), and IVD3 (*n* = 220; *r*^2^ = 0.021; *p* = 0.031). **c**, Per-fibre D-period strain (%) versus unloaded D-period variation (nm) for IVD1 (*n* = 1826; *r*^2^ = 0.036; *p* = 2.824*10^−12^), IVD2 (*n* = 670; *r*^2^ = 0.035; *p* = 1.100*10^−6^), and IVD3 (*n* = 220; *r*^2^ = 0.043; *p* = 0.001). **d**, Unloaded D-period variation (nm) versus per-fibre D-period variation strain (%) for IVD1 (*n* = 1826; *r*^2^ = 0.340; *p* = 1.716*10^−123^), IVD2 (*n* = 670; *r*^2^ = 0.300; *p* = 8.999*10^−52^), and IVD3 (*n* = 220; *r*^2^ = 0.350; *p* = 3.408*10^−18^). No adjustments were made for multiple comparisons.

The mean fibrillar D-period demonstrated a significant positive correlation with intra-fibrillar variation in the D-period (*r*^2^ = 0.089, *P* < 0.001; Extended Data Fig. 6a,b). Fibrils with larger mean D-periods, which can be considered as having a tensile pre-strain, have greater variability in the D-periods of their constituent microfibrils. We also found a significant negative relationship between initial per-fibre D-period variation and change in variation upon load (*r*^2^ = 0.340, *P* < 0.001; Extended Data Fig. 6), suggesting that fibres undergoing compression become less variable in the D-period and the inverse for those undergoing tension.

## Lamellar texture in fibril properties

**Extended Data Fig. 7:**
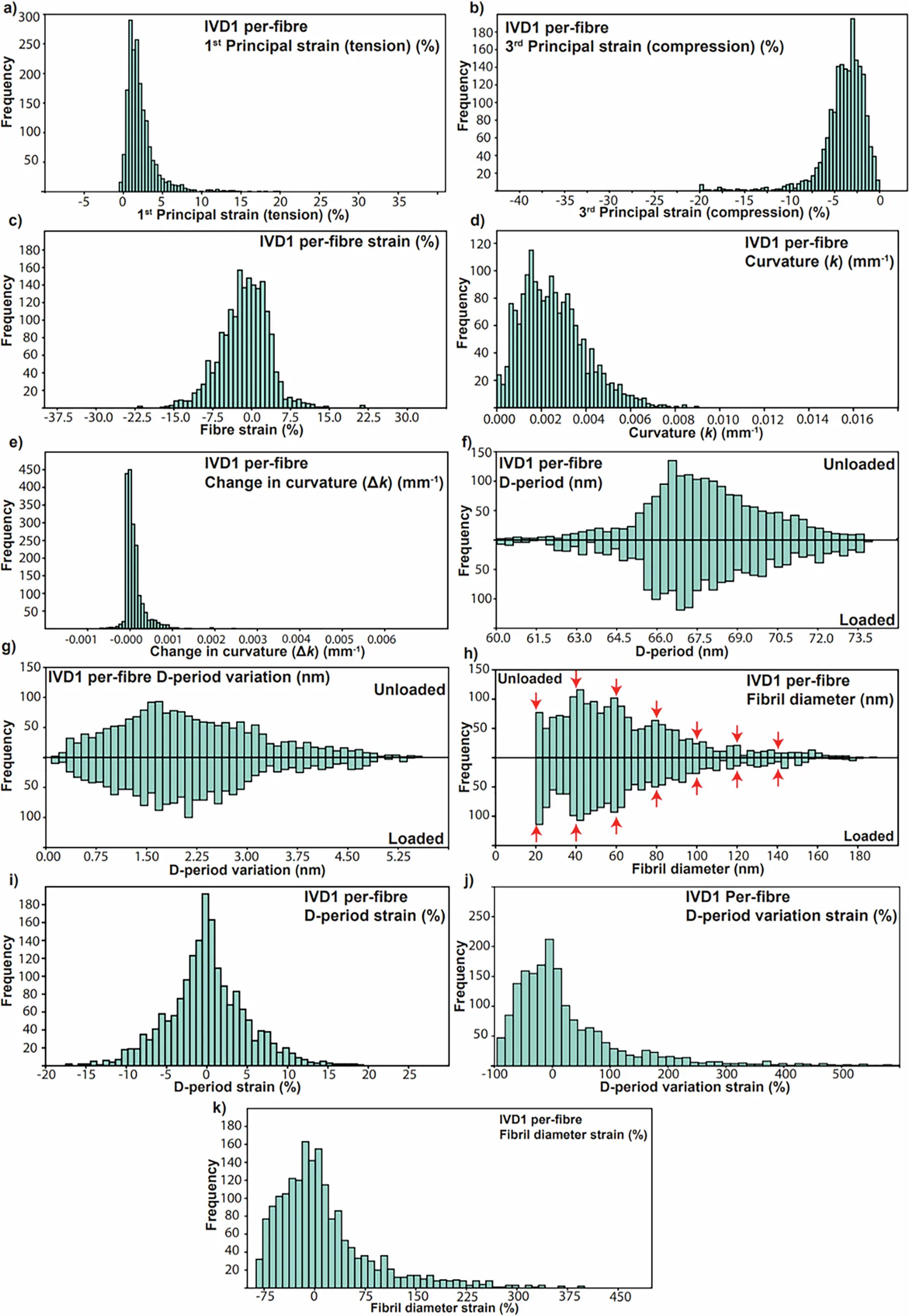
Histograms displaying variation in estimated per-fibre. **a**, 1^st^ principal strain. **b**, 3^rd^ principal strain. **c**, Fibre strain. **d**, Curvature. **e**, Change in curvature. **f**, D-period. **g**, D-period variation. **h**, Fibril diameter. **i**, D-period strain. **j**, D-period variation strain. **k**, Fibril diameter strain for sample IVD1.

Global and spatial patterns in structural and mechanical properties at the fibril to tissue level can be analysed by the spatial registration of TomoSAXS datasets. The mean fibril diameter exhibits twofold or larger variability across the tissue (64.4 ± 32.5 nm), with a clear lamellar texture (Fig. 3e, top panel). Closer inspection of fibril diameter distribution (Extended Data Fig. 7h) reveals multiple peaks on a base harmonic of approximately 20 nm. This aligns with previous electron microscopy studies^43^ revealing type II collagen fibrils form different super-coiled structures from a building block of repeated approximately 20-nm microfibrils. Combined with the texture seen in Fig. 3e (top panel), these results suggest that super-coiling levels may be different in different regions of the annulus fibrosus and between lamellae.

**Extended Data Fig. 8:**
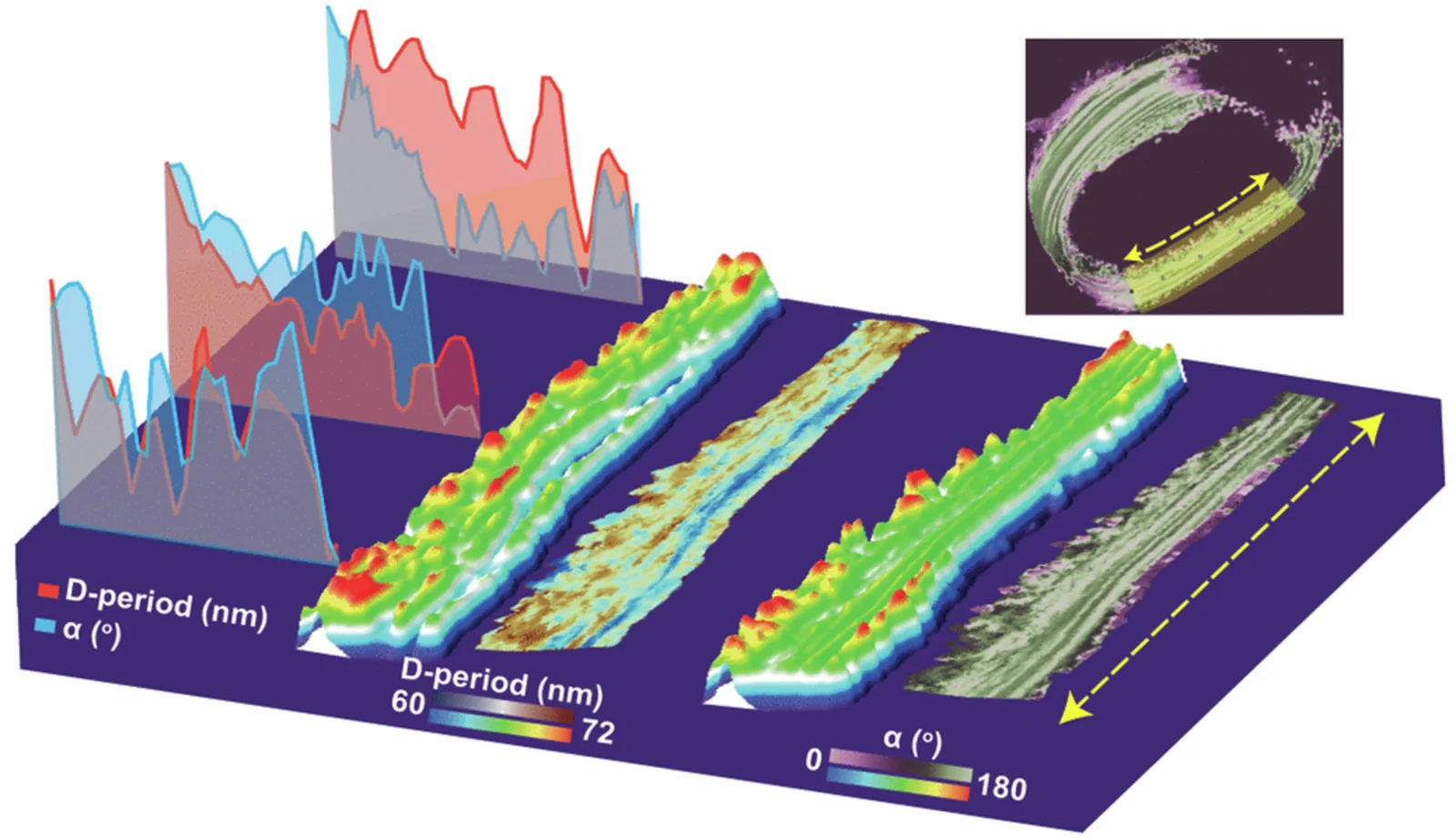
Transect analysis and comparison between D-period patterning and vertical fibre orientation (α) across algorithmically straightened samples of the posterior IVD1.

Algorithmic straightening of maps^44^ for fibrillar-level properties permits comparison of their spatial patterns across individual transects through the annulus fibrosus via line profiles, providing quantitative support for correlations qualitatively visualized between fibril characteristics and their lamellar texture (Extended Data Fig. 8). Of note, comparisons of the D-period pre-strain and fibre orientation (*α*) across shared transects through the posterior annulus fibrosus suggest that pre-strain is broadly conserved within individual lamellae, and neighbouring lamellae show contrasting pre-strains suggestive of complementary fibrillar pre-stress (Extended Data Fig. 8).

## TomoSAXS for intact human organs

**Fig. 4:**
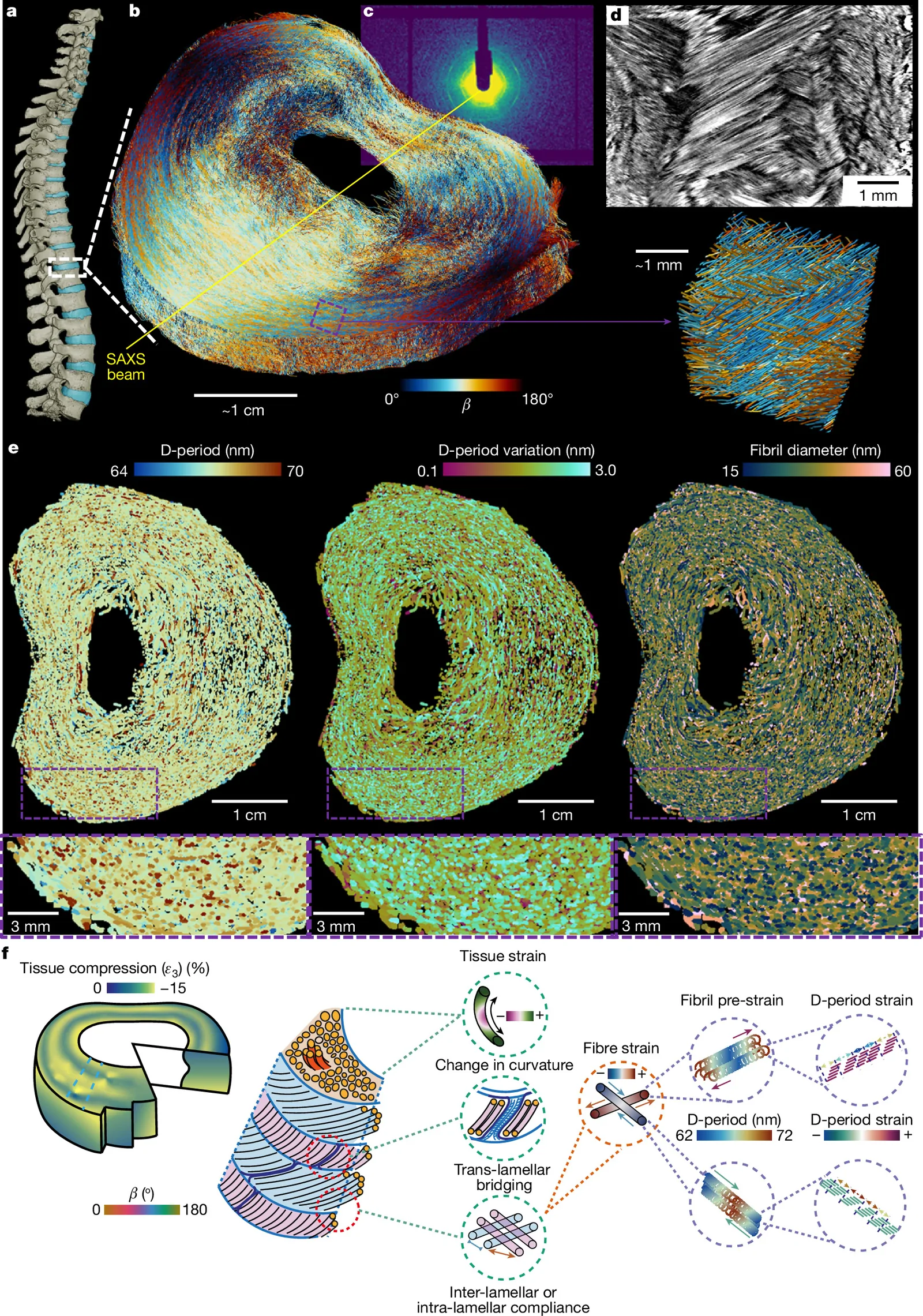
TomoSAXS of human IVD and hierarchical IVD mechanics. **a**, Complete human spine. **b**, Fibre tracing generated from HiP-CT of an intact human IVD (thoracic level 11–12, 64-year-old male donor). **c**, High-energy SAXS pattern. **d**, Greyscale HiP-CT image showing a clear fibre texture (top) and a zoomed-in image of a region of fibre-tracing data (bottom). **e**, Single-slice TomoSAXS reconstruction showing the D-period (left), D-period variation (middle) and fibril diameter (right) across the IVD. **f**, Schematic outlining the mechanisms of load transfer isolated here from the macrolevel or whole-tissue level, to the nanoscale.

We demonstrated the potential of the TomoSAXS technique on human samples, by performing correlative hierarchical phase-contrast tomography (HiP-CT)^45^ and tomographic SAXS at high energies on a whole formalin-fixed IVD (approximately 5 cm width) at The European Synchrotron. A single-slice reconstruction of the data shows clear texturing of nanoscale properties (Fig. 4e). The measured D-period ranged from 62.38 to 73.80 nm with a mean value of 67.15 nm (standard deviation = 0.98 nm), D-period variation ranged from 0.175 to 3.07 nm with a mean value of 1.56 nm (standard deviation = 0.61 nm) and fibril diameter ranged from 15.67 to 117.78 nm with a mean value of 41.29 nm (standard deviation = 17.08 nm). This represents an order of magnitude increase in sample dimensions probed using 3D SAXS techniques and opens doors for the investigation of matrix drivers of health and disease in intact human organs.

## Discussion and outlook

The TomoSAXS methodology provides an unparalleled view of macroscale-to-nanoscale mechanics in the IVD (Fig. 4c). By embedding SAXS data within the structural and mechanical context from pCT and DVC, it enables analysis of nanoscale–tissue interactions within the hierarchical assembly of the IVD. Tissue-level strains show complex patterns, probably linked to non-collagenous annulus fibrosus components such as elastin, thought to enable recoiling of the annulus fibrosus after deformation^20^. Fibre-level strains showed a strong correlation with fibre curvature, indicating that highly curved regions may be more prone to injury.

**Extended Data Fig. 9:**
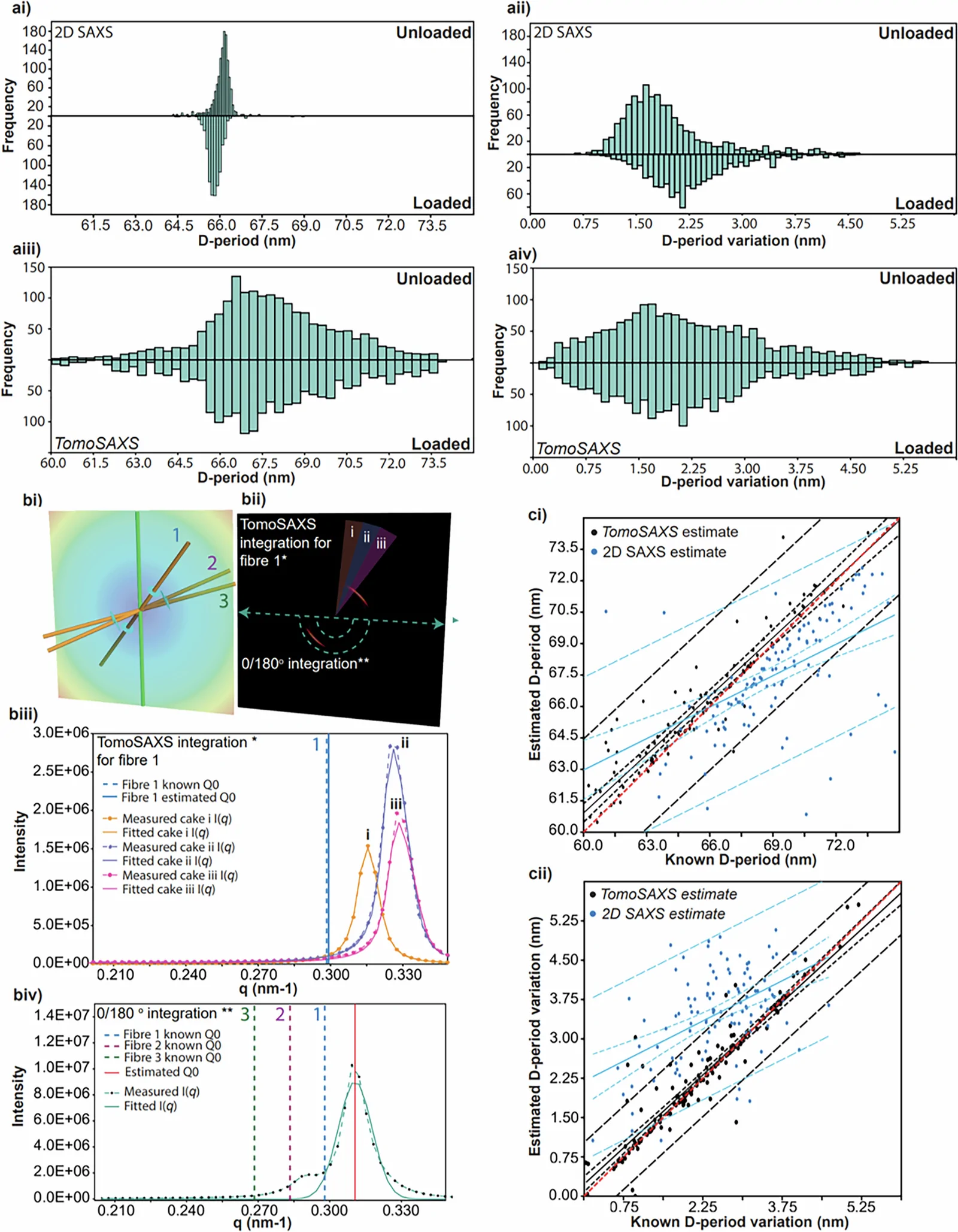
Comparison between performance using TomoSAXS versus traditional 2D SAXS approaches for studying nanoscale structure in IVD. **a**, Comparison between estimates of mean D-period and D-period variation for sample IVD1 using 2D SAXS (above) and TomoSAXS (below) analyses of IVD samples. **b**, Comparison between 2D SAXS and TomoSAXS analysis of single voxels modelled using 3D diffraction models, consisting of 3 fibrils of known scattering properties taken from a random distribution with the reported range from real IVD samples (**bi**). **bii**, Comparison between TomoSAXS integration of independent scattering regions (coloured cakes i-iii for Fibre 1 in **bi**), and traditional 0–180° 2D SAXS azimuthal integration (dashed green lines). **biii**, TomoSAXS fitting of 3D diffraction models to radial integrations across each labelled cake in **bii** based on the 3D orientation of Fibre 1, alongside known q_0_ (nm^−1^: inverse to D-period) and estimated q_0_. **biv**, 2D SAXS fitting of combined scatter integrated from 0–180° across χ using a Gaussian model, alongside a single estimate of q_0_ using this model and known values for each of the three fibres in the voxel. **c**, Comparison between known and fitted D-period (**ci**) and D-period variation (**cii**) for 100 simulated fibres using TomoSAXS (black) and 2D SAXS (blue).

Fibrillar mechanics critically inform strain transmission across scales. Fibrillar pre-strains (initial D-period) and load-induced strain showed strong spatial relationships with fibre-level strains. Their absolute magnitudes suggest that most fibre strain is transferred directly to fibrils, with little inter-fibrillar shear, unlike tendons under tension^46^. Avoiding the averaging effect of commonly used 2D diffraction techniques for studying nanoscale strains and the convolution of scattering from fibres at differing orientations (Extended Data Fig. 9) may also contribute to the wider spread of fibril strains seen (up to approximately 10–15%), which are consistent with tensile tests of individual fibrils^39^.

A negative correlation between the initial D-period and D-period strain suggests pre-strained fibrils converge towards a neutral state under load. High fibrillar residual stresses position the IVD in a mechanically advantageous but vulnerable state. This correlates with clinically seen high levels of degeneration in the IVD^13–16^. Pre-strain stiffens an otherwise compliant tissue, enabling physiological load bearing while reducing the remaining margin before damaging strains are reached. This illustrates a fundamental trade-off in fibre-reinforced biological tissues, in which mechanical performance is achieved at the expense of increased susceptibility to microdamage and degeneration.

The relationship between mean and variance in per-fibre D-period supports a helical coil model of fibrillar mechanics, loosening or tightening under load (Fig. 4f), akin to previous twisted or coiled fibre models^38,47^. This suggests an increase in the variability of stress along fibres as they deviate from a purely compressive (lowest possible D-period) state (Fig. 4f). In this scenario, loading causes increased local swelling pressure to a fibril in a low D-period state, leading to localized regions of high tension (and higher D-period), a phenomenon that is amplified as overall osmotic pressure and residual strain increases. Loss of residual strain in the IVD leads to aberrant cellular mechanosensing and degeneration^48^; however, methods to measure fibrillar-level pre-strain in intact organs have been lacking until now.

The multiscale mechanisms of load response reported here may be incorporated into finite-element modelling of IVD biomechanics^49^ and explain complex non-linear load responses. Collagen fibrils store stress and strain energy through osmotic pressure shifts, generating hundreds of MPa during dehydration^42^. These correlations have potential clinical relevance in musculoskeletal degeneration, with reduction in swelling pressure due to synthetic ageing shown to result in a reduction of fibrillar pre-strain in articular cartilage^50^. The mean values and load-induced changes of nanoscale properties reported here conform to previously reported values in other collagenous soft tissues^1,3^; however, the range of nanoscale strains exceeds previous estimates^1,3^. By spatially deconvolving 3D pre-strain maps (Fig. 3), TomoSAXS surpasses previous 2D SAXS studies that have reported only depth-averaged changes. Indeed, owing to the range of fibre orientations and variable tissue-level strain in the IVD, conventional SAXS would have reported an average fibril strain of zero, missing the rich intra-tissue texture and multiscale correlations (Supplementary Note 19). Other soft tissues such as arteries exhibit a multi-layered structure with variable fibre orientation and internal pre-stress; changes in ultrastructural pre-strain at the fibrillar level may also have important biomechanical roles in such systems^51,52^.

**Extended Data Fig. 10:**
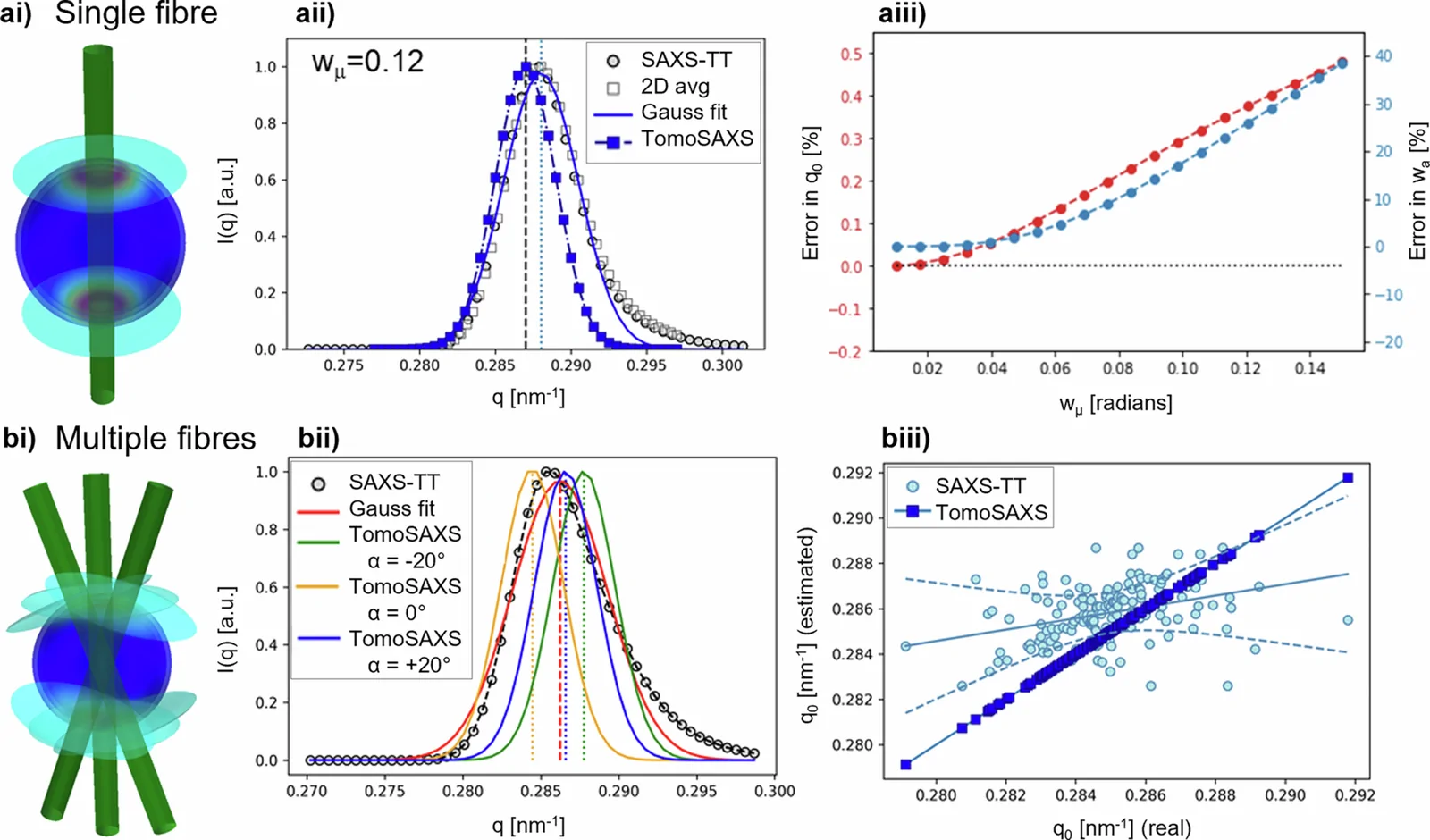
Comparison between SAXS tensor tomography (SAXS-TT) and TomoSAXS for reconstructing scattering parameters of individual fibres. **ai**, 3D rendering of reciprocal space intensity for a single vertically oriented fibril showing the meridional peak, overlaid with three translucent reciprocal space spherical intensity maps closely spaced around the peak position. **aii**, I(q) plots for the SAXS-TT reconstruction using the s = 0 harmonic (circles), 2D averages (square) and a narrow profile along the vertical (dash-dot), which gives the true value and can be estimated from the TomoSAXS fit. Blue: Gaussian fit to the SAXS-TT reconstruction. Dashed vertical line: true q_0_ value; dotted blue vertical line: estimated from Gaussian fit. **aiii**, % error in q_0_ and w_a_ when estimated using the Gaussian fit, for varying values of w_μ_ (inversely related to fibril radius), showing progressive deviation at higher w_μ_. **bi**, 3D rendering of the reciprocal space intensity and spherical intensity maps, for 3 fibres separated angularly by 20° in the same plane and in the same voxel. **bii**, SAXS-TT reconstruction of the combined scattering (circles/dashed line) and Gaussian fit to SAXS-TT profile (red line). Green, orange and blue lines denote narrow angular profiles along the centre of each fibre orientation, as estimated with TomoSAXS, giving the true q_0_ values (vertical dotted lines, same colour code). **biii**, Comparison of TomoSAXS-reconstructed data (dotted line) versus values calculated by SAXS-TT for a range of fibril parameters; since the SAXS-TT I(q) calculates an average scattering characteristic of fibres in the voxel, there is less sensitivity to inter-fibre variation.

TomoSAXS thus presents a powerful addition to multimodal X-ray imaging for spatial surveys of nanoscale properties^53^, offering key advantages over approaches such as SAXS-TT^28^ for the study of fibrous materials (Supplementary Note 20). By spatially registering SAXS data to collagen fibres defined by their 3D orientations (from pCT), TomoSAXS enables fibre-specific—rather than voxel-specific^28^—assessment of nanostructure. This allows direct analysis of heterogeneous fibre-scattering profiles, with resolution determined by pCT (here 1.6-µm voxel size) rather than the SAXS beam size (here 20 µm; Extended Data Fig. 10), and estimation of fibre-specific nanoscale strain. In addition, TomoSAXS only requires approximately 1% of the theoretical minimum number of SAXS angular projections needed for tomographic reconstruction (Supplementary Note 23).

The combined CT-DVC-SAXS approach is essential to resolve fibril-level strains, with the DVC also providing strain information at the fibre and tissue scales. Tracking fibres across slices allows interpolation methods that preserve fibre continuity. Using the structural model of the fibril-scattering function, rather than the more general spherical harmonic approach, allows more accurate measurements, albeit currently at the cost of increased computational time and greater specificity of the scattering functions for the material investigated. Here the relatively large angular step (22.5°) used for rotations in the rat IVD case (Methods) limited the number of fibres detectable under Ewald conditions to approximately 70%, which can be improved in the future by higher angular resolution (5−10°; Supplementary Fig. 5a). In each horizontal slice around 35–85% of the detectable fibres were solved, which were distributed reasonably uniformly (but not densely) across the tissue volume (Supplementary Fig. 6). To improve spatial resolution, future work may look to improve the SAXS reconstruction pipeline by adding higher-order overlaps to increase the fraction of solvable fibres or adapting alternate global solve strategies.

The reconstruction of fibril properties within an intact human IVD has demonstrated the scalability of the TomoSAXS method; however, challenges arise from the order of magnitude increase in size compared with small-animal models, with 1.5 TB raw HiP-CT data and 800 GB raw SAXS data generated per sample, compared with 42 GB pCT data and approximately 100 GB SAXS data for the small-animal samples tested here. Formalin fixation of the sample will have impacted the nanoscale collagen properties measured here, and future investigation into the effects of fixation should be performed.

Scan time remains a limitation, particularly for the investigation of mechanics in viscoelastic samples, as samples must be stable during scanning (Supplementary Fig. 4). Here the total experimental time for an in situ experiment was approximately 4 h (Extended Data Fig. 1). The need for stress relaxation means that the dynamic response of materials immediately following loading is not captured. Fast (5 s) pCT scan times have been used to capture dynamic loading of cartilage^54^, and could be combined with the DVC to determine tissue-level and fibre-level strains during loading. Radiation dose is also critical, and its effect on IVD mechanics requires further testing. The total radiation dose applied during in situ experiments was 80.6 kGy (12.2 kGy per SAXS and 26.6 kGy per pCT), compared with approximately 2,000 kGy that would be required to perform SAXS-TT measurements using the same setup. This dose level may have led to a stiffening of collagen fibrils^55^, but is unlikely to have had a major effect on organ-level or tissue-level mechanics, based on results from previous studies at similar dose levels^22,54,56^. Total dose for TomoSAXS of human IVD was 15.5 kGy (3.8 kGy SAXS and 11.7 kGy HiP-CT). Although efforts were made to perform physiologically relevant in situ IVD mechanical experiments, truly physiological conditions would require the use of a humidity and temperature-controlled sample environment and multi-axis mechanical testing rig compatible with synchrotron beamlines.

Looking ahead, the TomoSAXS methodology is broadly applicable to other collagenous and soft biological materials, such as tendon^3,25^ and cornea^57^, as well as engineered materials. Indeed, experiments on man-made composites would probably have far fewer limitations in terms of viscoelastic effects, hydration and radiation dose. We have demonstrated the feasibility of performing TomoSAXS on intact human IVD, with the potential to extend the technique to other organs including the heart and lungs. Although synchrotron radiation was used here, TomoSAXS could also be undertaken with laboratory X-ray sources at the expense of markedly increased acquisition times. The measurement of fibre orientations from pCT could be replaced with other 3D microscale-imaging techniques, such as optical coherence tomography^58^. Extending to engineered materials, our 3D diffraction modelling software may also be developed for other periodic and angularly dependent materials including nanostructured polymers^7,59^ and carbon fibre composites^8^.

## Methods

### Sample acquisition and preparation

Samples originated from male 8-week-old Sprague Dawley rats kept in controlled conditions (12–12-h light–dark cycles, 22 °C and ad libitum access to food and water) at the University of Manchester Biomedical Sciences Facility. All tissue collection procedures were performed in accordance with the UK Animals (Scientific Procedures) Act 1986, local regulations set by the UK Home Office with institutional approval obtained from the University of Manchester Animal Welfare and Ethics Review Board under the Home Office Licence (#70/8858 or I045CA465). Whole spines were dissected and snap frozen in liquid nitrogen before storage at −80 °C. Thawing and further dissection were performed within 24 h of experimentation, as described by Parmenter et al.^22^. This resulted in whole IVD samples (from vertebral levels thoracic 10 to lumbar 2; Supplementary Table 1) with half vertebrae on either side. No randomization or blinding was carried out.

Samples were mounted in resin-printed holders (Supplementary Fig. 1) using cyanoacrylate glue (VetBond; 3 M Science), with sample alignment designed so that the mid-transverse slice of the IVD was parallel to the loading plates. A Kapton polyimide tube enclosed each specimen, leaving approximately 0.5 mm exposed. Holders were placed in a Deben CT500 micromechanical tester (100 N loadcell, ±1% accuracy; Deben) with a custom open-frame design^22^ (Supplementary Fig. 1). Displacement was recorded using a 10-mm extensometer (3-μm resolution, 1% linearity). The open-frame configuration facilitated alignment and hydration of samples while minimizing beam-path obstruction during SAXS. Sample hydration was maintained through immersion in phosphate-buffered saline (PBS), supplied via a capillary at the holder base from a 100-ml syringe through a stopped-flow module (Bio-Logic). A continuous flow preserved fluid level despite minor leakage from the 3D-printed holder.

### Overview of the SAXS–pCT experiment

Although simultaneous SAXS and pCT would have been ideal, in the absence of a dedicated SAXS–pCT beamline at Diamond Light Source (DLS), a sequential approach was taken (SAXS(I22) preloaded → pCT(I13) preloaded → pCT(I13) loaded → SAXS(I22) loaded), as illustrated in Extended Data Fig. 1, with special stabilized sample holders and identical mechanical rigs at both beamlines to ensure comparability (Supplementary Fig. 1).

### Tomographic SAXS acquisition

SAXS data were acquired at the I22 beamline of the DLS synchrotron (experiment SM29784-8, 12–15 December 2023). A Deben CT500 was mounted to a rotation stage, on top of the sample table. A Dectris Pilatus P3-2M detector was placed at a distance of 9 m from the stage, with a vacuum tube in between to minimize air scatter. All SAXS measurements used a beam energy of 14 keV and beam size of 20 µm. Each sample was first mapped at 0° angular orientation using a raster map of approximately 100 × 100 SAXS frames using a 50-ms exposure time and a 20-µm step size. Maps were inspected to identify the IVD, and a 5 × 1 mm region of interest was assigned within the IVD for tomographic SAXS scans. SAXS scans were undertaken over raster maps of approximately 250 × 10 SAXS frames with a step size of 20 µm using 150-ms exposure time for nine equally spaced angular orientations (22.5° step) between −85° and 95° (to minimize blocking by mechanical testing support rods). Radiation dose for a full single axis SAXS tomography was 12.2 kGy (Supplementary Note 1). A relatively large step size of 22.5° was used due to a significant reduction in flux caused by the reduced ring current of 200 mA during the beamtime. The experiment can be readily scaled to a smaller angular step (greater fraction of fibres intersecting the Ewald sphere). SAXS data for one horizontal slice is available in the Supplementary Dataset^59^.

### pCT acquisition and reconstruction

pCT was conducted at the Diamond-Manchester Imaging Branchline I13-2, DLS (experiment SM29784-9, 12–14 December 2023). The setup followed established protocols^22^ (mean energy of 27.6 keV, 1,800 projections, 0.5-m sample–detector distance), with only the exposure time adjusted to 0.15 s to account for the reduced ring current mentioned in the previous subsection. The insertion device gap was set to 5 mm during scanning and widened to 7 mm for alignment and low-resolution scans to reduce dose. Initial scans used a 1.25× objective (voxel size of 2.6 µm and field of view of 6.7 × 5.6 mm) to capture the sample environment, including the Kapton tube for SAXS co-registration. Subsequent scans used a 2× objective (voxel size of 1.625 µm and field of view of 4.2 × 3.5 mm) before and after compression. Radiation doses were 1.35 kGy (voxel size of 2.6 µm) and 27 kGy (voxel size of 1.625 µm) per scan (Supplementary Note 2). Tomograms were reconstructed using the open source Python library Savu 4.2 (https://savu.readthedocs.io) and further processed using ImageJ 1.54 as previously described^22^. Images of IVD1 are available in the Supplementary Dataset^60^.

### In situ mechanical testing

Samples were held under axial compression, applied in the laboratory vertical *z*-direction, during scanning. For a reproducible mechanical protocol, two identical Deben CT500 mechanical testing rigs with 100 N load cells were used during the experiment, one placed at beamline I22 (provided by Imperial College London) and the other placed at beamline I13-2 (provided by the University of Manchester at Harwell). A preload of 1 N, corresponding to approximately 40% the animals body weight (260 ± 33 g), was applied at I22 before SAXS scanning to prevent over swelling of the nucleus pulposus (Extended Data Fig. 1b). Samples were allowed 20 min of stress relaxation before scanning to minimize motion, determined as the time taken for load readings to stabilize (less than 0.01-N change in 1 min). Following SAXS, samples were clamped in position using bespoke 3D-printed sample holders and screws (Supplementary Fig. 1) and transferred to I13-2 for pCT (Extended Data Fig. 1b).

Clamped samples were placed in the mechanical testing rig at I13-2 and secured at the top (Extended Data Fig. 1c). The bottom platen of the rig was slowly raised until screw hole alignment was reached between the rig and the clamped sample holder, which was then secured in place. The clamps were removed from the sample holder before preload pCT scans. After the first pCT scan at 1.6-µm voxel size, a 50-µm displacement was applied to the bottom platen of the sample holder in the laboratory *z*-direction, leading to a 2–5% reduction in IVD height (Supplementary Table 1). Axial compression was chosen for ease of experimentation and interpretation of results. Again, samples were given 20 min or more of stress relaxation before scanning. After imaging, the sample was clamped in its loaded state before being removed from the rig and transported back to I22 (Extended Data Fig. 1c). At I22, the sample was placed back in the mechanical testing rig and clamps were removed before taking the loaded SAXS scans, which used the same protocol as described in the section ‘Tomographic SAXS acquisition’ (Extended Data Fig. 1d).

### Fibre tracing and DVC analysis

Fibre tracing of the preload 1.6-µm images was performed using the Avizo XFiber module (Avizo 3D 2023.2; Supplementary Fig. 2), with parameters used for tracing given in Supplementary Note 3. After fibre tracing, fibres clearly corresponding to imaging artefacts—such as streaks from mineralized tissues and ring artefacts from reconstruction—were removed from further analysis. Fibre spatial graph data were exported as a .xml file, and fibre identification number was conserved throughout all further analysis.

Fibre orientation and curvature were computed per fibre from a 3D polynomial space-curve fit to the traced fibre centreline^4^ (*r*(*s*)) using a custom-written MATLAB script (MATLAB R2023a, https://github.com/DiamondLightSource/tomosaxs; Supplementary Note 4). This resulted in fibre ID, *x*, *y*, *z*, curvature, *α*-orientation and *β*-orientation values for points placed every 6.5 µm along each fibre. Fibre ID, *α*-values and *β*-values were converted to 3D volume images for input into the TomoSAXS reconstruction (Supplementary Note 5).

Fibre curvature (*k*(*s*)) was evaluated from the first and second derivatives of the fitted curve^4^ using the standard 3D definition:

$$k(s)=\frac{\Vert {{\bf{r}}}^{{\prime} }(s)\times {{\bf{r}}}^{{\prime\prime} }(s)\Vert }{{\Vert {{\bf{r}}}^{{\prime} }(s)\Vert }^{3}}$$

A local DVC analysis with flexible point cloud specification was performed using the open-source software iDVC (https://tomographicimaging.github.io/iDVC/)^4,21–24,61^. The fibre-based coordinates generated above were used as the DVC point cloud defining the locations of sub-volumes used for tracking (Fig. 2e), resulting in 0.7–1.4 million DVC measurement points per sub-volumes for each sample (Supplementary Table 1). Spherical sub-volumes with a diameter of 40 voxels (65 µm) were matched between the preload and loaded images using a zero-normalized sum-of-square difference function for optimization and 12 degrees of freedom (translation, rotation and first-order strain; Fig. 2g). Six thousand sampling points were placed within each sub-volume for matching. The displacement of the first sub-volume in the point cloud was defined by manually tracking a landmark between the preload and loaded images using ImageJ.

Two strain measurement techniques were applied to the displacement fields measured from the DVC, corresponding to different length-scales probed. For tissue-level strains, the differential of a polynomial fit to the displacement field was used to calculate the Lagrangian strain tensor at each point^61^. The 75 closest points to the measurement point were used for fitting to smooth out noise in the displacement field and improve accuracy. The first principal strain was derived from the maximum positive eigenvalue, and the third principal strain was derived from the maximum negative eigenvalue of the strain tensor. The direction of the corresponding eigenvectors relative to the local fibre direction was calculated as an angle between 0° (parallel to fibre direction) and 90° (orthogonal to fibre direction; Extended Data Fig. 5g). Radial and circumferential strains were calculated by projecting the strain tensor along the local radial and circumferential directions (https://github.com/DiamondLightSource/tomosaxs; Extended Data Fig. 5a and Supplementary Note 6). Fibre strains were calculated using a custom-written MATLAB script (MATLAB 2023a), which performs a polynomial fit to displacement along the fibre direction (https://github.com/DiamondLightSource/tomosaxs; Supplementary Fig. 3 and Supplementary Note 7).

The double projection zero strain method^22^ was used to quantify the accuracy of DVC displacements and strains (Supplementary Note 9). Zero-strain displacement accuracy (mean absolute error) was 88.5 nm and precision (standard deviation of absolute error) was 83.5 nm. Tissue zero-strain accuracy was 0.16% and precision was 0.16%. Fibre zero-strain accuracy was 0.08% and precision was 0.10%. The DVC was able to track a virtual deformation of 10% compression with a displacement accuracy of 198 nm, tissue strain accuracy of 0.40% and fibre strain accuracy of 0.45%.

To calculate orientation for the same fibres in each sample after loading, DVC displacements were applied to the fibre points in MATLAB (https://github.com/DiamondLightSource/tomosaxs; Supplementary Note 10). Orientation of the deformed fibres was calculated, and loaded Fibre ID, *α* and *β* 3D volume images generated for input into the TomoSAXS reconstruction.

### 3D diffraction modelling

3D diffraction modelling was applied by simulating the meridional X-ray diffraction peaks of collagen fibrils with nanoscale fibre symmetry, under user-defined experimental conditions (wavelength, lattice spacings and angular tilts), and accounting for the variable intersection of the 3D scattering intensity with the Ewald sphere. Implementation was done in a custom Python library developed by us (https://github.com/DiamondLightSource/tomosaxs) and is a more complex version of the model presented by Badar et al.^1^. In brief, the meridional scattering arising from the D-periodicity is simulated by a set of 3D semi-flat ellipsoidal functions, whose location in 3D space is at multiple harmonics of the inverse D-period (*q*_0*n*_ = 2π*n*/*D*), radial width in *q* (*w*_a_) is related to fibril pre-strain heterogeneity, and angular width (*w*_μ_) is related to inverse fibril diameter and intrafibrillar tilting, parameterized by a degree of circularity of the ellipsoid function (*δ*; 0 = straight function; 1 = circular ellipsoid). The need for a semi-flat, rather than completely flat, meridional ellipsoid came from the experimental observation that even for tissues with uniaxial fibril orientation, the meridional ellipse showed some arcing (Supplementary Note 11). We believe this may be related to the intrafibrillar tilt of the microfibrils^38^.

Owing to the intersection with the Ewald sphere (radius ∝ 1/*λ*), the intensity and pattern of the resulting 2D fibril diffraction depends on user-input 3D tilt angles *α* and *β* of the fibril, which will be a crucial factor in deciding which fibres contribute to scattering, as well as the beam wavelength. The functions for fibril scattering can be calculated at any point across the diffraction condition of the sphere both in 2D (Fig. 2b, left panel), as well as in 1D (for example, azimuthal (*χ*) and radial (*q*) plots; Fig. 2c) by averaging in the complementary axis.

### TomoSAXS algorithm overview

Here the foregoing library (https://github.com/DiamondLightSource/tomosaxs), in conjunction with the pCT or DVC data (for *α* and *β*), was used to reconstruct the third-order collagen diffraction peak (which typically has intensity between wavevectors of approximately 0.26–0.32 nm^−1^). The TomoSAXS implementation works via a series of discrete modules in the Python (3.10) environment, summarized here.

- Module 1 spatially co-registers the pCT and SAXS scans, from which the individual collagen fibres in each SAXS beam path at all tomographic rotations can be found.
- Module 2 performs background correction of the SAXS signals, accounting for PBS, Kapton tube and air scattering, building on the adcorr Python package^62^. The pCT and co-registered SAXS scans from module 1 allows accurate correction using the variable thickness of the sample through each SAXS beam path by adjusting the proportion of dispersant to sample, compared with assuming a uniform thickness in Pauw et al.^62^.
- Module 3 provides initial estimates for per-fibre SAXS amplitude, using singular-value decomposition (SVD) and the 3D diffraction model.
- Module 4—the core of the reconstruction—extracts the nanoscale parameters in an iterative way, by scanning through the SAXS tomography along *χ* and rotation axes to find angular regions where either a single fibre, a pair of fibres or triplet of fibres dominates the observed scattering over a given angular sector (10° in width). Fibres are isolated at the single, double or triplet level when one, two or a maximum of three fibres contribute predominantly (more than 90%) to total scattering across at least a 10° sector around the circumferential *χ* axis of individual SAXS frames (Fig. 2c, top panel). By calculating SAXS radial intensity profiles within this azimuthal sector at three equally spaced angular positions (for example, at 0–3.33°, 3.33–6.66° and 6.66–9.99° from the start of a 10° sector), and fitting each radial profile with 3D diffraction models, the parameters mean D-period (inverse to radial peak centre; equation (1)); variation in D-period (peak width along *q*); and fibril radius (inversely related to peak width along *χ*) can be estimated. For double-fibre and triple-fibre fits, to avoid degenerate cases where multiple fibres are pointing in the same direction and with similar fibril parameters, we enforced a minimum separation of 5° between fibrils, as well as minimum predicted intensity levels from each fibre to avoid dominance by one (for doublet) or two (for triplet) fibres.
  To iteratively solve for the fibre parameters, a two-stage process was followed (Supplementary Fig. 7). First, a sweep was conducted across all rotations and beam paths to isolate and fit single fibres with sufficient independence in scattering intensity across *χ*, until no more individually isolatable fibres were solvable. The process was then repeated to include fitting for single, double and triple sets of fibres across all beam paths and rotations, looping until no further fibres were solved in a single sweep through all angles and rotations.
- Module 5 spatially reconstructs and outputs the estimated metrics and estimates fibril-level (nanoscale) strain in biomechanical loading, using DVC estimates of per-fibre (microscale) deformation to locate the same fibre in the unloaded and loaded datasets.

### Module 1: spatial co-registration

Spatial co-registration of SAXS and pCT datasets was achieved through identifying fiducial markers (such as the mineralized vertebral endplates) in the samples, which gave distinct signals in SAXS and pCT. The Kapton tube of the sample environment was used for horizontal alignment and the mineralized tissues of the vertebral endplates used for vertical alignment. High-resolution (1.625 µm) pCT images did not capture enough Kapton tube for co-registration, so were spatially registered to low-resolution (2.6 µm) images. Both sets of images were downsampled to a voxel size of 6.5 µm and the offset between them calculated in *x*, *y* and *z* coordinates. Fibre orientation tiff stacks (absolute voxel size of 8.125 µm) were padded to the size of the original high-resolution (1.625 µm) reconstruction (512 × 512 × 432 voxels) and centred using the offset between high-resolution and low-resolution images, so that all pCT data were spatially aligned (Supplementary Note 12).

Vertical registration between SAXS and pCT involved identifying the border of the mineralized vertebral endplates in the SAXS intensity map generated during coarse mapping at 0°, and in the 2D total phase-contrast intensity maps of a sample along the same angular orientation from the pCT data and calculating the vertical offset between them. Horizontal registration was achieved by identifying the Kapton tube from its characteristic peaks in SAXS intensity, and manual segmentation of the tube from the pCT data. For each SAXS tomography orientation (*n* = 9, −90° to 90°), the fibre orientation, index and Kapton data for vertically isolated SAXS slices were rotated to the corresponding angle (using a custom function for maintaining fibre data integrity during rotation; Supplementary Note 13). The location of the left-hand edge of the Kapton tube was then compared and aligned between the SAXS and pCT data. This allowed finding the indexed fibres that contributed to SAXS scattering along each beam path of the raster scan in the specific SAXS slice. For each frame, the orientation, index and number of CT voxels comprised by each indexed fibre along its beam path were saved, along with the calculated distance between each fibre and the centre of the beam path, which was used as a weighting factor for model fitting. This data were saved as individual matrices, along with the calculated total specimen thickness (along beam path) for each frame.

### Module 2: background correction

Background correction was undertaken for each individual SAXS frame following the method outlined in Pauw et al.^62^. A separate scan using the same parameters as the SAXS tomography was undertaken for a single slice (a slice represents a 1D scan × *n* = 9 rotations) through (1) an empty Kapton tube, and (2) a Kapton tube filled with PBS, to represent background SAXS signals from both the sample holder (Kapton tube) and the dispersant (PBS). These signals were algorithmically subtracted from each sample SAXS frame using the adcorr Python library (https://github.com/DiamondLightSource/adcorr), adjusting the ratio between the sample and dispersant based on the sample thickness for the respective frame (module 1). The resultant background-corrected data were saved as .h5 files for each TomoSAXS slice.

### Module 3: SAXS amplitude estimation

The first analytical step in the TomoSAXS algorithm makes an initial estimate of the amplitude of SAXS scattering for individual fibres using the SVD, using a mean value for the other peak characteristics such as position, radial and angular widths (Supplementary Table 2 and Supplementary Note 14). This was justified by the small variability of the peak position (from 2D data) across different sample locations. The estimation step was used to identify locations of strong scattering for specific fibres enabling fibre-by-fibre solution in the next module 4 step.

At a given beam path in the sample *x*, and a rotation angle *r*, the angular intensity of the third-order collagen peak *q*_0_ can be written as

$${I}_{x}^{r}\,(\chi )=\mathop{\sum }\limits_{i}^{F}{w}_{i}{I}_{i}^{r}(\chi ;{q}_{0},\Delta {q}_{0},\alpha ,\beta )$$

where the sum in *i* is taken over all fibres in the volume, with weighting factors either 0 (if outside of the beam path) or proportional to the intensity at the fibre location (assuming a Gaussian beam profile with known shape). As all fibres are taken to have the same average peak location and width parameters in this initial estimation step, the contribution from each fibre ${I}_{i}^{r}(\chi ;{q}_{0},\Delta {q}_{0},\alpha ,\beta )$ can be calculated up to a scaling factor proportional to the amplitude factor *A*_*i*_, including the geometrical transformations needed to account for fibre orientation with respect to the beam. Hence, when represented for each x,χ,r the above equation can be expanded into an overdetermined set of linear equations, which can be solved by SVD.

A Python dictionary was created with entries for each SAXS frame comprising of 0–180° intensity(*χ*) (herein *I*(*χ*)) radial integrations between 0.26 and 0.32 nm^−1^ in *q*, binned to 36 averaged points across *χ* and smoothed using 1D Savitzky–Golay filtering (window length of 9 and polynomial order of 1). Two matrices were then created: the first consisting of a single vector comprising the concatenated *I*(*χ*) integrations for all SAXS frames (left-hand side (LHS) in the equation above); and the second with columns comprising the contribution of each fibre to the same binned *I*(*χ*) intervals predicted for each SAXS frame using 3D diffraction modelling (right-hand side (RHS) in the equation above). The amplitude for each fibre was then estimated by non-negative linear least squares regression, regressing predicted versus measured *I*(*χ*) data. A Python dictionary was constructed, with entries for each indexed fibre consisting of its index, *α* and *β* angles and an empty entry for fitted nanoscale parameters. This dictionary was used to calibrate registration matrices (see above) for each rotation angle of the SAXS tomography, creating a series of other dictionaries.

### Module 4: TomoSAXS fitting

#### Initial intensity simulation

As shown in Fig. 2f–h, TomoSAXS reconstruction was based on fitting fibre diffraction models of intensity to measure *I*(*q*) profiles across the azimuthal angular ranges (Fig. 2g) of independent scattering signals from either (1) a single fibre, (2) a pair, or (3) a triplet of overlapping fibres (Supplementary Table 2). We first calculate simulated angular SAXS intensity profiles using estimated amplitudes from module 4. A Python fibre dictionary was constructed, containing its index, *α* and *β* angles, initial estimated scattering amplitude and a dictionary for fitted nanoscale parameters. The dictionary was referenced against registration matrices (see above, module 1) for each rotation angle of the SAXS tomography, creating a series of individual orientation-specific dictionaries, indexed by beam path.

For each beam path in the orientation-specific dictionary, a dictionary indexed by fibre index stored information for every fibre in the beam path (including initial estimated amplitude), with a weighting term from the calculated distance of the fibre to the beam centre (Supplementary Note 13) where *d* is the distance from the beam centre and *w* the beam diameter:

$${\mathrm{weighting\; factor}=e}^{{-d}^{2}/2{w}^{2}}$$

These dictionaries were used to create simulations of the angular scattering intensity of the third-order meridional peak of every beam path using 3D diffraction modelling, using tilt angles obtained from pCT and the averaged nanoscale peak metrics (*q*_0_ = 0.2856 nm^−1^, *w*_a_ = 0.005 nm^−1^, *w*_µ_ = 0.2 nm^−1^ and *δ* = 0.5; see Supplementary Note 14 for rationale).

#### Fitting single, double and triplets of fibres

The angular intensity profile was scanned to iteratively solve individual or small groups of fibres, rather than fitting all fibres to the data, simultaneously (Supplementary Note 16).

*Single-fibre fit.* For each fibre *k* in a beam path *p* at a rotation *r*, its individual angular intensity contribution ${I}_{k}^{p}(\chi ;{r})$ to the total intensity ${I}^{p}(\chi ;{r})$ was calculated. If the following condition was fulfilled for an angular range $[{\chi }_{k;1}^{p},{\chi }_{k;1}^{p}+\Delta \chi ]$ between 0 and 2π (Δ*χ* = 10°), and a single-fibre dominance threshold *t*_*s*_ = 0.90:

$${I}_{k}^{p}(\chi ;r) > {t}_{s}{I}^{p}(\chi ;r);{\chi }_{k;1}^{p} < \chi  < {\chi }_{k;1}^{p}+\Delta \chi $$

then the fibre *k* was identified as being the dominant contribution to the SAXS signal in the $[{\chi }_{k;1}^{p},{\chi }_{k;1}^{p}+\Delta \chi ]$ angular range. The index, beam-path location and angularly independent sector $[{\chi }_{k;1}^{p},{\chi }_{k;1}^{p}+\Delta \chi ]$ was recorded for all fibres that met the user-defined criteria for independent scattering were stored in a Python list.

Next, these fibres were fitted to extract the nanoscale (fibrillar) peak metrics, by calculating ${n}_{\chi ,c}$ (default = 3) equally spaced radial slices (azimuthally integrated) across the $[{\chi }_{k;1}^{p},{\chi }_{k;1}^{p}+\Delta ]$ angular region, with a width $\Delta {\chi }_{c}$ (default = 6°). These were simultaneously fitted with 3D diffraction models for the fibre, using the minimize() function of the lmfit Python library (https://lmfit.github.io/lmfit-py/fitting.html) and the Nelder–Mead algorithm. The angles *α* and *β* and the weighting factor of the fibre were fixed parameters, and *q*_0_, *w*_a_, *w*_μ_, amplitude and *δ* were set as fit parameters (see Supplementary Table 2 for settings and Supplementary Note 15 for rationale). If the fits passed several quality thresholds, both relating to absolute values of the parameters and statistical quality of fit (for standard error, threshold = 25%), the fit values were added to the dictionary entry for the respective fibre. Instances in which the fit to the fibre did not meet quality thresholds were saved and added to a separate Python list allowing them to be discarded from future investigation. This process was repeated for all the fibres in the list.

As successful fits allowed more potential single-fibre instances to be identified (already fitted fibres could be deconvolved from the scattering in all beam paths where they occur), the process was repeated until no more instances of independent scatter for single fibres could be found for any beam paths in the SAXS tomography.

The above process isolated and fitted the instances with the strongest independent scattering signal (approximately 10–20% of indexed fibres per slice), providing robust fits for further deconvolution and fitting of other fibres occurring in the next step of the reconstruction algorithm.

*Overlapping fibre fit.* In the next step, the reconstruction algorithm included fitting of instances where two to three fibres produced a combined scattering that meets the thresholds for independent scatter. For example, for all pairs of fibres ${k}_{1},{k}_{2}$ in the beam path p, we checked whether the following condition was fulfilled for any $[{\chi }_{k;1}^{p},{\chi }_{k;1}^{p}+\Delta \chi ]$ and a double-fibre dominance threshold *t*_*d*_ = 0.95:

$$({I}_{{k}_{1}}^{p}+{I}_{{k}_{2}}^{p})(\chi ;r) > {t}_{d}{I}^{p}(\chi ;r);{\chi }_{k;1}^{p} < \chi  < {\chi }_{k;1}^{p}+\Delta \chi $$

For multiple-fibre fits, validation simulations (Supplementary Note 15) have shown that the dominance of one fibre’s scattering intensity can bias the fit of the other fibre (or fibres) and lead to inaccuracies in fits. Hence, upon identification of a pair of fibres ${k}_{1},{k}_{2}$, the relative combination of scatter from each fibre was estimated. Only instances in which each fibre represented a minimum of ${t}_{d}^{1},{t}_{d}^{2}$ = 20% of the total scatter across > 90% (0.90) of the angular sector $[{\chi }_{k;1}^{p},{\chi }_{k;1}^{p}+\Delta \chi ]$ were considered for fitting. Instances that passed this test were then fitted using the minimize() function as above.

After each fit, the parameter database was updated, and each angular intensity profile was scanned to identify any new solvable fibres (single, double or triple), which could be fitted by deconvolving already fitted fibres.

The foregoing steps were looped through all beam paths at all rotations, for a user-defined maximum number *n*_*l*_ = 10 times, recording the ratio of fitted versus total indexed fibres at the end of each loop. If no increase in fitted fibres was observed at the end of a loop, the loop was terminated as all possible fibres had been fitted.

The entire TomoSAXS pipeline was conducted on a per-slice basis (per-horizontal 20-µm SAXS tomography slice) using cluster computing (specifically the DLS computing cluster). This allowed each slice to be run in parallel to maximize efficiency. The complete process across each of the five modules was completed within 6–7 days, with each module triggered individually in an iterative manner. TomoSAXS fitting (4–5 days) resulted in a mean proportion of fitted fibres of 60.08% per slice. This included multiple fits for differing instances of individual fibres found within different TomoSAXS slices. It is important to note that this does not correspond to the proportion of the total sample represented in each slice being fitted, as TomoSAXS operates on a per-fibre basis as opposed to a per-voxel basis (as in SAXS-TT^28^). Reconstructions are spatially heterogeneous and dependent on the density and orientation of fibres within the respective slice.

### Module 5: TomoSAXS reconstruction

Once fitting was complete, fitted parameters were spatially reconstructed for scanned fibres to create volumetric maps of per-fibre values. This was performed per slice for each parameter, by creating blank maps of the same shape as fibre index maps (created during the registration process; Supplementary Note 13) and assigning metric values to the voxels assigned to the index of every solved fibre in index maps. For fibres with fitted values in multiple TomoSAXS slices, if slices remained unfitted between these points, they were provided with values between fitted values estimated using linear interpolation.

#### Per-fibre nanoscale strain reconstruction

To estimate strain in nanoscale parameters under compressive load, indexed fibres were isolated that provided fits in both unloaded and loaded data. For each unloaded fit, the estimated vertical deformation of this point (and thus position in the loaded TomoSAXS scan) was estimated using the vertical (*z* axis) deformation measurements provided by DVC analysis (Supplementary Note 17). Strain in each parameter was calculated as the percentage difference between unloaded and loaded TomoSAXS fits for each point.

### Validation using digital phantoms

The spatial calibration between pCT and SAXS data allowed estimation of the accuracy of fits supplied by the TomoSAXS technique, by providing known artificial nanoscale parameters to each indexed fibre in each TomoSAXS slice (maintaining fibre orientation, initial estimated scattering amplitude and per-instance weighting; Supplementary Note 18). Experimental noise was added to the simulated SAXS data, and accuracy and precision of nanoscale parameter estimates calculated as mean absolute error and standard deviation of absolute error.

### Laboratory TomoSAXS analysis

Chicken foot tendons were dissected and air cured under differing tare loads of 0.5 N, 1.5 N and 3.5 N to generate different levels of pre-strain in the collagen. Three tendons were arranged at different overlapping orientations and superglued to a 3D-printed sample holder (Extended Data Fig. 2a). SAXS scans were performed using a Xenocs Xeuss 3.0 (DLS) with a beam size of 400 µm and exposure time of 5 min. Nine angular rotations between 0° and 180° were used, with 11 beam paths per rotation. µCT of the sample was performed using a Phoenix V|tome|x (Research Complex at Harwell) with a voxel size of 14.3 µm.

The orientation of collagen fibrils within each tendon was assumed to be aligned with the principal axis of the tendon. The sample was divided into an 11 × 11 grid for TomoSAXS reconstruction rather than individual fibres, for the purpose of a simplified, proof-of-concept reconstruction (Extended Data Fig. 2). TomoSAXS reconstruction followed modules 4 and 5 of our pipeline, with no background correction or SVD analysis of scattering amplitude applied.

### TomoSAXS of human IVD

Static imaging of a whole, formalin-fixed, human IVD (spinal level thoracic 11–12, 64-year-old male donor, no known spinal pathologies) was performed at the BM18 beamline of the ESRF using hierarchical pCT^45^ (Supplementary Note 21). Donation and dissection were performed at the Laboratoire d’Anatomie des Alpes Françaises in November 2023, in accordance with French legislation governing body donation. Subsequent transport and imaging protocols were approved by the UK Health Research Authority through the Integrated Research Application System (#200429) and the French Ministry of Health.

The entire IVD was captured with a voxel size of 10 µm and a local zoom with a voxel size of 4 µm performed centrally to enable clear visualization of collagen fibres throughout the complete organ (Fig. 4b,d). Fibre tracing was performed on the 10-µm scan using the Avizo XFiber module (Avizo 3D 2023.2), with parameters used for tracing given in Supplementary Note 21. Correlative high-energy (75 keV, beam size of 100 µm) tomographic SAXS was performed at ESRF beamline ID31 at 24 angles (Supplementary Note 22).

TomoSAXS reconstruction of a single slice of human IVD followed a modified pipeline to overcome the increased number of angular rotations, the 10^3^ increase in sample volume and concomitant increase in fibre density per beam path. In principal, this involved three primary modifications. First, *α* and *β* fibre-angle maps were independently quantized by *k*-means clustering of non-zero angles rounded to 3° increments, then combined pixel-wise into a 3D orientation label map of unique vertical–horizontal fibre pairs, reducing fibre indices from more than 26,000 to approximately 3,600. Second, we extended the multiple-fibre fitting strategy to include four-fibre and five-fibre clusters across the angular *χ* axis. Finally, to handle the larger number of beam paths and greater amount of indexed objects per beam path, we introduced parallel processing. Instead of running rotations sequentially, each rotation was processed in parallel on a computing cluster, with all rotations reading from and updating a shared database of fitted fibres so that they could inform one another during execution. For more details of these modifications, see Supplementary Note 22.

### Statistical analysis

Two-sided ordinary least squares regression analysis was used to test the agreement of known versus estimated properties of digital phantom data with and without the addition of experimental noise (Extended Data Table 1). Correlations between per-fibre mean values of fibre strain, D-period, D-period variation and D-period strain were tested using ordinary least squares regression (Extended Data Table 2). No adjustments were made for multiple comparisons.

## Online content

Any methods, additional references, Nature Portfolio reporting summaries, source data, extended data, supplementary information, acknowledgements, peer review information; details of author contributions and competing interests; and statements of data and code availability are available at https://doi.org/10.1038/s41586-026-11148-5.

## Supplementary information

Supplementary Information Supplementary Tables 1 and 2, Supplementary Figs. 1–8 and Supplementary Notes 1–23 providing detailed experimental methods, reconstruction parameters, validation, image processing, DVC and fibre-strain analyses, TomoSAXS reconstruction workflow, digital phantom validation, comparisons with existing SAXS methods, human intervertebral disc implementation, and supplementary references.

Peer Review File

## Figures and Tables

**Extended Data Table 1:**
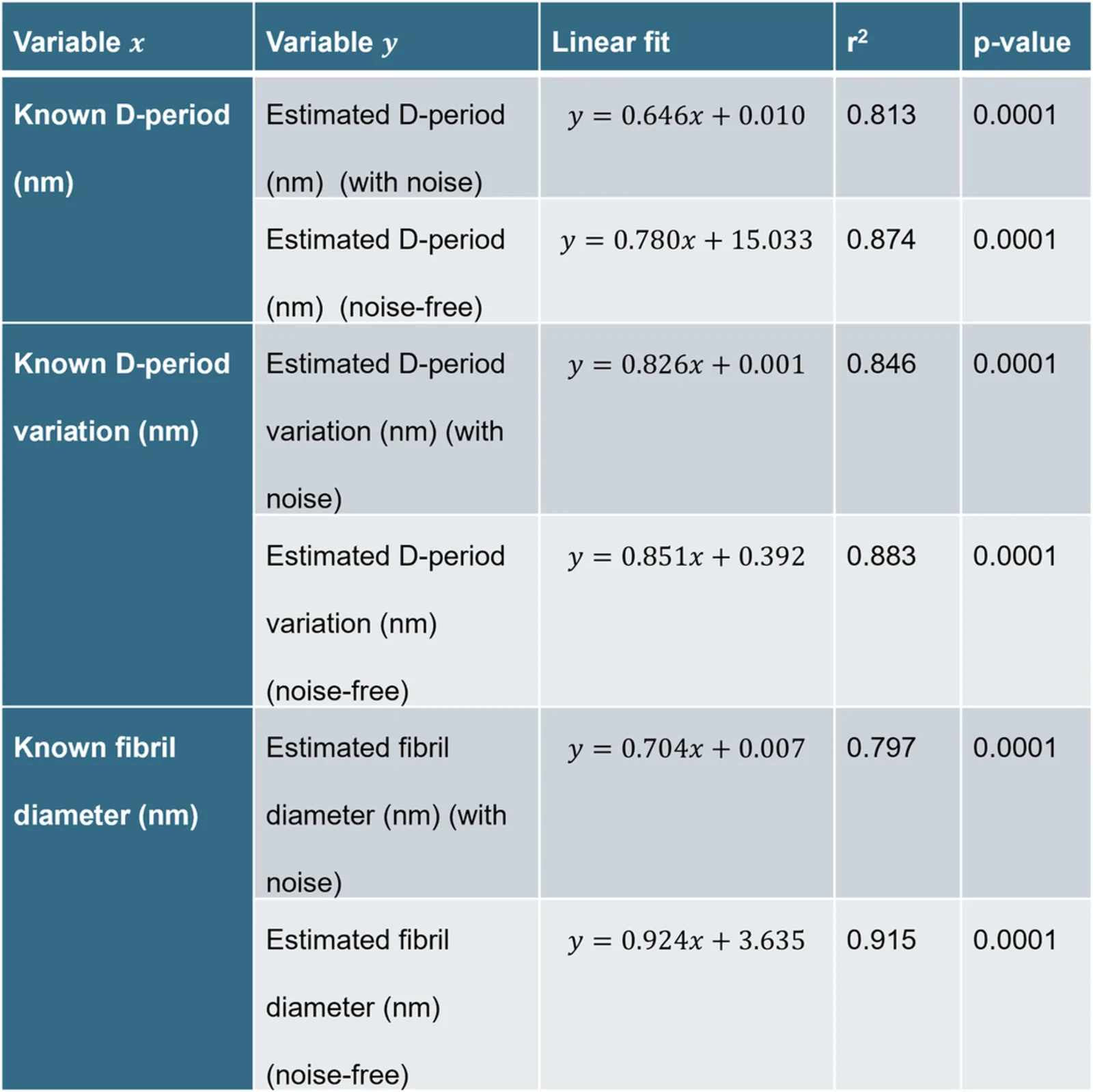
Two-sided ordinary least squares regression analysis of known digital phantom parameters versus estimated parameters from *TomoSAXS* with and without the addition of experimental noise

**Extended Data Table 2:**
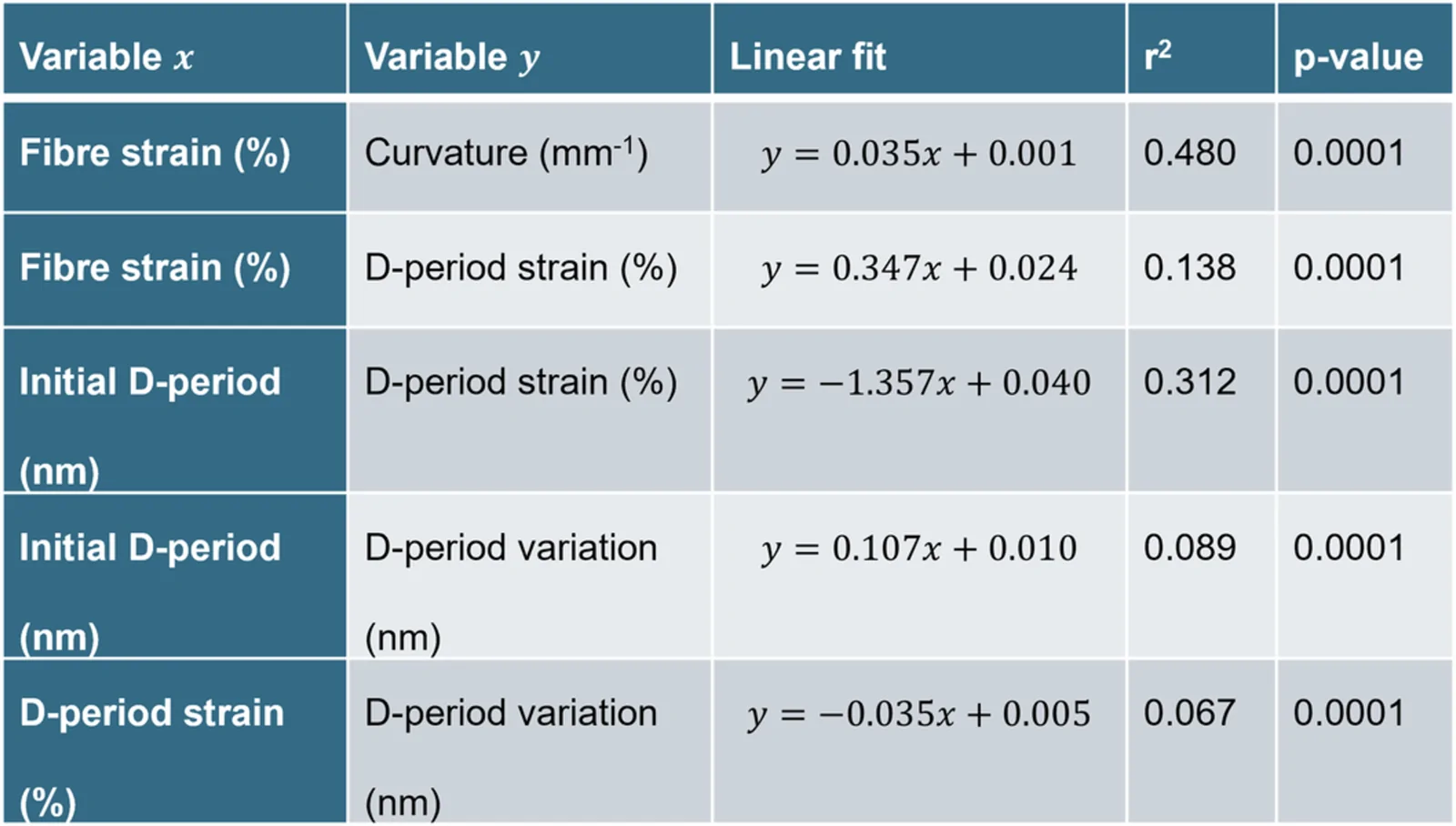
Summary of two-sided OLS regression analysis performed on per-fibre mean values

## Data Availability

An example dataset (rat IVD) is available (https://doi.org/10.5522/04/30286255), with all the data required to run TomoSAXS reconstruction for a single slice. Any supplementary information in this study is available in the paper and the Supplementary Information. Additional data are available from the corresponding authors upon request.

## Code availability

All code created for this project is available in a dedicated GitHub library (https://github.com/DiamondLightSource/tomosaxs).
