# Supplementary material for: Multimodal X-ray imaging reveals hierarchical fibre mechanics
Source: Nature. 2026 Nov 4;659(8139):91–9. doi: 10.1038/s41586-026-11148-5 (PMC13736667; doi:10.1038/s41586-026-11148-5)
Supplement: Supplementary file 1 [file 41586_2026_11148_MOESM2_ESM.pdf]

nature portfolio

Peer Review File

Multimodal X-ray imaging reveals hierarchical fibre mechanics

Corresponding Author: Professor Himadri Gupta

Any redactions in this file are there to maintain patient confidentiality, the confidentiality of unpublished data, or to remove third-party material.

This file contains all reviewer reports in order by version, followed by all author rebuttals in order by version.

Version 0:

Reviewer comments:

Referee #1

(Remarks to the Author)
The manuscript of Newham et al. describes a multimodal X-ray imaging approach combining phase-contrast tomography resolving the micrometer sized collagen fibers and SAXS to obtain nanostructural information on the fibril level. The method describes the deconvolution of the SAXS signal in a three-dimensional object to single fiber information. By measuring before and after mechanical loading they obtain the tissue, fiber and fibril strains in the connective tissue of an intervertebral disc (IVD).
It is a very interesting and novel work which allows to study multi-scale structural information at relatively low X-ray doses making in-situ mechanical studies feasible. The manuscript is very dense combining elements of method development with results on the IVDs. The motivation for the development of the method and it's application to the IVD are clearly stated and present a convincing case. The details of the method development together with results on the IVD is very interesting and novel, however it's presentation is not easy to digest, in particular for the targeted broad scientific audience. The references are reflecting appropriate credit to previous work. Thus I believe the manuscript deserves publication in Nature, but needs clarifications and also a more focused presentation together with a clearer description of the limitations.

Some main points to consider:
The authors should consider to shorten the manuscript to the essential key messages, some more technical details of the method developments might be better placed in a more specific journal as it would also be a pity to "hide them away" in the supplementary material. It is nice to see that the authors put a lot of effort into validating their methods with DVC phantoms and by comparing results with 2D SAXS and SASTT, but the conclusions aren't always clearly stated (see comments later on). Similarly with the presented results, for example in the extended data figure 2, every parameter which can be obtained is shown, but some of them are not introduced or discussed in any detail, which then makes it hard to grasp the most important findings.

The reconstruction to deconvolve the scattering from individual fibers relies on finding a first beam path in which one can clearly isolate the scattering of a few fibers which then allows to deconvolve the signal from additional beam paths allowing iteratively for more and more single fibrils to be indexed. For Figure 2 where this is illustrated it is claimed that in the highlighted path there are only three fibers which predominately contribute to that measured SAXS signal. It is clear that a fiber only contributes to the signal depending on the 3D orientation if Bragg condition is fulfilled, but in figure aii) it appears there are many neighboring fibrils in the same path which are all aligned in almost the same direction, therefore it isn't obvious that the condition fulfilled? Please comment and clarify this point!
It is stated that they manage to deconvolve 55% of the fibers, but the map of them (and the missing 45%) is not shown. The fibers which are not indexed might be from more dense areas, which opens up the question on the interpretation of the mechanical results. If no information is obtained from dense areas, which actually might be very important mechanically, what implications does it have on the discussed results? How can the relevance of the mechanics be estimated in the case of sparse inhomogeneous sampling?
Also related to the algorithm, it would be interesting to know how many fibers typically were in the beam path. In the method section it is stated that 10-20% are reconstructed by single fibre fitting, what happens then? How much double fibre fitting? It would be interesting to see the cascade and some discussion on what that means towards limitation of the method on other

samples.

In the concluding sections it is commented how this method could be used for even bigger samples and many other fibrillar materials which seem to make it even harder to find enough “sparse” beam paths where only few fibers contribute to the signal. Only the increase in data size is discussed for upscaling to human IVD’s (orders of magnitude bigger in size). But also the number of fibers in the beampath are increased considerably (longer beampath and bigger beam). How many single fibre fitting can be expected in that case and what percentage of fibers could be reconstructed reliably? It would be very important to discuss those limitations towards upscaling.

Some minor comments:
What is the current state of research in the microstructural understanding of the IVD? The structural data is only compared to other collagenous tissues later.

Figure 1 gives a nice overview, it would help to include a coordinate system explaining the “lateral angle” beta and later introduced “vertical angle” alpha and z-averaging.
It was a bit confusing what the coarse SAXS maps (for alignment?) and fine maps are corresponding to and at the same time comment on beam/step size together with the dimension of the sample. This would help to understand how sparse that sampling is compared to Crowther criterion of CT sampling of number of projections = Pi*diameter/resolution. On line 96 a SAXS voxel size of 20um is given, however it is not reconstructed in a voxel-based algorithm. Only 9 projections recorded, so apart from using a single axis as it is stated, that is also far less projections compared to SAXS tensor tomography reconstructions, so I am not sure how to understand voxel size in this context.

The novelty of the here presented TomoSAXS is to get SAXS information on a single fibre level, rather than for an average as a voxel size in SAXS tensor tomography. I would argue however that the main advantage is to do that with less dose, one could imagine that one can also deconvolve multiple fibre contributions within one voxel of a SAXS tensor tomography reconstruction and with that end with the same information. That could even help lifting some limitations of needing enough sparse beam paths to get the iterative indexing of fibers going. It would be interesting to compare the radiation dose to demonstrate the advantage of TomoSAXS in that respect.

Figure 2 ciii): The double fibre fit seems straight forward if the overlapping peaks in the azimuthal direction are separated in the radial direction but what happens if it’s not the case? The two apparently very different scenarios are not discussed in Methods-Module 4.

On line 113 some sub-sections along q are mentioned, not sure what that is, is it 3 azimuthal profiles in the range? Isolated angularly independent scattering instances are identified with maximum 3 fibers contributing predominately, but only in a subset of the scattering point? How many fibers can then be in the beam path that this criteria is fulfilled?

Extended data figure 2: as already mentioned as example of “too much information” consider to reduce, or structure better indicating subfigures with SAXS parameters and DVC parameters. Curvature is reported, but how is that calculated? It seems unnecessary to use different color scales for all the parameters in particular since some are still very similar.

Validation of the method:
Comparison with 2D SAXS (ext. data. Fig 6, S.I Note 19): As mentioned the key points are that there is less averaging/overlapping and thus the fitting is better, the figure is complicated to convey this. Some details to the figure:
align x-axes between (ai) and (aiii), Legends missing in (bii) and (biii)
Not sure about (bii) and (biii), why is the combined peak position higher than the 3 individual simulated fibres in (bii), and it is unclear what (biii) is.
Check if the caption is correct: (bi) should be (bii) and (bii) should be (biii) ?
One could assume it all depends on the number of simulated fibres vs number of frames?

Phantom comparison (ext. data. Fig 7, S.I note 18): This is very valuable comparison to check the reconstruction of the SAXS parameters, and the results are convincing. Maybe the precision could be reported as relative values to the variation between the fibres, rather than absolute. This does not validate the phase-contrast fibre identification part though.

Comparison with SASTT (ext. data 8, S.I note 20):
The 3D RSM is simulated for 3 fibres, and a SASTT reconstruction is simulated using a q-resolved spherical harmonics reconstruction. Then, how is the SASTT RSM analysed in (aiii) ? One gaussian is fitted to the spherically averaged intensity? In that case, this would be the same validation as in the 2D case. What about directly fitting the intensities in the individual fibre directions within a voxel, that could also be used to then further deconvolve single-fiber contributions?

In summary this is an impressive piece of work for which we would like to congratulate the authors. With a clear description of limitations and by presenting the key messages more clearly, we recommend publication.
This review has been performed as co-review.

(Remarks on code availability)

Referee #2

(Remarks to the Author)
I co-reviewed this manuscript with one of the reviewers who provided the listed reports.

(Remarks on code availability)

Referee #3

(Remarks to the Author)
This paper describes the use of TomoSAXS to map fibril to fibre mechanics across intervertebral discs. A large amount of work is presented for which the authors should be commended. The results are novel and would be of interest to any researcher interested in the fundamental biomechanics of intervertebral discs. The insight into the interaction between structures across scales in unique. I have several points that should be addressed before publication:
1. Line 15 – A reference to support the claim that injury and age related degeneration are key drivers for backpain. I think the relationship is more complicated than is suggested.
2. Line 22 – I think you should say here that you are talking about both animal and human IVDs.
3. Figure 1 – small comments but the ant-post arrow on ai could better align with the anterior and posterior regions of the disc shown. At the moment the arrow looks more cranial-caudal.
4. Line 154 – It should be stated that ref 24 is in human discs, not rat. Since they are not both rat, should we expect the strains to be similar? Also DVC errors are usually largest at the endplates. Does this mean you can't trust your result that the strains were higher in this region? Later, you explain your zero-strain study, but you don't give any indication that errors are different in different regions of the discs, which they will be, especially close to the endplates.
5. Line 169 – has it previously been shown that fluid flow from NP into AF is seen in this sort of loading and that it creates swelling? Is there any literature you can cite to back up this idea?
6. Line 171 and 172 – Standard deviations should be added to these average directions.
7. Lind 171 – Can you explain in more detail how the two angles for compression and tension indicate substantial strain accommodation by the extrafibrillar matrix and inter-fibre motion. I think readers would appreciate this being explained more clearly.
8. Line 200 – It would be useful to compare these stresses to the stresses you would expect failure. Have these failure stresses been previously quantified? Are these stresses close to failure, or a long way off?
9. Line 362 – I expect immersing these specimens in PBS would result in over-hydration. How did you prevent overhydration? Was the hydration level of discs physiological at the point of scanning?
10. Line 400 – could you add justification as to why you chose to axially compress specimen, rather than axially rotate or flex?
11. Line 406 – These calculations to convert an applied load to an internal pressure would be more trustworthy if the fact that NP pressure differs from AF pressure. So this simple conversion is not an accurate method to compare to pressure measured in-vivo (in the NP region).
12. Line 408 – How did you know that 20 minutes stress relaxation was sufficient? Justification required.
13. Line 409 – can you give more detail on how the samples were clamped into position. How did you make sure you did actually hold the same position? I would expect this is challenging.
14. Line 413 - what was your definition of just touching? it is usual to use a load for this threshold to ensure this step is repeatable.
15. Line 415 – how was the 50 micron displacement applied? This is not explained in enough detail. Was this loading purely axial? How did you make sure? Was the mid-transverse slice of the IVD parallel to the loading plates? How did you make sure of this? If you couldn't, how would any errors in this affect your results?

(Remarks on code availability)

Referee #4

(Remarks to the Author)
This is a very exciting approach combining tomography and SAXS in situ, where the novelty is the presentation of a data handling approach named TomoSAXS, enabling mapping fiber to fibril mechanics within the tissue. The manuscript is very well written and clearly outlined.

It is clear that TomoSAXS as a reconstruction approach to gain local fiber and fibril strain (micro to nano scale) has high potential, and the authors present a very thorough work. However, key aspects of validation using more controlled experiments are largely missing. Despite the IVD being a compelling tissue, it presents an extremely complex case for fundamental proof of concept of a new approach. After proper validation, it is clear that TomoSAXS may gain importance is studies of fibrous materials with 3D structure and variabiity.

Specific comments are presented below.

- Despite including several experiments, there seems to be a lack of basic validation of the theoretical approach, e.g. showing that TomoSAXS is valid on a known sample with known fiber and fibrillar structure and distribution. The current

validation includes simulated phantom data, simulated comparisons with SASTT and comparisons with in situ 2D scattering. For the latter, the match is not very good, which is inherent as TomoSAXS aims to analyze the full 3D structure (and should not match the 2D results). Despite the data seeming reasonable, much of it is very noisy and it is not possible to understand if and how much of this is simply due to true biological variability or how much is originating from theoretical limitations of the TomoSAXS fitting.

- Uncertainty assessment: A large part of the presented data is very noisy, see e.g. the correlations between fiber strain and fibrillar strain (section starting on line 184, and figure 3, and extended data figure 4). The reasons for this is unclear. Again, is it biological variability, or is it in the tomoSAXS or due to uncertainties in other parts of the pipeline? How are the uncertainties in DVC and its inherent noise contributing? Is it possible to assess the propagation of uncertainties from CT-DVC-SAXS and tomoSAXS approach?

- The main experiments are performed on rat IVDs in situ. Then, a human experiment is included as preliminary data of TomoSAXS, but in my understanding it is a static combined tomography and SAXS experiment? Thus it is not including in situ loading? (line 257 etc) It is also a formalin fixed specimen (line 691). The role of this part in the paper is unclear. In my opinion, nature papers should not include preliminary findings (It should either be in and then fully analyzed to align with the aims of the study, or it should be removed). Currently the relevance without in situ and of fixed specimens are unclear.

- Scan time is presented as a limitation, but the total time for the testing of one rat IVD is not presented. What was the testing time from the sample went into the rig for the first step to the end? This should go into the extended data figure 1, and text.

- Radiation dose is estimated, which is important. Please present also total dose. If my estimations are correct the total dose would be somewhere around 72 kGy + additional dose from alignment x 4. This is a substantial dose and should be acknowledged and discussed.

- Line 334 states that about the future prospects of the technique with focus on soft biological tissues. Considering the mechanical challenges of soft tissues (viscoelasticity), long duration of the experiments and the high radiation dose, the reliability of the fibril strain may be questionable. It would be nice to hear the authors thoughts about the future potential.

- How sensitive is TomoSAXS reconstruction to number of SAXS projections, and beamsize? How large can the discrepancy between resolution of CT and SAXS be, without losing the ability to reconstruct the 3D fibrillar data?

Minor comments

- Aim: The introduction and motivation of the work is presented clearly and compellingly. However a clear aim/hypothesis/research question is missing.

- Many of the figures are presented with colors and font-sizes that makes it impossible to view and read the details.

- Extended data figure 5 (and also part of 6) is unreadable and it is also unclear from the figure legend what is shown. What lengthscale? What studies? What is FIVD 1-3?

- For extended data figure 6 b-c, did you try any sample with a simpler fibril orientation in 2D, thus where you would expect your TomoSAXS to result in similar pattern as the 2D direct SAXS mapping?

- The application of digital phantoms is a very nice approach to simulate noise in the data and show accuracy of added noise.

- One possible validation experiment could be to compare with SASTT in a downsampled situation. If you consider the same volumes, how do the techniques correlate then?

- In the experiments, how similar were the IVDs in behavior? Why was the experiment done in displacement control instead of strain control?

- The overview of the TomoSAXS algorithm is hard to follow. Could a schematic of what goes into the different modules help? Also here, the total time for computation of the data is unclear? 6-7 days + 4-5 days plus ??

- Statistical analysis: This section is not possible to assess. It is unclear what is compared and it reads does not read as a methods section. Why were the methods selected? What was compared? Please motivate the approach and explain what is presented.

Supplementary information

- Supp table 1 – first time mentioning number of samples. Unclear if they are all from the same rat or three different rats? Why did you not use the same IVD/spinal segments to also gain some idea of reproducibility? Also why did the strain vary so much?

- Supp note 9: It is important to assess zero strain noise. However, in this experiment you also move the sample across DLS

during the experiment. Did you do any zero strain analysis including moving the sample in and out? It would give a better understanding of the uncertainties involved.

- Supp note 19: Includes statistics that has not been introduced in the statistics earlier. No motivation of type of data and type of statistical testing chosen.

(Remarks on code availability)

Version 1:

Reviewer comments:

Referee #1

(Remarks to the Author)
The revised version of this manuscript by Newham et al., reporting on a multi-modal X-ray imaging approach combining phase contrast tomography and SAXS convincingly includes the novel method, including all necessary details and validation, as well as the results on animal and human intervertebral disc.
The comments made on the first draft have all been carefully and exhaustively answered. The balance to make this manuscript accessible to the broad audience of nature while including all necessary details has been worked on very well. New illustrations and schematics are greatly aiding the understandability. The manuscript is still very dense, and at parts not easy to digest, but this should not be taken as a critique, but is rather a result from merging novel method development with interesting findings on biological application, which of course does increase the significance and potential impact of this publication. I would like to congratulate the authors for an impressive piece of work.
While there was already significant work shown for the validation of the method in the fist version, the authors have now extended even further by introducing a tendon which due to unidirectional orientation provides an easier test case. The authors have also added discussion towards the very low dose needed compared to other approaches, which highlights even more the significance in particular towards in-situ measurements of mechanical test on radiation sensitive samples. Limitations of the method are appropriately discussed and conclusions drawn are carefully considering those.

one very minor detail to cross-check: in the rebuttal letter 1.6 II. It is stated in the answer that the solved fibers are in the range of 25-60%, in the changes made to the manuscript it is stated that 35-85% of all detectable fibers were solved, check which number is the correct one to be added to the manuscript.

(Remarks on code availability)

Referee #2

(Remarks to the Author)
I co-reviewed this manuscript with one of the reviewers who provided the listed reports.

(Remarks on code availability)

Referee #3

(Remarks to the Author)
I believe the authors have addressed most of my comments satisfactorily. There are three, however, listed below that I believe require a further response:

1. Line 237 – “This can be interpreted as fibres with an initially tensile pre-strain undergoing compression upon loading, and those with an initial compressive pre-strain undergoing tension”. My understanding is that fibres can only resist tension so this wording is strange to me. Is it possible for fibres to withstand compression? Do they not just buckle? Please could you expand your discussion here to explain to other readers who may have the same assumption about fibres only resisting tension?

2. Line 243 – “…residual stress in the IVD may result in fibrillar strains close to failure” – Do you mean fibrillar stresses close to failure in this sentence? And do you think it makes sense that residual stresses are so high? I note that this has been reported in other tissues, but it seems strange to me that the stresses are so close to failure at quite a low load. Why would this be beneficial from a physiological perspective? I would have expected these stresses to be substantially below the failure stresses, so I believe the manuscript should include additional discussion on this.

3. Comment 3.12 from previous round – “20 minutes stress relaxation” – What was your definition of “stabilise” (in manuscript text) or “plateau” (in your rebuttal). I.e. less that X% change in X minutes.

(Remarks on code availability)

Referee #4

(Remarks to the Author)
I would like to thank the authors for the extensive response to all reviewers comments and for clearly addressing all queries. The manuscript and work represents an excellent contribution to the scientific community!

There are still a number of figures in extended data where the font sizes are not readable, which would benefit from minor editing.

(Remarks on code availability)
I have downloaded and browsed the code, but I have not fully understood it or spend enough time to state that I have reviewed it.

Open Access This Peer Review File is licensed under a Creative Commons Attribution 4.0 International License, which permits use, sharing, adaptation, distribution and reproduction in any medium or format, as long as you give appropriate credit to the original author(s) and the source, provide a link to the Creative Commons license, and indicate if changes were made.
In cases where reviewers are anonymous, credit should be given to 'Anonymous Referee' and the source.
The images or other third party material in this Peer Review File are included in the article's Creative Commons license, unless indicated otherwise in a credit line to the material. If material is not included in the article's Creative Commons license and your intended use is not permitted by statutory regulation or exceeds the permitted use, you will need to obtain permission directly from the copyright holder.
To view a copy of this license, visit https://creativecommons.org/licenses/by/4.0/

We are very grateful to all Reviewers for their insightful, constructive and supportive comments on our manuscript. In the response below, we highlight Reviewer comments in **blue**, our response in **black**, and changed text in the revised manuscript in **red**. Reviewers are listed as R1, R3 and R4, since the first review (R1+R2) was reported as a co-review.

Referees' comments:

Referee #1 and Referee #2 (Remarks to the Author):

1.1 - The manuscript of Newham et al. describes a multimodal X-ray imaging approach combining phase-contrast tomography resolving the micrometer sized collagen fibers and SAXS to obtain nanostructural information on the fibril level. The method describes the deconvolution of the SAXS signal in a three-dimensional object to single fiber information. By measuring before and after mechanical loading they obtain the tissue, fiber and fibril strains in the connective tissue of an intervertebral disc (IVD).
It is a very interesting and novel work which allows to study multi-scale structural information at relatively low X-ray doses making in-situ mechanical studies feasible. The manuscript is very dense combining elements of method development with results on the IVDs. The motivation for the development of the method and it's application to the IVD are clearly stated and present a convincing case. The details of the method development together with results on the IVD is very interesting and novel, however it's presentation is not easy to digest, in particular for the targeted broad scientific audience. The references are reflecting appropriate credit to previous work. Thus I believe the manuscript deserves publication in Nature, but needs clarifications and also a more focused presentation together with a clearer description of the limitations.

We thank the reviewers for their positive assessment of our work, and recommendation that our manuscript "deserves publication in *Nature*". We have worked on clarifying their specific points as discussed below, added new Figures and analyses, and improved the presentation to make the manuscript more appropriate for a broader audience. A clearer description of limitations has been added to the discussion.

Some main points to consider:
1.2 - The authors should consider to shorten the manuscript to the essential key messages, some more technical details of the method developments might be better placed in a more specific journal as it would also be a pity to "hide them away" in the supplementary material.

Thank you for this comment. We have moved technical details out of the main manuscript into the methods. These include details of DVC calculation, detailed technical descriptions of the *TomoSAXS* reconstruction algorithm, and human *TomoSAXS* acquisition and analysis.

1.3 - It is nice to see that the authors put a lot of effort into validating their methods with DVC phantoms and by comparing results with 2D SAXS and SASTT, but the conclusions aren't always clearly stated (see comments later on). Similarly with the presented results, for example in the extended data figure 2, every parameter which can be obtained is shown, but some of them are not introduced or discussed in any detail, which then makes it hard to grasp the most important findings.

We thank the reviewers for appreciating our efforts to validate our methodology. We have now attempted to further clarify the results of our validations, through placing a dedicated validation and uncertainty section at the start of the results (lines 144-171).

Extended data figure 2 (now 4) is deliberately designed to represent all the inherent outputs of the technique. This is to support other researchers in understanding the full potential of the *TomoSAXS* technique and how it could be applied to their own research. We appreciate R1's comment that embedding this figure within the main manuscript may distract the reader from the most important findings, which is why it has been designated as an extended data figure, which will not appear within the main body of text upon publication. We have now moved extended data figures to the end of the main manuscript in our resubmitted document, to better reflect how the paper will read upon publication.

1.4 - The reconstruction to deconvolve the scattering from individual fibers relies on finding a first beam path in which one can clearly isolate the scattering of a few fibers which then allows to deconvolve the signal from additional beam paths allowing iteratively for more and more single fibrils to be indexed. For Figure 2 where this is illustrated it is claimed that in the highlighted path there are only three fibers which predominately contribute to that measured SAXS signal. It is clear that a fiber only contributes to the signal depending on the 3D orientation if Bragg condition is fulfilled, but in figure aii) it appears there are many neighboring fibrils in the same path which are all aligned in almost the same direction, therefore it isn't obvious that the condition fulfilled? Please comment and clarify this point!

We thank the reviewers for this comment, and appreciate that, in Figure 2a, more fibres appear to be fulfilling the diffraction condition than the three highlighted. Indeed, inspection of the simulated intensities in Fig. 2ci shows an additional 5 fibres are contributing to the overall pattern. However, due to a combination of: 1) their 3D orientation and: 2) position or distance from centre of the approximately Gaussian shaped beam profile, their intensity contributions are significantly smaller than the 3 highlighted fibres.

We appreciate that it is not immediately obvious from the 3D renderings that these 3 fibres are the only 3 predominant contributors to the scattering signal. We attribute this as due to the limitations of displaying 3D data in 2D, and the colourmaps used to display alpha and beta angles covering the full 0 to 180 degrees making it difficult to visually distinguish small variations in orientation. We have tried multiple ways of displaying the beam path in Fig. 2, and ultimately found these renderings best display the complexity of the beam path. To note the effect of fibre position relative to beam, we have expanded our explanation:

Lines 118-119: "due to their 3D orientation and anisotropic diffraction, as well as their distance from the centre of the Gaussian beam profile, only a subset of these fibres will satisfy the Ewald diffraction condition and visibly contribute to the SAXS pattern"

1.5 - It is stated that they manage to deconvolve 55% of the fibers, but the map of them (and the missing 45%) is not shown. The fibers which are not indexed might be from more dense areas, which opens up the question on the interpretation of the mechanical results. If no information is obtained from dense areas, which actually might be very important

mechanically, what implications does it have on the discussed results? How can the relevance of the mechanics be estimated in the case of sparse inhomogeneous sampling?

1.6 - Also related to the algorithm, it would be interesting to know how many fibers typically were in the beam path. In the method section it is stated that 10-20% are reconstructed by single fibre fitting, what happens then? How much double fibre fitting? It would be interesting to see the cascade and some discussion on what that means towards limitation of the method on other samples.

In these comments, Reviewers 1-2 ask important questions about our technique, specifically **how many fibres we reconstruct**, whether the results show **only SAXS reconstruction in less dense fibrous regions**, and **whether it can work for denser, larger, or more aligned structures**, where many fibres might be contributing to the SAXS signal at a particular c-orientation. We address these in a 3-part response below:

I. **Angular resolution**: First, part of the reason for not reconstructing all the fibres was the choice of an angular step size in the *in situ* rat IVD experiment to 22.5° (9 rotations across 180°). An unforeseen accelerator-related beam intensity reduction (due to an issue with a radiofrequency cavity at Diamond Light Source) resulted in ~2/3 the usual X-ray flux. This resulted in longer exposure times, necessitating a reduction in angular sampling steps while keeping the scan x-y extent the same.

   The lower number of rotations were chosen to attempt to match potential for fibre reconstruction with efficiency for scanning multiple samples before and after loading during the available beamtime. The resulting step of 22.5° is relatively large compared to the equatorial angular peak width of the SAXS reflection ~ $q_0 \tan(w_\mu)$ and, as the Reviewer notes, much larger than usual SAXS-TT step sizes. Increasing the angular step resolution will increase the number of fibres that fulfil the Ewald diffraction condition, and thus the potential number of solvable fibres.

   In the revised MS, we demonstrate this with model *TomoSAXS* simulations of the fibre geometry of our scanned samples with unit amplitude and mean fibril parameters (e.g. w_μ = 0.2), at different numbers of total rotations (3, 6, 9(used), 12 and 18). Using a threshold of 10% of maximum measured scattering intensity, we have calculated the proportion of fibres that never meet the diffraction conditions to satisfy this threshold in each scenario. Results show that with increased angular resolution (number of rotations), the proportion of fibres that contribute to the signal increases from ~72% at 22.5 degrees to 96% at higher angular resolutions of 10° (new Supplementary Fig. 5a, shown below).

   Therefore, by increasing the angular resolution to >18 rotations, *TomoSAXS* is in principle able to reconstruct and retrieve over 95% of fibres in more optimal scanning conditions, **showing it is not an in-principle technique limitation of *TomoSAXS* at current sizes**. We also note that, conscious of this limitation, we took care to use a smaller (7.5°) angular step for the ESRF human IVD experiment.

   Changes made in text:

   We have added text in the Discussion section, and in Methods, and a new Supplementary Figure, in which part A) shows this behaviour:

   Discussion:

***Line 341:* "Here, the relatively large angular step (22.5°) used for rotations in the rat IVD case (*Methods*) limited the number of fibres detectable under Ewald conditions, which can be improved in future by higher angular resolution (5°-10°; Supplementary Fig. 5a). …"**

Methods:

***Line 428-432:* "A relatively large step size of 22.5° was used due to a significant reduction in flux caused by synchrotron accelerator issues during the beamtime. This required fewer scan steps to test all samples. The experiment can be readily scaled to a smaller angular step (greater fraction of fibres intersecting the Ewald sphere)."**

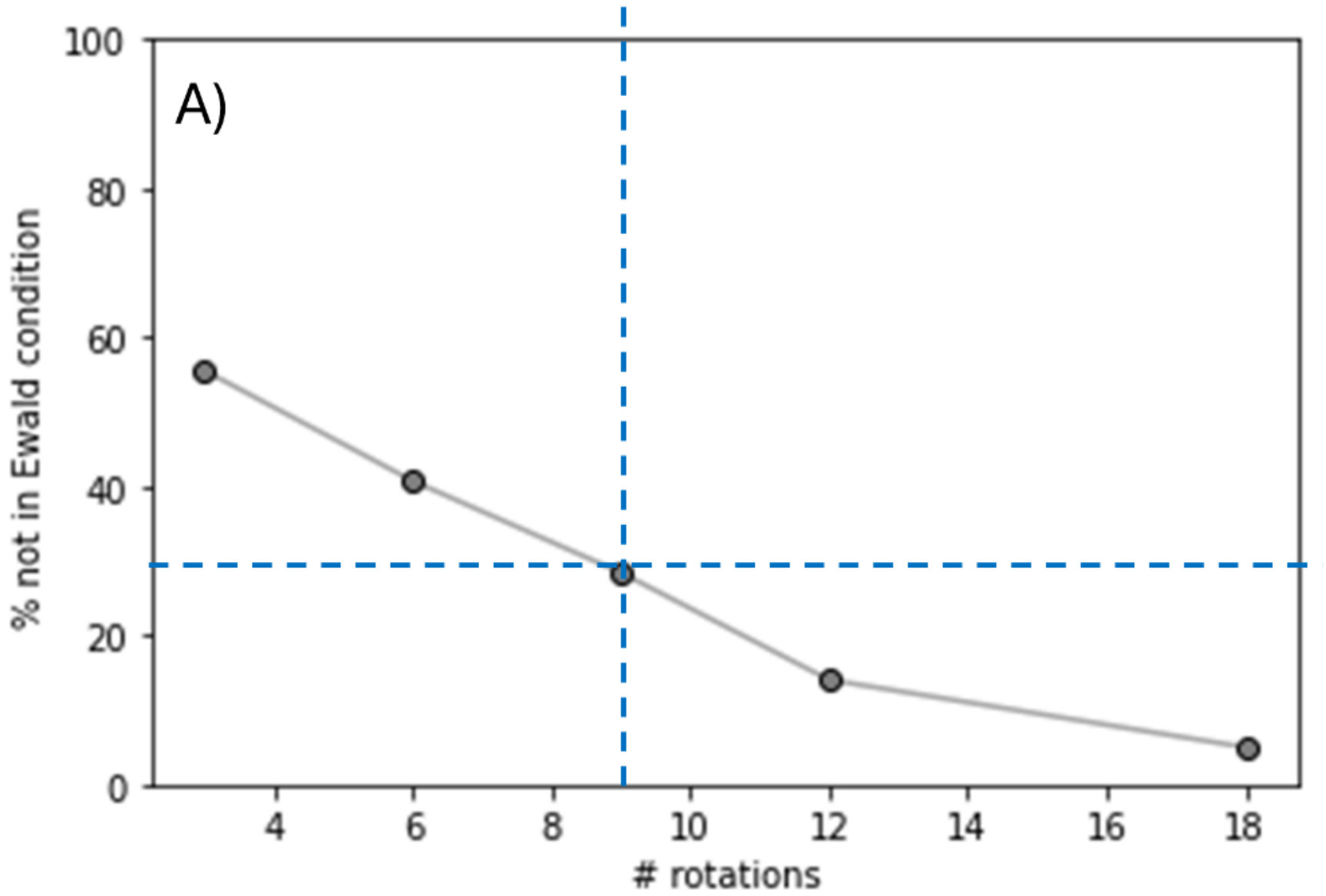

Supplementary Figure 5: *A) The percentage of fibres which do not scatter significantly decreases with increasing number of rotation steps in a 0-180° SAXS tomographic rotation scan (<10% peak intensity; real sample fibre geometry used). Vertical dashed line: 9 rotations (used for in situ rat IVD experiments) corresponding to ~30% fibres. By 18 rotations, <5% of fibres do not scatter significantly.*

II. **Sampling across all zones, dense regions, solved versus unsolved**: A potential danger which the reviewers highlight is whether our method can miss certain tissue regions due to unfavourable diffraction conditions or dense alignment. The reviewer asks us to show the fibres which are solved versus the unsolved fibres, and to check that we still obtained information from dense regions.

The coverage of solved versus unsolved fibres across the slices is now displayed in a new **Supplementary Figure 6** (below). Here, greyscale texture shows solved fibres, cyan shows fibres which never entered diffraction condition with the given angular step size, and dark pink shows fibres with detectable scattering which remained unsolved. While some dark pink fibres are present across the slices, there is no evident region-specific absence of solved fibres, i.e. it is not the case that dense regions do not get solved using our method. The gap in the upper right is due to the presence of a vertebral

endplate, and we have, in this current implementation of *TomoSAXS*, focused on unmineralized collagen only.

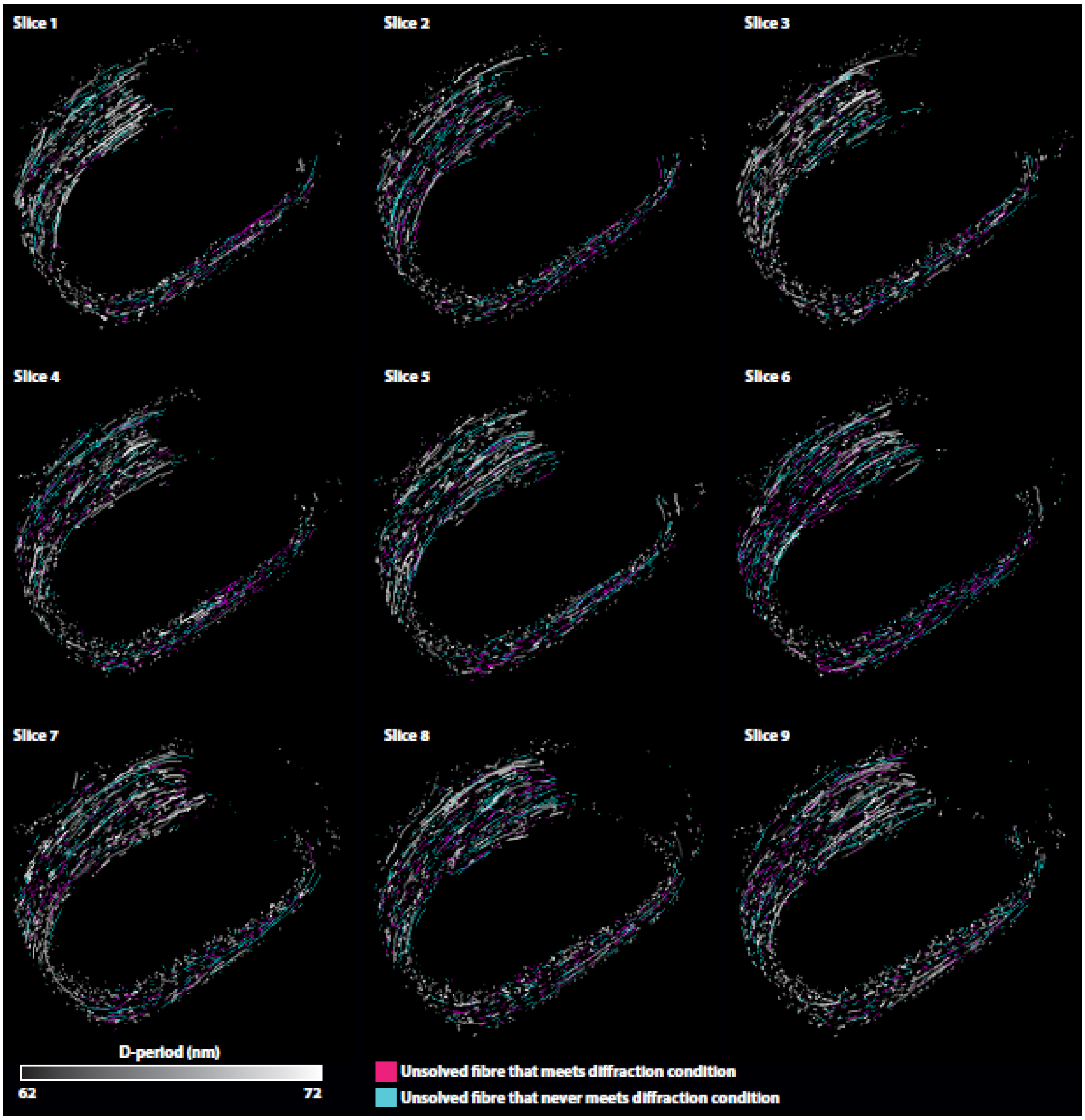

SI Figure 6: *Rendering of solved fibres (~56%, grayscale), fibres which do not meet Ewald diffraction condition (~27% as per Supplementary Fig. 5a; cyan), and fibres which scatter but remain unsolved in the TomoSAXS reconstruction (~20 %, dark pink). Upper right void is due to scattering from mineralized fibrils, which are left out of the current implementation of TomoSAXS*.

Percentage of fibres solved: 55% refers to the total number of distinct fibres (which span multiple SAXS slices) which are solved at least once across the 10 slices. Within individual slices, the fraction of solved fibres ranges from 25-60%.

Thus, we do obtain representative sampling of nanoscale fibre properties when considering the ROI, as evident from the lamellar-level texture seen by combining adjacent slices in Fig 3c. But we acknowledge that this may be at the expense of a vertical loss of resolution, due to this combination of adjacent slices.

Further, as noted in our later response to Reviewer 4 (**4.3**), our phantom simulations support the suggestion that, for the fibres which are detected, the parameters are being recovered reasonably accurately.

Changes made in text:
Figures: We add the **Supplementary Figure 6** as shown above.

Text: In the Discussion section, where limitations and future extensions are described, we now discuss this:
Line 344: **"***In each horizontal slice around 35-85% of all detectable fibres were solved, which were distributed reasonably uniformly (but not densely) across the tissue volume (Supplementary Fig. 6). To improve spatial resolution, future work may look to improve the SAXS reconstruction pipeline by adding higher-order overlaps to increase the fraction of solvable fibres or adapting alternate global solve strategies."*

III. **Fibres reconstructed, % solved, cascade, single/multiple etc.**: We show below a graph (Supplementary Fig. 5b) with the cumulative number of solved fibres per iteration, showing single-voxel (SV: green) and double/multiple voxel (MV: dark blue).

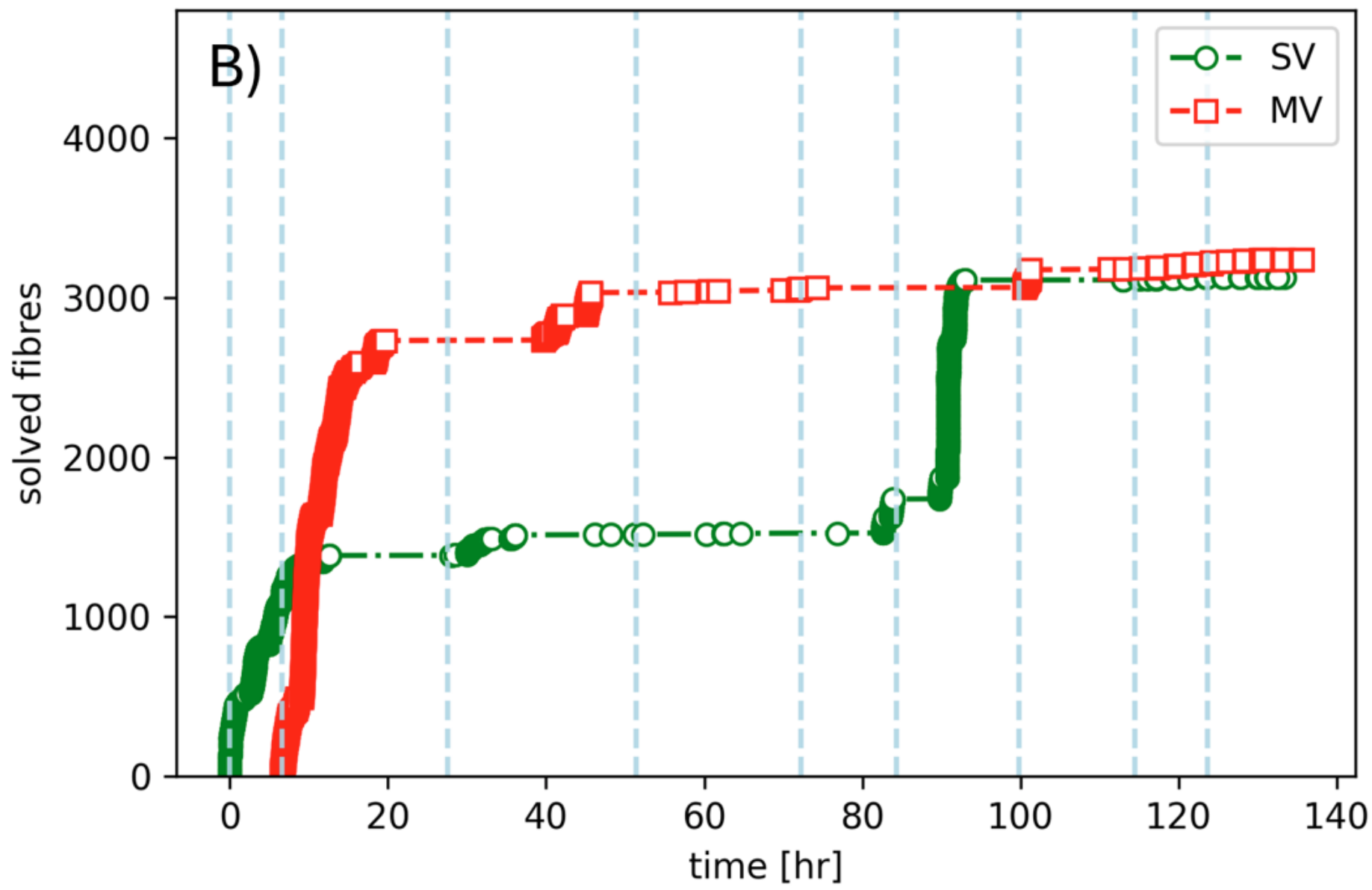

Supplementary Figure 5: **...** *B) Cumulative rate of solution of fibres for the FIVD1 sample geometry: green – single voxel (SV) solves; red – multiple voxel (double and triple) solves). Dashed lines denote a complete set of rotations (epoch). Symbols denote where solution occurred. Reconstruction terminates when no SV or MV solves occur during an epoch.*

...

The solution proceeds in two stages – first, A) a repeated SV-only sweep across all rotations and beampaths till no more SV fibres can be solved, followed by B) repeated sweeps with single-, double- and triple-voxel solves allowed. Both loops A) and B) continue till no more solved fibres were calculated in a full sweep across rotations and beampaths, indicating saturation. This process is summarized by a new flowchart describing the process (in response to **R4.17** comment – see below).

In the data shown, we see an initial rapid increase of SV-solved fibres, followed by a near stepwise increase of SV/DV/TV solved fibres, where an increase is followed by a plateauing/slowing down of the solutions. In the plateaus, a few fibres are being solved in every sweep through all rotations, but much fewer than before. At some point in the plateau, solution of a "keystone" fibre or group of fibres unlocks a cascade of newly solvable fibres, leading to another rapid increase and so on. This is repeated around 3-5 times in a typical run. The reconstruction terminates when no further fibres are solved in a sweep through all rotations.

While this approach would naturally take longer on larger samples (since the predicted scattering for each fibre must be calculated each time, and there is more overlap), it is not in-principle a bottleneck to larger samples. This is evidenced by our pilot studies on human data (**Fig. 4**), and we refer the Reviewer to that section for further information.

Changes made in text: We have added a new figure (Supplementary Fig. 5b) which graphs the cumulative number of solved fibres in single- and multiple-voxel case, showing the saturation and cascade behaviour described above.

We have also added the following text to Materials and Methods, section "*Overview of TomoSAXS algorithm*". Please also see the added flowchart (response to **4.17;** Supplementary Fig. 7).

Line 562: *Fibres are isolated at single-, double- or triplet level when one, two, or a maximum of three fibres contribute predominantly (>90%) to total scattering across at least a 10° sector around the circumferential χ axis of individual SAXS frames (Fig. 2ci). By calculating SAXS radial intensity profiles within this azimuthal sector at 3 equally spaced angular positions (e.g. at 0-3.33; 3.33-6.66; 6.66-9.99 degrees from the start of a 10° sector), and fitting each radial profile with 3D diffraction models, the parameters mean D-period (inverse to radial peak centre; Equation 1); variation in D-period (peak width along q); and fibril radius (inversely related to peak width along χ) can be estimated. For double and triple fibre fits, to avoid degenerate cases where multiple fibres are pointing in the same direction and with similar fibril parameters, we enforce a minimum separation of 5° between fibrils, as well as minimum predicted intensity levels from each fibre to avoid dominance by one (for doublet) or two (for triplet) fibres.*

To iteratively solve the fibre parameters, a two-stage process is followed (Supplementary Figure S7). First, a sweep is conducted across all rotations and beam paths to isolate and fit single fibres with sufficient independence in scattering intensity across χ, until no more individually isolatable fibres are solvable. The process is then repeated to include fitting for single, double and triple sets of fibres across all beam paths and rotations, looping till no further fibres are solved in a single sweep through all angles and rotations.

1.7 - In the concluding sections it is commented how this method could be used for even bigger samples and many other fibrillar materials which seem to make it even harder to find enough "sparse" beam paths where only few fibers contribute to the signal. Only the increase in data size is discussed for upscaling to human IVD's (orders of magnitude bigger in size). But also the number of fibers in the beampath are increased considerably (longer beampath and bigger beam). How many single fibre fitting can be expected in that case and what percentage of fibers could be reconstructed reliably? It would be very important to discuss those limitations towards upscaling.

As this point has been raised by both R1-2 and R4, we address the human reconstruction question in our consolidated response **in R4.5**. For our rat IVD samples, we present below an example histogram of the number of fibres per beam path. While the median value is high at around 20, the distribution is pronouncedly asymmetric to smaller fibre numbers, i.e. there are many beam paths with 1-5 fibres only. Of these even less would be satisfying the Ewald diffraction condition, and hence the chance of single- and multiple-voxel solves is high.

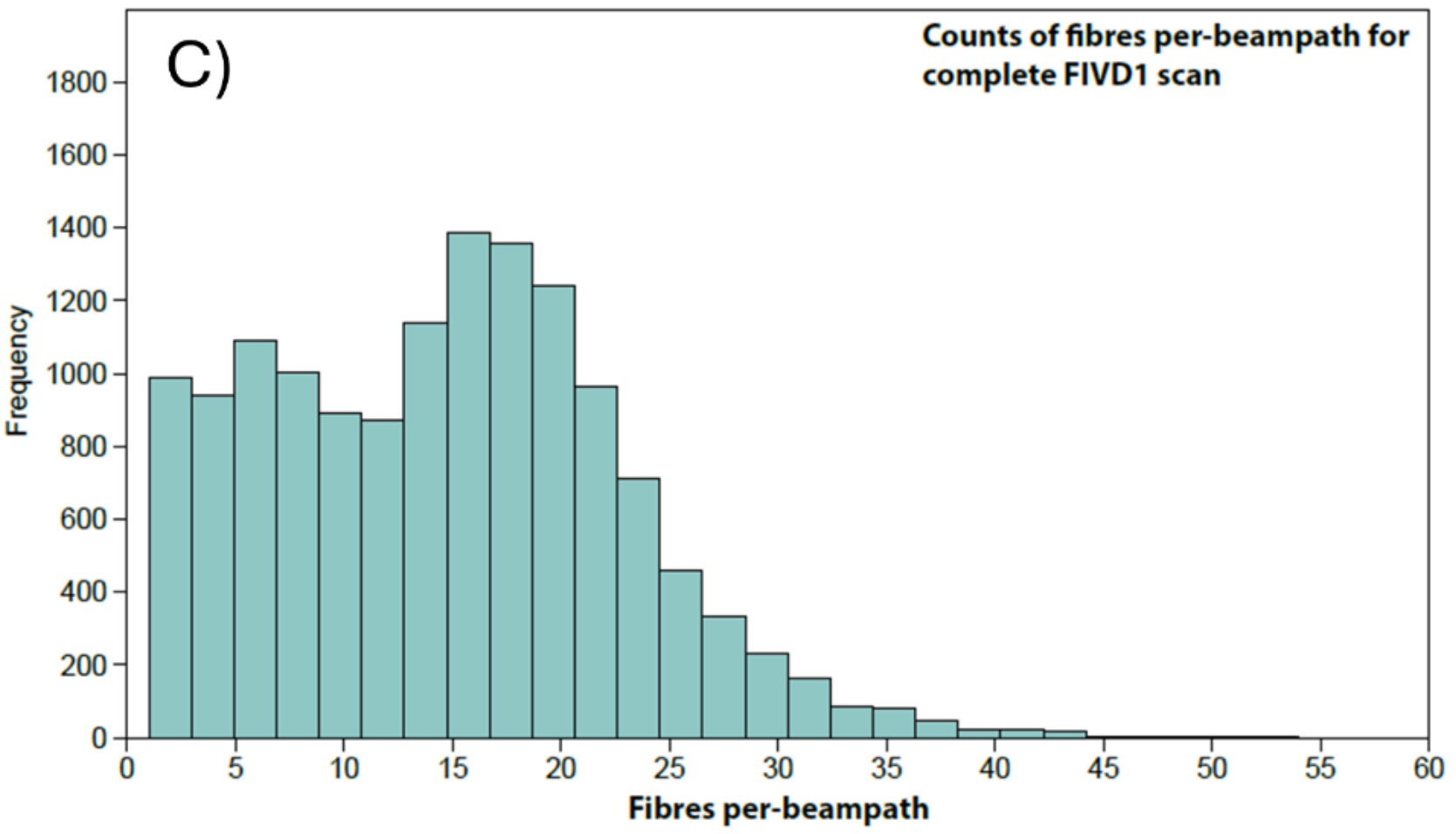

Supplementary Figure 5: **...** *C) Histogram of counts of fibres per beam path for a complete IVD1 scan*.

Some minor comments:

1.8 - What is the current state of research in the microstructural understanding of the IVD? The structural data is only compared to other collagenous tissues later.

We thank the reviewers for this comment and have added additional comments on the current state of the micro- and nano structural understanding of the IVD:

Line 35: "*Imaging of this fibrous microstructure across the whole organ is challenging due to the similar density of fibres and the surrounding matrix, and fibre analysis of the IVD has generally been limited to small regions of interest(Disney et al., 2019, 2022).*"

Line 52: "*2D SAXS analysis of the IVD has demonstrated that energy dissipation occurs through fibril reorientation and stretching(Rosenberg et al., 2023), but interpretation has been limited due to a lack of 3D information.*"

1.9 - Figure 1 gives a nice overview, it would help to include a coordinate system explaining the "lateral angle" beta and later introduced "vertical angle" alpha and z-averaging.

Thank you for this comment; we have added a coordinate system to Figure 1b and Fig. 2ai.

1.10 - It was a bit confusing what the coarse SAXS maps (for alignment?) and fine maps are corresponding to and at the same time comment on beam/step size together with the dimension of the sample. This would help to understand how sparse that sampling is compared to Crowther criterion of CT sampling of number of projections = Pi*diameter/resolution. On line 96 a SAXS voxel size of 20um is given, however it is not reconstructed in a voxel-based algorithm. Only 9 projections recorded, so apart from using a single axis as it is stated, that is also far less projections compared to SAXS tensor tomography reconstructions, so I am not sure how to understand voxel size in this context.

We thank the reviewers for this comment. As the sampling density was equal for both initial 2D SAXS raster maps used for sample alignment and the primary rotational raster maps, we have removed any mention of 'coarse' and 'fine' SAXS maps from the manuscript. We now instead refer to an alignment SAXS map followed by tomographic SAXS mapping. The reviewers are correct that this is not a voxel-based algorithm, so we have replaced any reference to a SAXS 'voxel' size with reference to the SAXS beam diameter.

We thank the reviewers for mentioning the Crowther criterion – we had not previously compared the number of projections we have used to the theoretical minimum number required for tomographic reconstruction, and doing so has strengthened the presentation of the advantages of *TomoSAXS,* showing that we require only 1 % the theoretical minimum number of projections for tomography. We now mention this in the discussion:

Line 334: "Additionally, *TomoSAXS* only requires ~1 % the theoretical minimum number of SAXS angular projections needed for tomographic reconstruction (Supplementary Note 23)."

And the following note has been added to the supplementary materials:

One of the principal advantages of TomoSAXS over existing 3D SAXS reconstruction techniques, such as SAXS-TT, is the substantially lower number of angular projections required for reconstruction. The theoretical minimum number of projections required to reconstruct a tomographic image is given by the Crowther criterion (Crowther et al., 1970):

$$N = \pi D / r$$

Where N *is the number of projections,* D *is the diameter of the sample, and* r *is the voxel size.*

For SAXS-TT, data must be acquired about two axes due to the orientation-dependent nature of X-ray scattering. As a result, the minimum number of angular projections is effectively twice that predicted by the Crowther criterion.

For the rat IVD experiment considered in this study, an equivalent SAXS-TT experiment of a 4 mm diameter sample using a 20 µm voxel size (corresponding to the SAXS beam diameter) would require 1,257 angular projections. In contrast, the TomoSAXS experiment used only 9 projections, corresponding to 0.72% of this theoretical requirement. However, this limited angular sampling restricted the number of fibres that could be

reconstructed. Increasing the number of projections to 18 would likely enable a complete reconstruction (Supplementary Fig. 5a), while still representing only 1.4% of the theoretical minimum.

An alternative interpretation is that the effective resolution of TomoSAXS is better characterised by the spacing between reconstructed fibres rather than the beam diameter. For the rat IVD, this spacing is approximately 8 µm. Using this value in the Crowther criterion yields a theoretical requirement of 3,142 projections. Under this assumption, the 18 projections required for a full TomoSAXS reconstruction correspond to only 0.57% of the theoretical minimum.

A similar analysis can be performed for human IVD experiment. For a 4 cm diameter sample with a 100 µm SAXS beam, an equivalent SAXS-TT experiment would require 2,513 angular projections. The 24 projections used in the TomoSAXS experiment therefore represent 0.96% of this value. If instead the spacing between traced fibres (~75 µm) is taken as the relevant resolution, the theoretical requirement increases to 5,026 projections, meaning that only 0.48% of the theoretical minimum is required for TomoSAXS reconstruction.

While there are also radiography projections in our technique to get the 3D CT image (which are not required in SAXS-TT), these are full field, relatively low dose per projection and very fast taking little time in comparison to SAXS acquisitions.

1.11 - The novelty of the here presented TomoSAXS is to get SAXS information on a single fibre level, rather than for an average as a voxel size in SAXS tensor tomography. I would argue however that the main advantage is to do that with less dose, one could imagine that one can also deconvolve multiple fibre contributions within one voxel of a SAXS tensor tomography reconstruction and with that end with the same information. That could even help lifting some limitations of needing enough sparse beam paths to get the iterative indexing of fibers going. It would be interesting to compare the radiation dose to demonstrate the advantage of TomoSAXS in that respect.

We thank the reviewers for this comment, and have now explicitly stated the dose advantage of *TomoSAXS* compared to SAXS-TT:

Line 362: “The total radiation dose applied during *in situ* experiments was 70.8 kGy (7.3 kGy per SAXS, 26.6 kGy per pCT), compared to ~2000 kGy that would be required to perform SAXS-TT measurements using the same set-up. ”

For the second part of the Reviewer’s point (comparing SAXS-TT and *TomoSAXS*), we have responded in more detail in the later point (**1.17**). We agree that a combination of SAXS-TT to estimate orientation followed by *TomoSAXS* to measure the strain patterns would have huge potential. We believe the CT-SAXS correlation and inherent link to microstructure is another key advantage,

For the radiation damage comment, please also see our response to Reviewer 4 in point **4.7**.

1.12 - Figure 2 ciii): The double fibre fit seems straight forward if the overlapping peaks in the azimuthal direction are separated in the radial direction but what happens if it's not the case? The two apparently very different scenarios are not discussed in Methods-Module 4.

This point was not explained clearly in the original MS. In brief, the double fibre fit is carried out when, over a specified *angular* range across χ (not radial across q), the dominant intensity comes from two fibres which are i) angularly close but ii) still distinctly separated in the χ- direction. No assumption of peak separation in radial direction is made. Across this specified angular range, multiple (3) radial slices are made, which will contain contributions from both fibre 1 and 2, and a joint fit is carried out. The combination of i) and ii) is to avoid degenerate cases where the fibres have their angular peak too close together (contra case ii)) so that radial slices will not be able to deconvolute the two peaks (this is the situation referred to as "not the case" by the Reviewers). Angular separation enables resolution of individual fibril parameters even if the peaks are close together in radial direction.

Changes made in text:

We have expanded the explanation of the multiple fibre fitting (See response to **R1.6**). Following the specific point raised in **R1.12**, we have added the following text:

Line 571: *"For double and triple fibre fits, to avoid degenerate cases where multiple fibres are pointing in the same direction and with similar fibril parameters, we enforce a minimum separation of 5° between fibrils, as well as minimum predicted intensity levels from each fibre to avoid dominance by one (for doublet) or two (for triplet) fibres."*

1.13 - On line 113 some sub-sections along q are mentioned, not sure what that is, is it 3 azimuthal profiles in the range? Isolated angularly independent scattering instances are identified with maximum 3 fibers contributing predominately, but only in a subset of the scattering point? How many fibers can then be in the beam path that this criteria is fulfilled?

We have removed this line from the main manuscript, in line with improving readability for a broad scientific audience. We have clarified this point in the Methods (see response to point **R1.6** and **R1.12** above).

1.14 - Extended data figure 2: as already mentioned as example of "too much information" consider to reduce, or structure better indicating subfigures with SAXS parameters and DVC parameters. Curvature is reported, but how is that calculated? It seems unnecessary to use different color scales for all the parameters in particular since some are still very similar.

Thank you for your comments. We have grouped the parameters shown in Extended Data Fig. 5 (previously 2) into pCT and SAXS derived results. We apologise for the missing curvature methodology; this has now been added:

Line 484: *"Fibre curvature ($k(s)$) was evaluated from the first and second derivatives of the fitted curve using the standard 3D definition:*

$$k(s) = \frac{\|\boldsymbol{r}'(s) \times \boldsymbol{r}''(s)\|}{\|\boldsymbol{r}'(s)\|^3}$$

Each plot in Extended Data Fig. 5 (previously 2) shows a different parameter, with different parameters requiring linear, diverging, or circular colourmaps. We decided it would be less

confusing to give each parameter a different colourmap than to arbitrarily give some parameters shared colourmaps. All colourmaps presented are perceptually uniform and colour-blind friendly (Crameri et al., 2020).

Validation of the method:
1.15 - Comparison with 2D SAXS (ext. data. Fig 6, S.I Note 19): As mentioned the key points are that there is less averaging/overlapping and thus the fitting is better, the figure is complicated to convey this. Some details to the figure:
align x-axes between (ai) and (aiii), Legends missing in (bii) and (biii)
Not sure about (bii) and (biii), why is the combined peak position higher than the 3 individual simulated fibres in (bii), and it is unclear what (biii) is.
Check if the caption is correct: (bi) should be (bii) and (bii) should be (biii) ?
One could assume it all depends on the number of simulated fibres vs number of frames?

ED Figure 9 (formerly Figure 6) has been revised in line with the comments above. The new figure and caption are presented below and in the main MS. Only one sample is now shown for clarity. X-axes are aligned for plots in (a). Legends have been added to bii and biii. The reason why the combined peak position is higher is because here we are not reporting the angular average, but the angular integration (i.e. we are not dividing by the total angular span). So while the reviewer is quite right that a 0-180 azimuthal average would have the peak intensity lower than focused slices along the main χ-directions for intensity, a 0-180° azimuthal integration will be higher.

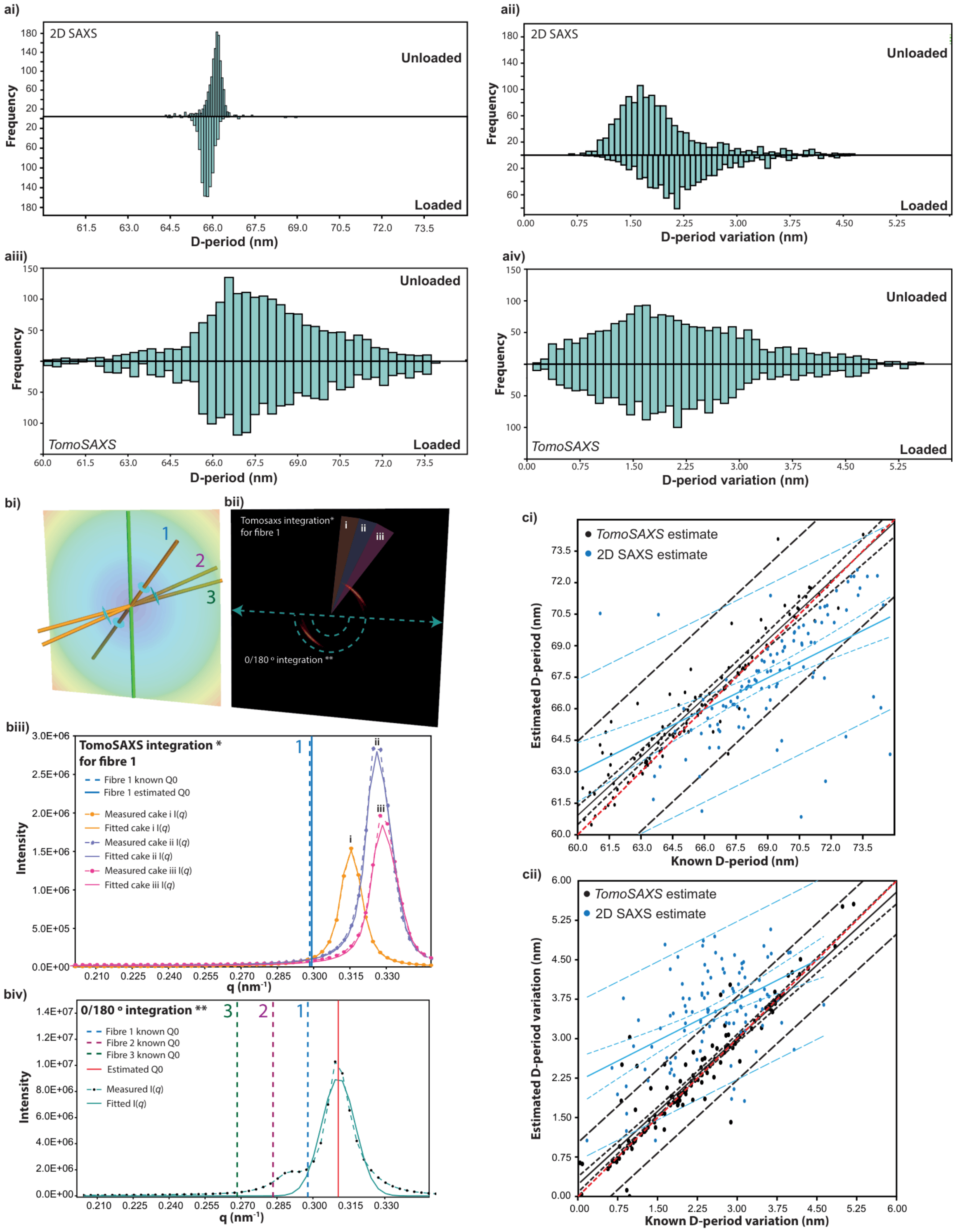

Extended Data Figure 9 – Comparison between performance using *TomoSAXS* versus traditional 2D SAXS approaches for studying nanoscale structure in IVD. a) Comparison between estimates of mean D-period and D-period variation for sample IVD1 using 2D SAXS (above) and *TomoSAXS* (below) analyses of IVD samples. b) Comparison between 2D SAXS and *TomoSAXS* analysis of single voxels modelled using 3D diffraction models, consisting of 3 fibrils of known scattering properties taken from a random distribution with the reported range from real IVD samples (bi). (bii) Comparison between *TomoSAXS* integration of independent scattering regions (coloured cakes i-iii for Fibre 1 in bi), and traditional 0-180°

2D SAXS azimuthal integration (dashed green lines). (biii) *TomoSAXS* fitting of 3D diffraction models to radial integrations across each labelled cake in (bii) based on the 3D orientation of Fibre 1, alongside known q_0 (nm^{-1}: inverse to D-period) and estimated q_0. (biv) 2D SAXS fitting of combined scatter integrated from 0-180° across χ using a Gaussian model, alongside a single estimate of q_0 using this model and known values for each of the three fibres in the voxel. c) Comparison between known and fitted D-period (ci) and D-period variation (cii) for 100 simulated fibres using *TomoSAXS* (black) and 2D SAXS (blue).

1.16 - Phantom comparison (ext. data. Fig 7, S.I note 18): This is very valuable comparison to check the reconstruction of the SAXS parameters, and the results are convincing. Maybe the precision could be reported as relative values to the variation between the fibres, rather than absolute. This does not validate the phase-contrast fibre identification part though.

This is an interesting point. We reported accuracy and precision in nm for consistency with the DVC results. We now present our accuracy and precision estimates as relative to the 6σ width of the distributions of each parameter:

Line 162: "*Accuracy, measured as mean absolute error (MAER, Methods), was 1.032 nm for mean D-period (6.8 % of the 6σ width), 0.280 nm for D-period variation (4.2 % of the 6σ width), and 8.532 nm for fibril diameter (6.9 % of the 6σ width). Precision, measured as standard deviation of absolute error (SDER, Methods), was 1.37 nm for mean D-period (9.05 % of the 6σ width), 0.34 nm for D-period variation (5.1 % of the 6σ width), and 15.32 nm for fibril diameter (6.9 % of the 6σ width).*"

In relation to their phase contrast fibre identification comment, we have added and additional extended data figure to show the results of the fibre tracing versus the greyscale images in different regions of the AF to provide further validation of the fibre tracing results. See the new Supplementary Figure 2, which we reproduce below. The code used for fibre reconstruction (Avizo XFiber) is a commercially available package which has been used widely in other IVD studies(Disney et al., 2019, 2022, 2026).

Following this comment, and that of **R4.3**, we have also added a new phantom reconstruction plot for the same geometries but with zero experimental noise, to check that the correlation improves. This is described in our response to **R4.3**.

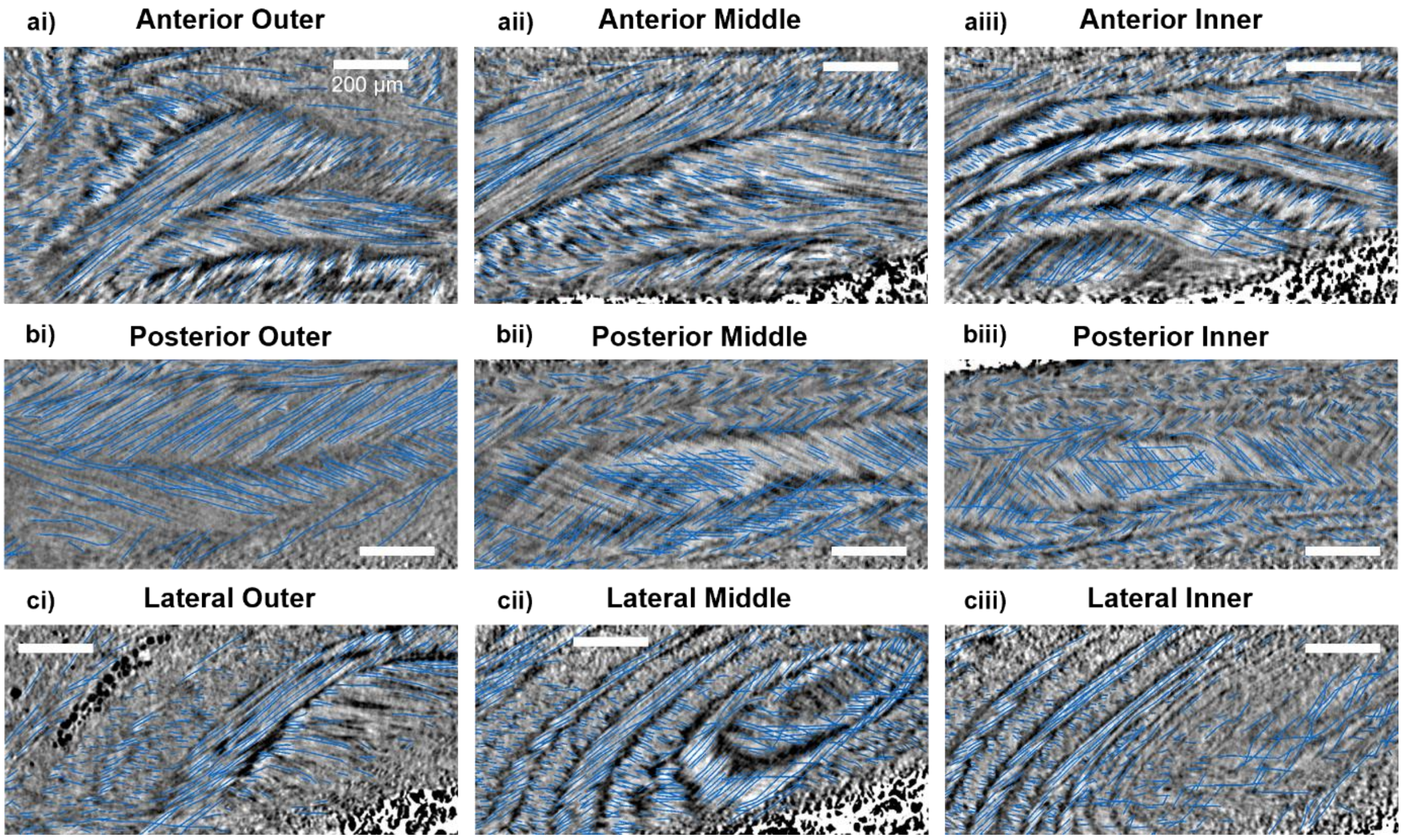

Supplementary Figure 2: Fibre tracing results (blue) overlayed on greyscale pCT images of the IVD in a) the anterior, b) the posterior, and c) the lateral i) inner, ii) middle, and iii) outer regions of the annulus fibrosus.

1.17 - Comparison with SASTT (ext. data 8, S.I note 20):
The 3D RSM is simulated for 3 fibres, and a SASTT reconstruction is simulated using a q-resolved spherical harmonics reconstruction. Then, how is the SASTT RSM analysed in (aiii) ? One gaussian is fitted to the spherically averaged intensity? In that case, this would be the same validation as in the 2D case. What about directly fitting the intensities in the individual fibre directions within a voxel, that could also be used to then further deconvolve single-fiber contributions?

We agree that the SAXS-TT vs *TomoSAXS* comparison was confusing in the earlier version, as two aspects were being shown together:

1) How fibril scattering shape biases estimates of D-period using conventional methods.

2) The averaging effect for voxels with multiple fibres estimated with SAXS-TT vs *TomoSAXS*.

In the revised figure, we separate these two effects (**ED Fig. 10a and b,** see below), respectively, and explore how both SAXS-TT and *TomoSAXS* might deal with these issues.

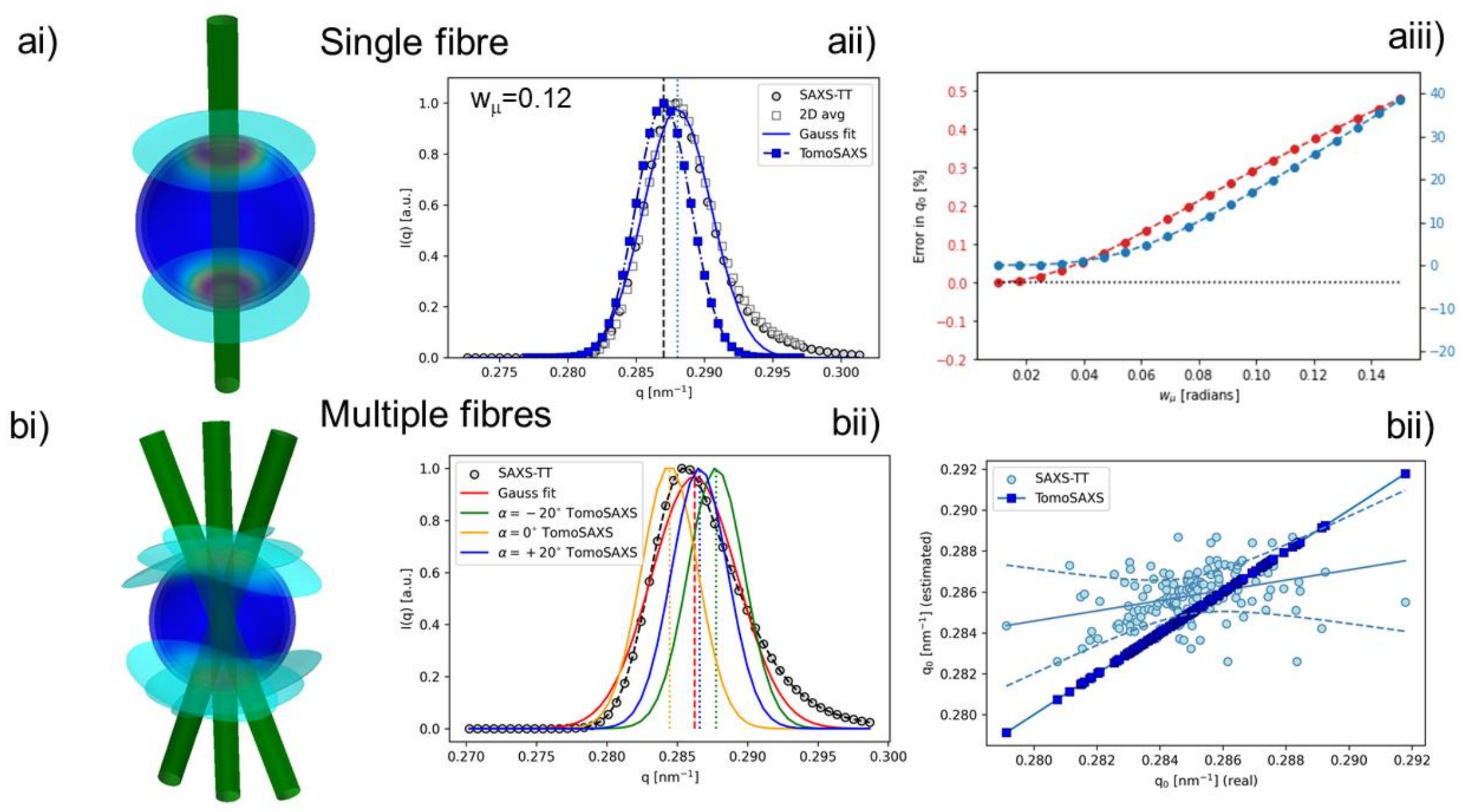

Extended Data Figure 10 – *Comparison between SAXS tensor tomography (SAXS-TT) and TomoSAXS for reconstructing scattering parameters of individual fibres.* *aii) 3D rendering of reciprocal space intensity for a single vertically oriented fibril showing the meridional peak, overlaid with three translucent reciprocal space spherical intensity maps closely spaced around the peak position. aii) I(q) plots for the SAXS-TT reconstruction using the s=0 harmonic (circles), 2D averages (squares) and a narrow profile along the vertical (dash-dot), which gives the true value and can be estimated from the TomoSAXS fit. Blue: Gaussian fit to the SAXS-TT reconstruction. Dashed vertical line: true q_0 value; dotted blue vertical line: estimated from Gaussian fit. aiii) % error in q_0 and w_a when estimated using the Gaussian fit, for varying values of w_μ (inversely related to fibril radius), showing progressive deviation at higher w_μ. bi) 3D rendering of the reciprocal space intensity and spherical intensity maps, for 3 fibres separated angularly by 20° in the same plane and in the same voxel. bii) SAXS-TT reconstruction of the combined scattering (circles/dashed line) and Gaussian fit to SAXS-TT profile (red line). Green, orange and blue lines denote narrow angular profiles along the centre of each fibre orientation, as estimated with TomoSAXS, giving the true q_0 values (vertical dotted lines, same colour code). biii) Comparison of TomoSAXS-reconstructed data (dotted line) versus values calculated by SAXS-TT for a range of fibril parameters; since the SAXS-TT I(q) calculates an average scattering characteristic of fibres in the voxel, there is less sensitivity to inter-fibre variation.*

Error in D-period and strain heterogeneity

In the new Extended Data Figure 10ai, we show the meridional scattering by a single fibril in reciprocal space. Due to the pancake-shaped intensity pattern, the reciprocal space map (RSM) – when represented as spherical shells – will show spherical sectors for $q<q_0 + d(q_0, w_a)$, and rings for larger q. As per the published SAXS-TT protocols (Pierantoni and Silva Barreto et al., 2023), the I(q) curve is obtained by reconstructing the intensity at each shell using the zeroth order spherical harmonic (s_{00}) which is a constant. We have reproduced this by doing a 2D fit in (q,f) at each RSM sphere, and when concatenated, leads to the curve shown in Extended Data Figure 10aii.

(As the reviewer correctly notes, this provides the same result as for 2D averaging due to spherical symmetry, and the 2D curve is overlaid on the SAXS-TT I(q) reconstruction).

To estimate the D-period and fibril strain heterogeneity, a Gaussian model is fitted to the data. Due to the finite w_μ, there is a rightward skew in the peak (explored in (Badar et al., 2022, 2025)), and as a result: i) the D-period is underestimated and ii) the strain heterogeneity is overestimated, versus their true values.

In *TomoSAXS*, the D-period and strain heterogeneity are obtained by fitting the diffraction model to a narrow angular range in the vertical sector, giving the correct parameters q_0 and w_a.

In Extended Data Figure 10aiii, we show the % error in estimated D-period and w_a arising when the SAXS-TT concentric shell method is used. For large fibril radii (small w_μ), the error is small, but increases as the fibril radii decreases, due to the increasing equatorial width. For smaller-diameter collagen fibrils (e.g. 40-60 nm as in Type-II) this error is noticeable and of the same order as the average shifts in D-period seen in our data. It is likely that type-I collagen fibrils (with diameters >100 nm) will lead to lower errors.

Multiple fibre effect

In Extended Data Figure 10b, the estimation of D-period on per-voxel (SAXS-TT) and per-fibre (*TomoSAXS*) is compared for three fibres in a SAXS voxel with fixed angular positions (α = --20°, 0 °, 20 °; β = 90 °) and variable q_0.

For SAXS-TT, the RSM is evaluated across a q-range around their mean value, and a resultant *I(q)* and Gaussian peak fit is obtained as in (a). A single D-period value is obtained for each voxel (i.e. the same value is given to each fibre within a voxel).

For *TomoSAXS*, due to the angular separation of the peaks for the different fibres, narrow angular averages for each fibre can be carried out, resulting in recovery of the individual parameters for each fibre.

The per-voxel nature of SAXS-TT leads to, in this case, a lowered sensitivity to the true D-period values as seen in Extended Data Figure 10biii, where a range of different q_0 positions for the fibres were simulated. The resulting linear correlation curve shows a reduced slope <1.

As a final comparison of the two methods, we note that fibres along the same direction which have clearly different q_0 values could be resolved in SAXS-TT if their q-separation was large enough to distinguish the peaks in the raw data, though we are not aware of SAXS-TT studies on experimental systems which do this. While *TomoSAXS* does not do this yet, it could be similarly extended to analyse overlapping peaks where clearly radially separated peaks are visible in the data, extending the angular separation concept used above (**R1.6**, **R1.12** and **R1.13**).

In summary this is an impressive piece of work for which we would like to congratulate the authors. With a clear description of limitations and by presenting the key messages more clearly, we recommend publication.
This review has been performed as co-review.

We are very grateful to the Reviewers for their insightful, constructive and encouraging comments.

Referee #3 (Remarks to the Author):

This paper describes the use of TomoSAXS to map fibril to fibre mechanics across intervertebral discs. A large amount of work is presented for which the authors should be commended. The results are novel and would be of interest to any researcher interested in the fundamental biomechanics of intervertebral discs. The insight into the interaction between structures across scales in unique. I have several points that should be addressed before publication:

3.1. Line 15 – A reference to support the claim that injury and age related degeneration are key drivers for backpain. I think the relationship is more complicated than is suggested.

Thank you for the comment. We agree that the relationship between disc degeneration and back pain is complicated, and we have added reference 11 (Lyu et al., 2021) to line 16 to better explore/ explain this:

Lyu, F.-J. et al. Painful intervertebral disc degeneration and inflammation: from laboratory evidence to clinical interventions. Bone Res. ***9****, 7 (2021).*

3.2. Line 22 – I think you should say here that you are talking about both animal and human IVDs.

Thank you for this comment, we have modified lines 24-26 to include both rat and human IVDs:

Line 24: "*We apply this technique to intact rat IVDs under physiologically relevant loading to investigate structure–function relationships across multiple length scales. We then extend the technique to intact human IVDs in static conditions*."

3.3. Figure 1 – small comments but the ant-post arrow on ai could better align with the anterior and posterior regions of the disc shown. At the moment the arrow looks more cranial-caudal.

Thank you for this comment, we have adjusted the ant-post arrow in Figure 1 to better align with the disc.

3.4. Line 154 – It should be stated that ref 24 is in human discs, not rat. Since they are not both rat, should we expect the strains to be similar? Also DVC errors are usually largest at the endplates. Does this mean you can't trust your result that the strains were higher in this region? Later, you explain your zero-strain study, but you don't give any indication that errors are different in different regions of the discs, which they will be, especially close to the endplates.

This is a good point, and we agree that care should be taken when interpreting DVC data from the noisiest parts of the images, which, as you say, tend to be close to the endplates. We do not see this difference in strain uncertainty from either the zero-strain or virtual compression studies because the position of imaging artefacts is not changing between successive images, which they will in a real *in situ* loading experiment. In light of this, we

have removed our statement about high strains measured near the endplates, as it is not central to the study and could be affected by DVC errors.

In terms of regional variation in DVC accuracy, we have not quantified this for this study as we do not perform any kind of regional comparison within the disc which could be affected by differences in DVC uncertainty. Looking at the map of DVC uncertainty shown below, there are regions that have higher uncertainties than others, particularly isolated fibres, points surrounding gaps in the fibre tracing due to imaging artefacts, and one posterior-lateral region. The strain patterns discussed in the manuscript (radial strain bridges and lamellar texture) can be seen in all regions of the AF (see Extended Data Fig. 5), so regional variations in DVC accuracy should not have an impact on these results.

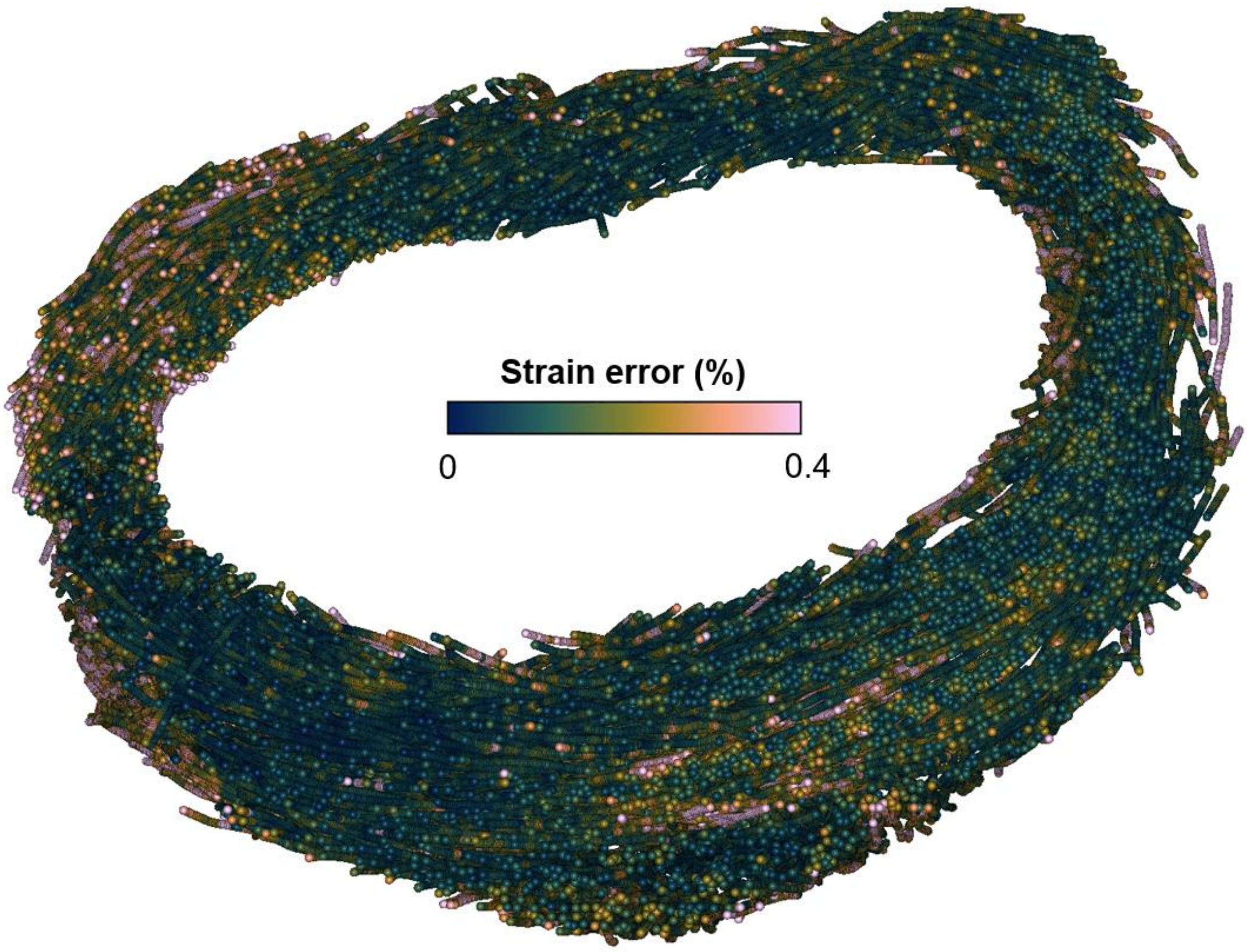

Response Figure 1 – DVC strain error distribution from zero-strain analysis.

3.5. Line 169 – has it previously been shown that fluid flow from NP into AF is seen in this sort of loading and that it creates swelling? Is there any literature you can cite to back up this idea?

To our knowledge, prior studies have not directly visualised fluid moving from the nucleus pulposus (NP) into individual annular lamellae during stress relaxation. However, the broader mechanism is well supported: the intervertebral disc behaves as a biphasic/swelling tissue, in which externally applied compression is balanced by fluid pressurisation and

osmotic swelling, and fluid redistribution is central to the time-dependent response. Human disc creep/recovery experiments explicitly identify fluid flow as a key driver of hydration and height changes under axial loading and unloading, while tissue-level measurements show that both NP and AF possess measurable swelling properties (Yang & O'Connell, 2019). There is also evidence that these local AF strain patterns are linked to swelling-related residual stress development. Residual-strain studies have shown that the AF contains a structured residual stress/strain field that develops in response to NP pressurisation and in vivo loading, with radial tension present in some inner-AF regions (Dusfour et al., 2021). In addition, swelling-based finite-element analyses have shown that disc swelling capacity strongly influences intradiscal deformations and helps maintain strain homeostasis throughout the AF (Yang & O'Connell, 2019). We have added the following reference to line 207:

Yang, B. & O'Connell, G. D. Intervertebral disc swelling maintains strain homeostasis throughout the annulus fibrosus: A finite element analysis of healthy and degenerated discs. *Acta Biomater.* **100**, 61–74 (2019).

3.6. Line 171 and 172 – Standard deviations should be added to these average directions.

Thank you for this comment, we have added standard deviations to lines 209-210.

3.7. Line 171 – Can you explain in more detail how the two angles for compression and tension indicate substantial strain accommodation by the extrafibrillar matrix and inter-fibre motion. I think readers would appreciate this being explained more clearly.

Thank you for this comment. We have added a clearer explanation to the manuscript:

Line 210: "*Fibres resist tensile strains aligned with their orientation but provide little resistance to strain in other directions. The lack of alignment between tissue-level principal strains and fibre orientation therefore indicates substantial strain accommodation by the extrafibrillar matrix and inter-fibre motion.*"

3.8. Line 200 – It would be useful to compare these stresses to the stresses you would expect failure. Have these failure stresses been previously quantified? Are these stresses close to failure, or a long way off?

Thank you for this comment, interestingly, it would seem these stresses are close to failure. We hypothesise that this may be one of the reasons why the AF is so susceptible to microdamage under sub-failure repeated loading of the IVD. We have added discussion of this to the manuscript:

Line 241: "*The measured failure stress of single collagen fibrils from rat tendons range from 70-200 MPa(Andriotis et al., 2023), indicating that residual stress in the IVD may result in fibrillar strains close to failure. This may help explain why the annulus fibrosus is susceptible to microdamage at mechanical loads well below those required to induce failure in the disc(Schollum et al., 2018).*"

3.9. Line 362 – I expect immersing these specimens in PBS would result in over-hydration. How did you prevent overhydration? Was the hydration level of discs physiological at the point of scanning?

For this experiment, immersion in PBS was the best way to maintain a constant hydration environment, with axial compression used to prevent over swelling of the NP. Progressive swelling was not seen in the measured force or imaging datasets.

We have been careful to use term ‘physiologically relevant’ rather than ‘physiological’ throughout the manuscript. The use of fresh-frozen, hydrated, unstained intact IVD for the *in situ* experimentation is a major advantage of the technique over other experimental methodologies probing the nanoscale. However, an ideal experiment would utilise a humidity and temperature-controlled chamber, however designing one compatible with the synchrotron scanning and in situ loading protocols used here was outside the scope of this project. We have stated this limitation in the discussion:

Line 368: “*Whilst efforts were made to perform physiologically relevant in situ IVD mechanical experiments, truly physiological conditions would require the use of a humidity and temperature-controlled sample environment and multi-axis mechanical testing rig compatible with synchrotron beamlines*.”

3.10. Line 400 – could you add justification as to why you chose to axially compress specimen, rather than axially rotate or flex?

Axial compression was chosen due to ease of experimental set-up and to maintain sample alignment with the X-ray beam post-loading. The application of a homogenous displacement across the entire sample meant it was easier to control the maximum displacement for DVC measurement and how much of the sample stayed within SAXS beam paths. The loading rigs available for this experiment cannot perform axial rotation, flexion would have been possible but difficult to set-up due to the small size of the samples.

We have added justification for axial compression to the methods:

Line 464: “*Axial compression was chosen for ease of experimentation and interpretation of results.*”

We hope to explore other loading mechanisms in future experiments.

3.11. Line 406 – These calculations to convert an applied load to an internal pressure would be more trustworthy if the fact that NP pressure differs from AF pressure. So this simple conversion is not an accurate method to compare to pressure measured in-vivo (in the NP region).

We thank the reviewer for this comment and agree that the calculation was approximate and not necessarily representative of IVD pressure. We have removed this calculation from the manuscript and instead stated that the preload was designed “*to prevent over-swelling of the nucleus pulposus*” in line 453.

3.12. Line 408 – How did you know that 20 minutes stress relaxation was sufficient? Justification required.

This was determined by the time taken for the force curve on the mechanical testing rig to plateau and was the length of time after which images could be reliably taken without motion artefacts.

We have justified this in the methods:

Line 454: "*Samples were allowed 20 minutes stress relaxation before scanning to minimise motion, determined as the time taken for load readings to stabilise*"

3.13. Line 409 – can you give more detail on how the samples were clamped into position. How did you make sure you did actually hold the same position? I would expect this is challenging.

This was an extremely challenging part of the *in situ* experiment. 3D printed sample holders were designed to hold the samples securely in place when removing and remounting in the mechanical testing rigs. However, the mechanism was not always successful, and multiple samples were discarded due to failed clamping. To ensure samples maintained the same position following remounting, radiographs from I13 were compared to WAXS intensity maps (collected simultaneously with SAXS data at DLS beamline I22 and providing higher optical contrast in raster maps than sum SAXS scattering data) at the beamline. We have added additional analysis of this, by comparing a WAXD diffraction scan map to the CT radiograph to support the fact that samples maintained their positioning between scans, which is shown in the Supplementary Figure above. The linear correlation between WAXD and CT reference points shows a strong linear correlation which, while not perfectly 1, has a displacement error of 2-3 µm, well within the pCT voxel sizes used for spatial registration (8.125 µm width) SAXS beam diameter size of 20 µm.

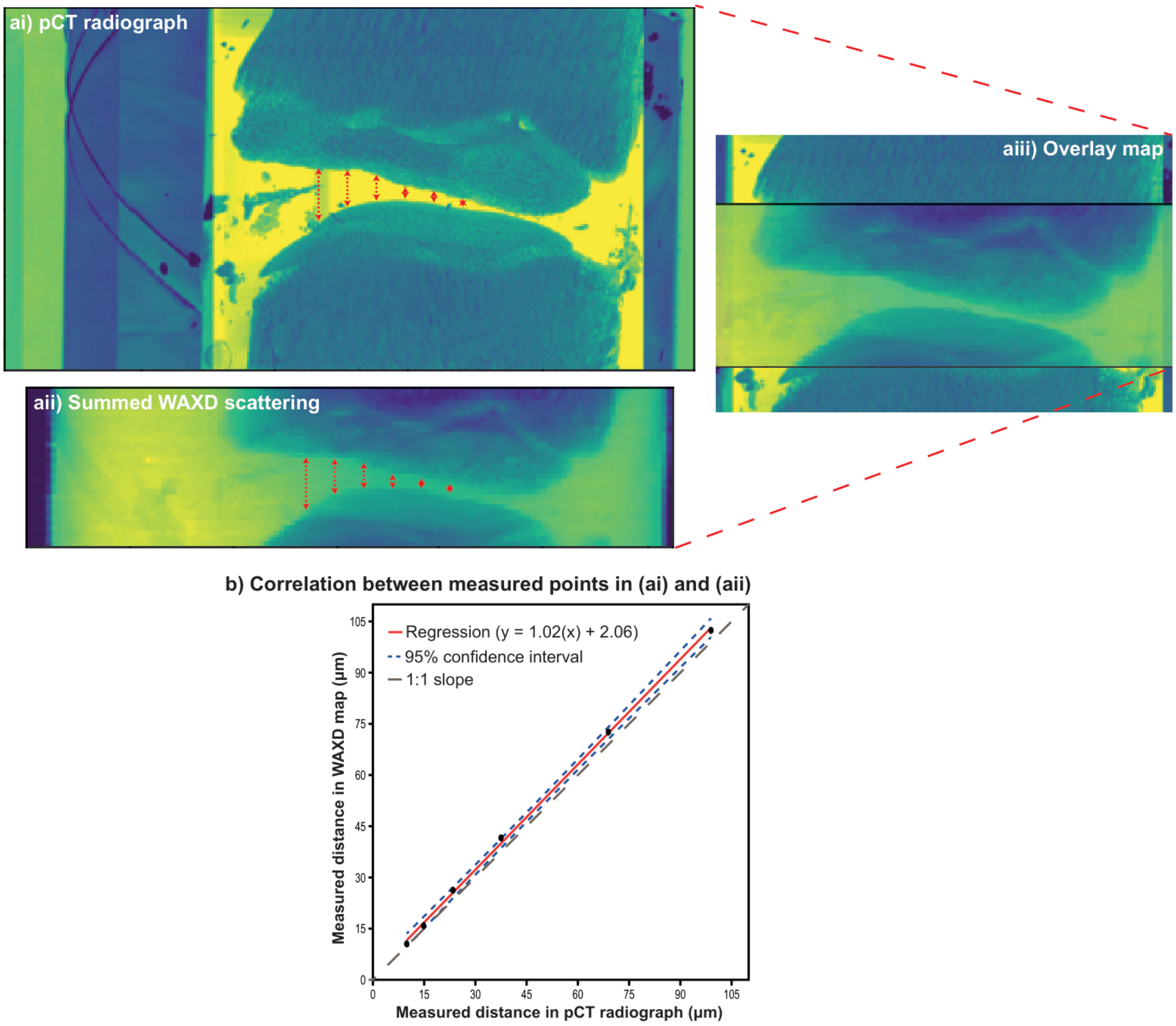

Supplementary Figure 4 – Spatial correlation between resliced pCT data (ai) and mapped total wide angle X-ray diffraction (WAXD) scattering (aii) for the equivalent angular orientation of the *TomoSAXS* tomography (used for vertical calibration between datasets: overlay plot between images in aiii). Dashed arrows in (a) highlight location of measurement points between vertebral end-plates in both modalities. These are regressed in (b), with measurement error consistently within the absolute voxel size of fibre tracing voxels (8.125 µm width) and tomoSAXS beam paths (20 µm width).

A description of clamping has been added to line 455:

"*samples were clamped in position using bespoke 3D-printed sample holders and screws (Supplementary Fig. 1)"*

We have added a new Supplementary Figure 4 showing correlation between WAXD and CT scans for alignment (see above).

3.14. Line 413 - what was your definition of just touching? it is usual to use a load for this threshold to ensure this step is repeatable.

Thank you for this comment, we have redefined this in the manuscript :

Line 459: "*The bottom platen of the rig was slowly raised until screw hole alignment was reached between the rig and the clamped sample holder, which was then secured in place*."

Screw hole alignment was used to determine when the sample was correctly mounted. We did not use a load threshold as it was vital no additional load was applied to the samples during this step.

3.15. Line 415 – how was the 50 micron displacement applied? This is not explained in enough detail. Was this loading purely axial? How did you make sure? Was the mid-transverse slice of the IVD parallel to the loading plates? How did you make sure of this? If you couldn't, how would any errors in this affect your results?

We have added detail of the displacement application to the methods:

Line 462: "*a 50-micron displacement was applied to the bottom platen of the sample holder in the laboratory z-direction*"

And added description of sample alignment :

Line 399: "*with sample alignment designed so that the mid-transverse slice of the IVD was parallel to the loading plates*."

Sample alignment was particularly challenging for this experiment due to the small size of samples and dissection methods available. The sample alignment we aimed for was that the mid-transverse slice of the IVD was parallel to the loading plates, however, we were unable to be as precise as we would have liked to be in this process as we can see in the CT images. Because of this, we have not performed regionalised analysis of e.g. anterior vs posterior strain, as this will rely heavily on the loading axis of the disc.

Referee #4 (Remarks to the Author):

4.1 - This is a very exciting approach combining tomography and SAXS in situ, where the novelty is the presentation of a data handling approach named TomoSAXS, enabling mapping fiber to fibril mechanics within the tissue. The manuscript is very well written and clearly outlined.

It is clear that TomoSAXS as a reconstruction approach to gain local fiber and fibril strain (micro to nano scale) has high potential, and the authors present a very thorough work. However, key aspects of validation using more controlled experiments are largely missing. Despite the IVD being a compelling tissue, it presents an extremely complex case for fundamental proof of concept of a new approach. After proper validation, it is clear that TomoSAXS may gain importance is studies of fibrous materials with 3D structure and variability.

We thank the reviewer for their overall positive assessment of our manuscript and appreciate the concern about a lack of simple validation experiments. Indeed, the development of *TomoSAXS* started with lab-based imaging of dried collagen samples. We have now included these initial, proof-of-concept experiments in the manuscript in a dedicated

validation and uncertainty section (lines 144-171, Extended Data Fig. 2). We also refer the Reviewer to the next point **4.3.**

Specific comments are presented below.

4.3 - Despite including several experiments, there seems to be a lack of basic validation of the theoretical approach, e.g. showing that TomoSAXS is valid on a known sample with known fiber and fibrillar structure and distribution. The current validation includes simulated phantom data, simulated comparisons with SASTT and comparisons with in situ 2D scattering. For the latter, the match is not very good, which is inherent as TomoSAXS aims to analyze the full 3D structure (and should not match the 2D results). Despite the data seeming reasonable, much of it is very noisy and it is not possible to understand if and how much of this is simply due to true biological variability or how much is originating from theoretical limitations of the TomoSAXS fitting.

Uncertainty assessment: A large part of the presented data is very noisy, see e.g. the correlations between fiber strain and fibrillar strain (section starting on line 184, and figure 3, and extended data figure 4). The reasons for this is unclear. Again, is it biological variability, or is it in the tomoSAXS or due to uncertainties in other parts of the pipeline? How are the uncertainties in DVC and its inherent noise contributing? Is it possible to assess the propagation of uncertainties from CT-DVC-SAXS and tomoSAXS approach?

Since the reviewer makes many important points in the above paragraphs, we first split them into separate issues and address them below. The main issues raised are:

1) **validation** on a known sample

2) **source of noise and uncertainty**: biological variability or *TomoSAXS* fitting

Validation of SAXS reconstruction on known collagen tissues:

We appreciate the Reviewer's point about validating on a known structure. Due to the limited "true" nanoscale information available on many systems, this question can be separated into two components –

a) whether *TomoSAXS* gives expected structural and in situ mechanical data at the fibrillar level on systems **with simple fibre texture but known fibril-level parameters**.
b) whether *TomoSAXS* can reconstruct spatially varying fibrillar parameters in a system **with complex fibre texture.**

For part a), we now present **a new Extended Data figure and supplement section**, showing our earlier validation studies using *TomoSAXS* (with a laboratory-based SAXS X-ray source) on uniaxially oriented collagen fascicles from chicken foot tendon. This is a stable and well-characterised system, with a known D-period of ~64-65 nm, and all fibrils oriented along the tendon axis. These experiments were carried out prior to our IVD studies, as part of a validation exercise. On this system we tested the ability of *TomoSAXS* to

i. reconstruct intrinsic fibril parameters (D-period, w_a, w_μ) in single, double and crossed fibre textures.

ii. Obtain expected changes in fibrillar D-period under application of static loads to the tendon constructs (comparing to standard in situ SAXS loading tests on tendon and modelling work)

D-period lies in expected range: For part i), our results show that, as expected, estimated D-period lies in the range of 64.5-66.5 nm, consistent with prior work on dry tendon(Fratzl et al., 1993; Masic et al., 2015). The other parameters like w_a are in expected ranges (Badar et al., 2022, 2025; Pierantoni et al., 2023).

Load-induced changes of D-period in line with expected changes: For part ii), under static load (by hanging weights on the constructs), the % changes in D-period can be used to approximately estimate fibril modulus. Plotting the increase in stress versus the measured fibril strain relative to the lowest loaded fibre gives an excellent linear relationship $\sigma = E\varepsilon_F$ with E=200 MPa and an R^2 of 0.99, in the same range as previously estimated single fibril moduli (e.g. 400-1000 MPa in (Eppell et al., 2006)). Since the tendon modulus is a combination of the load response of the fibrils and shearing in the GAG phase, it is not surprising that the values are slightly lower than single fibril measurements.

Since quite approximate methods were used to apply the stress (kitchen weights) and calculate the area (digitizing hand-drawn outlines around the shapes in the figure) too much should not be read into the fortuitously high accuracy.

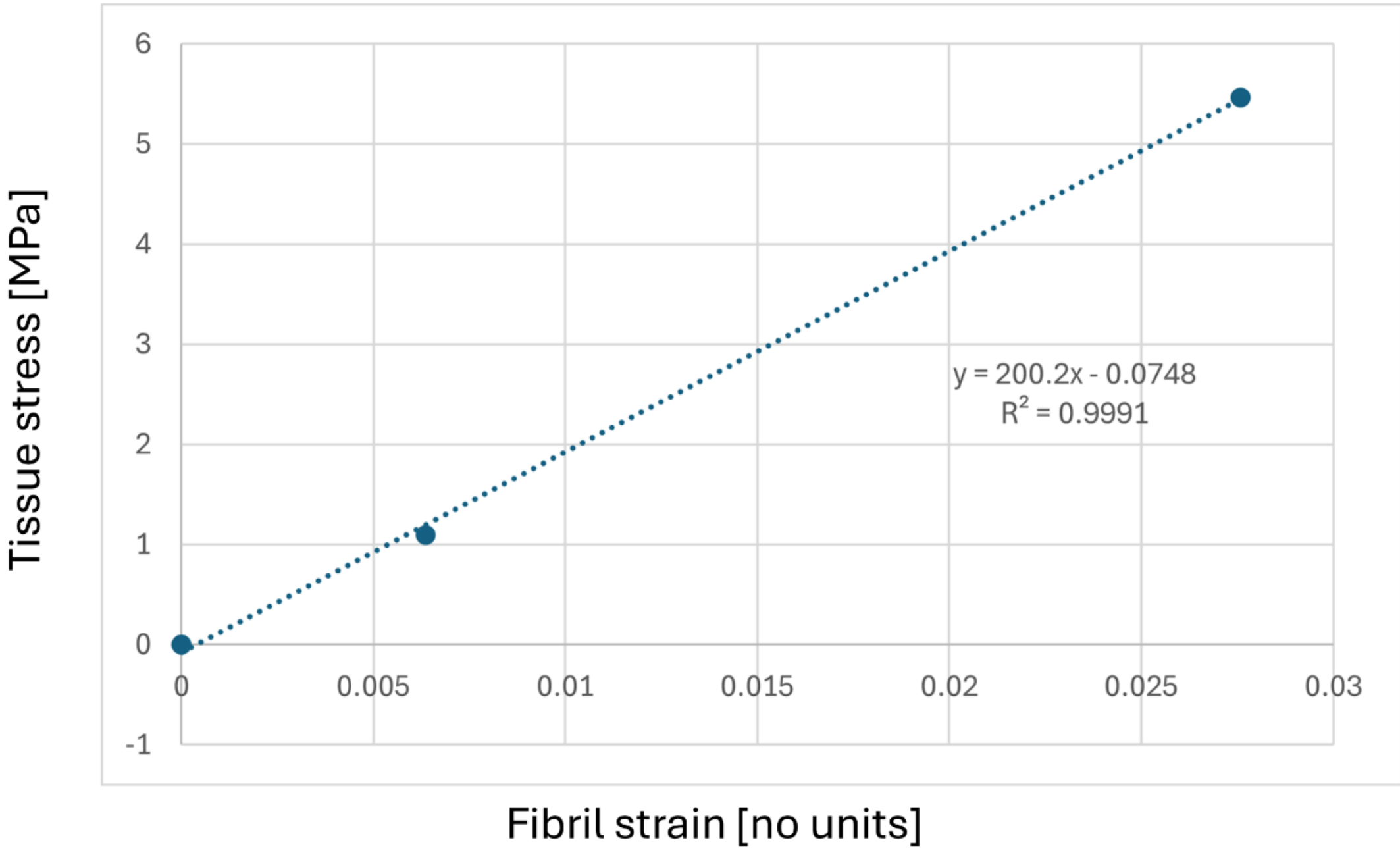

These results show that *TomoSAXS* gives the expected results for a material with simple fibre texture, known fibrillar parameters and nanoscale mechanical response.

Changes made in text:

We have created a new Extended Data Figure 2, shown below, and added text (given after the figure below).

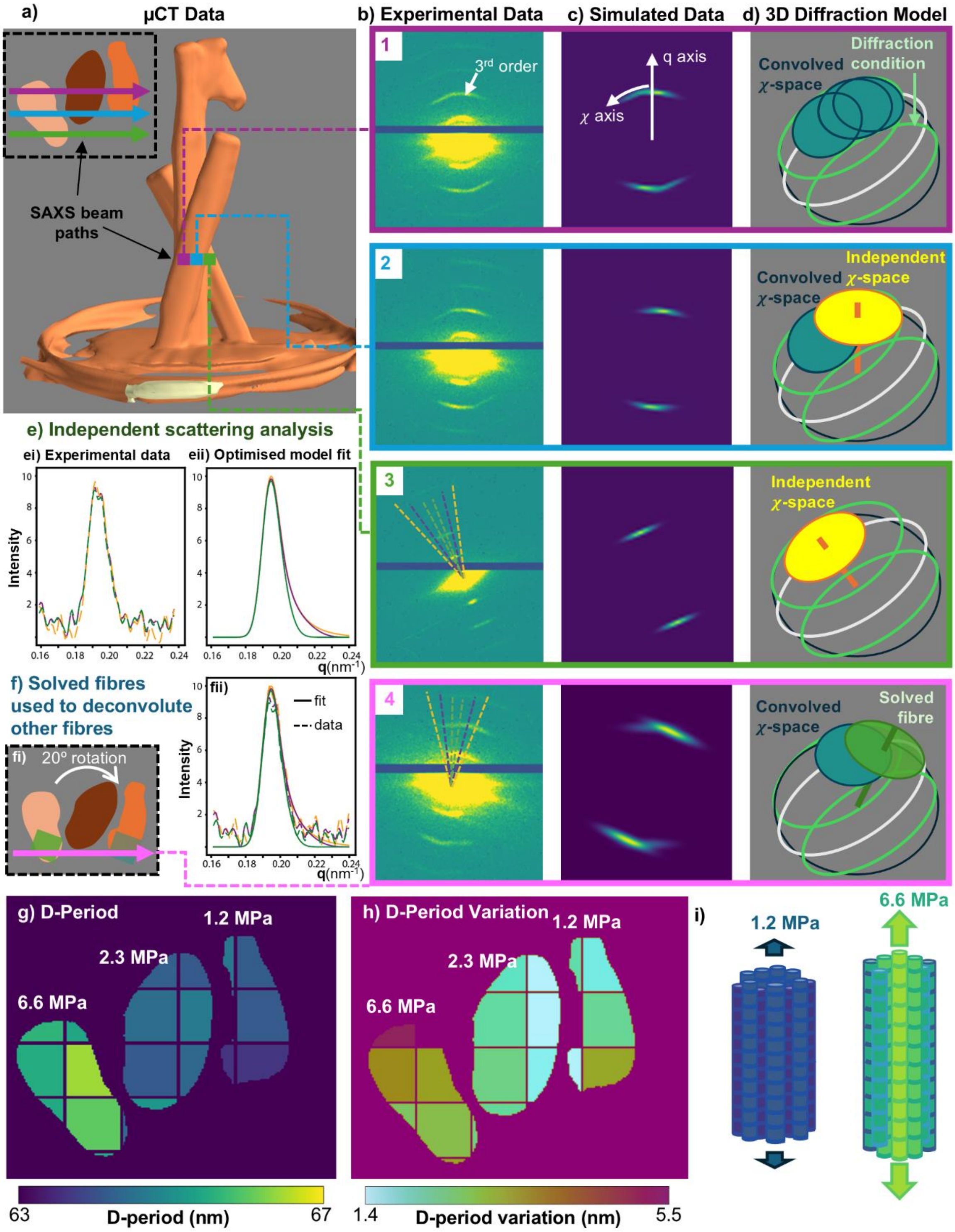

Extended Data Figure 2 - TomoSAXS validation on a simple, known system. *a) 3D rendering of laboratory microCT data from 3 dried chicken foot tendons placed at different angles, with locations of example SAXS beam paths 1, 2, and 3 shown. b) Experimental laboratory SAXS data,. c) Simulated 3rd order collagen peak SAXS data. d) 3D diffraction modelling for 4 X-ray beam paths. e) Independent scattering analysis of beam path 3; ei) experimental intensity data along the q axis; eii) optimised 3D diffraction model fit to the*

*experimental data (accounts for peak skew along q)**.** f) Results from the independent fibre in beam path 3 used to deconvolve information for the second fibre in beam path 4; fi) illustration of beam path 4 which is taken after rotating the sample 20°; fii) fit to experimental data for the convolved fibre. g) Collagen D-period and h) collagen D-period variation mapped across the 3 tendons. i) Illustration of collagen fibres in tendons dried under different tare loads - the tendon dried under 6.6 MPa tensile stress has a higher D-period and lower D-period variation compared to the tendon dried under 1.2 MPa stress.*

In ***TomoSAXS*****: Validation and uncertainty assessment** (lines 145-153)

To establish and validate the fundamental principles of the TomoSAXS reconstruction method, initial validation experiments were performed on a simple construct of dried chicken foot tendons using laboratory X-ray sources. 3 tendons were dried under different levels of tensile load to create uniformly oriented bundles of collagen fibres with different D-periods (Extended Data Fig. 2, Methods). The results showed an increased D-period and D-period variation in the tendon dried under the highest tensile strain (Extended Data Fig. 2g,h), fibril strains of 0.6-2.8% and an effective fibril modulus of ~0.2 GPa, in the same range as previous single fibril measurements, demonstrating the ability of TomoSAXS to resolve variations in nanoscale collagen properties in a known system.

In **Methods** (lines 759-772):

"***Laboratory TomoSAXS analysis of dried tendon constructs***

Chicken foot tendons were dissected and air cured under differing tare loads of 0.5 N, 1.5 N, and 3.5 N to generate different levels of pre-strain in the collagen. 3 tendons were arranged at different overlapping orientations and superglued to a 3D printed sample holder. SAXS scans were performed using a Xenocs Xeuss 3.0 (Diamond Light Source) with a beam size of 400 µm and exposure time of 5 minutes. 9 angular rotations between 0° and 180° were used, with 11 beam paths per rotation. µCT of the sample was performed using a Phoenix V|tome|x (Research Complex at Harwell) with a 14.3 µm voxel size.

The orientation of collagen fibrils within each tendon was assumed to be aligned with the principal axis of the tendon. The sample was divided into an 11 x 11 grid for TomoSAXS reconstruction rather than individual fibres, for the purpose of a simplified, proof-of-concept reconstruction."

Source of noise – biological variability vs *TomoSAXS* limitations.

For part b), when the fibre texture is more complex like in real IVDs, our digital phantom work provided evidence of the ability of *TomoSAXS* to reconstruct fibril parameters with reasonable fidelity when simulating observed experimental noise. To separate the issue of experimental noise from the reconstruction capacity of *TomoSAXS*, we have now **repeated the phantom simulations with zero noise** (same experimental geometry, random fibril parameters). We observed that the correlation between the reconstructed and ground-truth parameters increased in all cases, which gives us confidence that, for the fibres we can solve for, we are obtaining good (but not perfect) estimates.

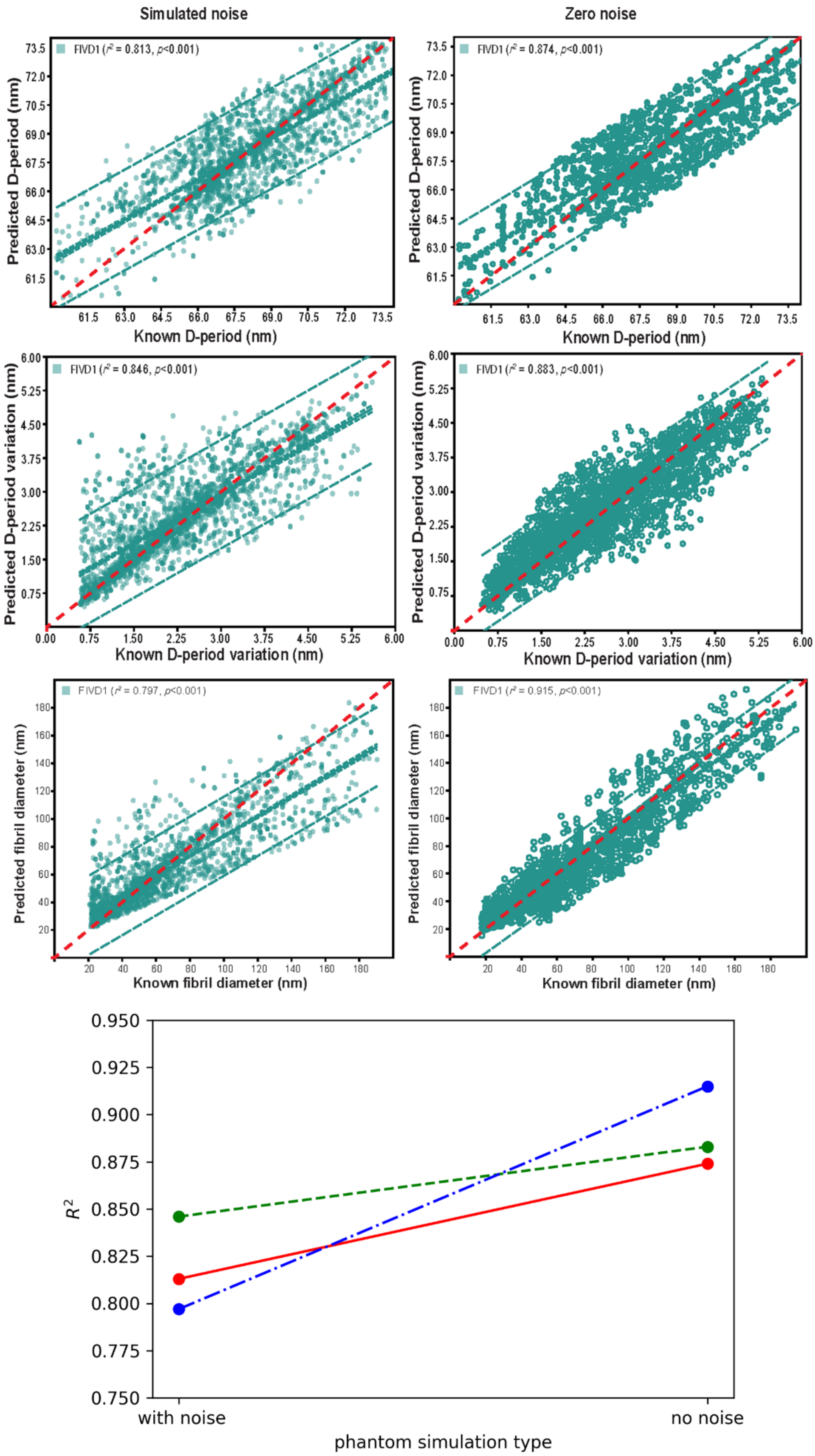

Simulated noise
Zero noise
FIVD1 (r² = 0.813, p<0.001)
FIVD1 (r² = 0.874, p<0.001)
Predicted D-period (nm)
Known D-period (nm)
FIVD1 (r² = 0.846, p<0.001)
FIVD1 (r² = 0.883, p<0.001)
Predicted D-period variation (nm)
Known D-period variation (nm)
FIVD1 (r² = 0.797, p<0.001)
FIVD1 (r² = 0.915, p<0.001)
Predicted fibril diameter (nm)
Known fibril diameter (nm)
R²
with noise
no noise
phantom simulation type

Supplementary Figure 8 - Extension of phantom simulation results in Extended Data Figure 3: Left column shows correlation plots and regression lines for sample fibre geometries with experimental noise levels, whilst right hand column corresponds to identical geometries but zero experimental noise. A consistent increase of R^2 from 0.80-0.85 to ~0.85-0.92 is observed for the noise-free case.

Changes made in text:

We have added Supplementary Figure S8 showing the improved correlation in the phantom plots with zero noise, and the text below in "***TomoSAXS*****: Validation and uncertainty assessment**" (lines 169-171), and the figure above.

"… *When provided noise-free data on identical geometries, TomoSAXS reconstruction accuracy improved, with r^2 = 0.874-0.915, which demonstrates the theoretical limitations of the technique (Supplementary Figure 8).* …"

Known biological variability of nanoscale structural parameters:

The inherent variability of SAXS-derived nanostructure parameters is well-established from much prior work, using both 2D SAXS and 3D SAXS TT approaches. At this stage, it remains an open question which we cannot answer conclusively, but (in line with this and earlier reviewer comments – see response to reviewer comments **1.16**) one approach might be to look at the average error in estimating fibril parameters from zero-noise phantom simulations and compare it to the variability observed across the specimen.

A critical potential source of error in *TomoSAXS* (as opposed to SAXS-TT) is the accuracy of the fibre angle assignment (from CT) to fibres along each SAXS beam path. The accuracy of this is addressed in the related question by R3 (**3.13**) on how accurately we could match positions, and we refer the Reviewer to that response for details.

The displacement accuracy measured from zero-strain analysis was 88 nm, and 198 nm for a virtual compression of 10 %. These values are significantly smaller than the beam size for SAXS (20 microns), so are unlikely to have contributed significantly to uncertainties in the SAXS measurements.

4.5 - The main experiments are performed on rat IVDs in situ. Then, a human experiment is included as preliminary data of TomoSAXS, but in my understanding it is a static combined tomography and SAXS experiment? Thus it is not including in situ loading? (line 257 etc) It is also a formalin fixed specimen (line 691). The role of this part in the paper is unclear. In my opinion, nature papers should not include preliminary findings (It should either be in and then fully analyzed to align with the aims of the study, or it should be removed). Currently the relevance without in situ and of fixed specimens are unclear.

This is an important point. We would contend that *TomoSAXS* is, first and foremost, about correlating 3D SAXS (nanoscale) volumetric measurements with 3D CT (microscale) structure. Static *TomoSAXS* (no application of *in situ* stimulus) is a very valuable technique to understand e.g. multiscale adaptation and degeneration in musculoskeletal tissues. The application of load in situ is one application (used here) but so are other stimuli such as heating, corrosion, pathology and ageing etc which can induce structural changes across

scales. Hence, the absence of in situ loading is not, in our view, a critical gap for the presentation of the human IVD data. **We have transformed the human feasibility into a full TomoSAXS reconstruction of fibre D-spacing for one slice of a human thoracic IVD**, demonstrating the applicability of TomoSAXS to helping solve biological questions in human organs

We believe the combination of chicken tendon validation, IVD reconstruction in rat IVDs under load, and the reconstruction of a single slice of human IVD demonstrates the validity of the technique in 1) accurately measuring the fibrillar parameters, 2) correlating to microstructural deformation, and 3) showing translational potential to large-scale human tissue, to fully demonstrate the potential of the method to help address human biological questions.

Based on the above, the primary issue remaining for performing *TomoSAXS* on structures like human IVD (a 10^3 increase in volume vs the rat IVD) is the technical challenge of reconstructing beam paths with a concurrently higher density of individual collagen fibres. We have here made progress in overcoming this issue through adapting three main processing components to our approach (importantly without modifying the inherent logic and per-fibre reconstruction method from our rat IVD experiment). These are:

1. Angular clustering to coarse-grain adjacent fibres with the same orientation into one indexed region.
2. Account for multiple fibre overlap by increasing overlap fits from 1-3 to 1-5 fibres.
3. Parallelising processing of individual rotational steps, rather than sequential solution.

A detailed description of this modification is given in Supplementary Note 22.

These modifications to our reconstruction program have allowed 68% of fibres in a *TomoSAXS* slice of human IVD data to be fitted over three runs of our reconstruction pipeline (new **Fig. 4**). This demonstrates that *TomoSAXS* can reconstruct large samples of soft collagen tissues including human IVD, albeit at the expense of some spatial resolution for closely aligned and adjacent fibres in the current implementation. Even without applying or analysing biomechanics, spatial assessment of clinically relevant micro and nanostructure elements across entire human tissues is now possible. Further, because of the nature of *TomoSAXS* (combining CT orientation with SAXS nanostructure), large increases in sample size do not require increases in the number of angular SAXS projections – making it highly scalable. See response **1.10** and Supplementary Figure 5.a.

We also understand R4's concerns about mechanical property change in formalin fixed tissues. However, given that most human organ tissues in biobanks will be formalin fixed or otherwise preserved, establishing what we can learn from such tissues is an important future area to explore, to apply *TomoSAXS* to clinically relevant questions.

4.6 - Scan time is presented as a limitation, but the total time for the testing of one rat IVD is not presented. What was the testing time from the sample went into the rig for the first step to the end? This should go into the extended data figure 1, and text.

Thank you for this comment, we have added experimental timings to Extended Data Figure 1, and added the total experimental time to the text, line 358:

"*Here, the total experimental time for an in situ experiment was ~4 hours*."

4.7 - Radiation dose is estimated, which is important. Please present also total dose. If my estimations are correct the total dose would be somewhere around 72 kGy + additional dose from alignment x 4. This is a substantial dose and should be acknowledged and discussed.

We have added further discussion of radiation dose and its potential effects on biomechanics to the discussion:

Line 362: "Radiation dose is also critical, and its impact on IVD mechanics requires further testing. The total radiation dose applied during *in situ* experiments was 70.8 kGy (7.3 kGy per SAXS, 26.6 kGy per pCT), compared to ~2000 kGy that would be required to perform SAXS-TT measurements using the same set-up. This dose level may have led to a stiffening of collagen fibrils(Ng et al., 2025), but is unlikely to have had a major impact on organ or tissue level mechanics, based on results from previous studies at similar dose levels(Dejea et al., 2024; Disney et al., 2026; Parmenter et al., 2025). Total dose for *TomoSAXS* of human IVD was 18.3 kGy (6.6 kGy SAXS, 11.7 kGy HiP-CT)."

4.8 - Line 334 states that about the future prospects of the technique with focus on soft biological tissues. Considering the mechanical challenges of soft tissues (viscoelasticity), long duration of the experiments and the high radiation dose, the reliability of the fibril strain may be questionable. It would be nice to hear the authors thoughts about the future potential.

Thank you for this comment. We agree that soft biological tissues do pose challenges for in situ *TomoSAXS* due to viscoelasticity and radiation dose. In terms of dose, significant reductions could be achieved by using other available beamlines that operate with higher beam energies (e.g. TOMCAT at the Swiss Light Source synchrotron, BM18 at the ESRF – used for human data), and using iterative reconstruction methods for CT.

In terms of viscoelasticity, *TomoSAXS* cannot capture the dynamic response of soft biological materials to load, and there will likely always be a need for a combination of 1D time resolved and 3D quasi-dynamic experiments to fully understand the mechanical behaviour of these complex mechanical systems. Static *TomoSAXS* scans of soft biological tissues offer huge potential to uncover gradients in nanoscale properties in intact organs, without the need for any mechanical testing.

Whilst the focus of our study is soft biological tissues, *TomoSAXS* is applicable to many other fibrous materials that will not have the same experimental limitations, and we have added this to lines 373-376:

"*Looking ahead, the TomoSAXS methodology is broadly applicable to other collagenous and soft biological materials, as well as engineered materials. Indeed, experiments on man-made composites would likely have far fewer limitations in terms of viscoelastic effects, hydration, and radiation dose*."

4.9 - How sensitive is TomoSAXS reconstruction to number of SAXS projections, and beamsize? How large can the discrepancy between resolution of CT and SAXS be, without losing the ability to reconstruct the 3D fibrillar data?

This partly depends on what feature we are trying to reconstruct. The first-order answer is that if we are dealing with concentrated reflections in reciprocal space (characteristic of highly crystalline systems), many angles are needed, as done by other similar methods like SAXS-TT and TexTomo (Frewein et al., 2024) . In contrast, when we have weakly crystalline materials with nanoscale size, like small diameter collagen fibres in cartilage, the equatorial width of the meridional scattering is inversely correlated to the diameter (Badar et al., 2025). So, smaller diameter fibres have a wider equatorial width in reciprocal space and will be intersected even with relatively fewer number of rotations (see also our earlier response R1.6 to reviewer 1-2 on angular resolution), potentially fitted to the 3D diffraction model. Assuming a random sampling of orientations in space, a rough estimate of the angular width of the scattering would be $(k/D_{eq})/(2\pi/D)$ ~10° where k/D_{eq} is the equatorial width of the meridional scattering and D_{eq}, D are the fibril diameter and D-period respectively. The minimum angular step size should be below this to capture every fibre.

Minor comments

4.10 - Aim: The introduction and motivation of the work is presented clearly and compellingly. However a clear aim/hypothesis/research question is missing.
Thank you for this comment, we have added a clear aim to lines 20-21:

"*Our aim is to develop a technique to measure full-field molecular-to-macroscale biophysics of fibrous tissues, and uncover new insights into IVD biomechanics*."

4.11 - Many of the figures are presented with colors and font-sizes that makes it impossible to view and read the details.

Thank you for this comment. We have been through all figures and improved readability.

4.12 - Extended data figure 5 (and also part of 6) is unreadable and it is also unclear from the figure legend what is shown. What lengthscale? What studies? What is FIVD 1-3?

We have edited these Figures, making the font readable, making it less complex (reducing the number of samples shown, removing comparison with previous studies). FIVD1, 2 and 3 are the three IVDs which were analysed in the study. We believe the new Figures are clearer and thank the Reviewer for their comments.

4.13 - For extended data figure 6 b-c, did you try any sample with a simpler fibril orientation in 2D, thus where you would expect your TomoSAXS to result in similar pattern as the 2D direct SAXS mapping?

This is a good point. We address this (and related) in the related question by R1-2 (**R1.17**) and refer the reviewer to that point for our extended response and new Figure. We also suggest that our inclusion of our validation experiment on chicken foot tendon with simpler fibre arrangements also conforms to this suggestion.

4.14 - The application of digital phantoms is a very nice approach to simulate noise in the data and show accuracy of added noise.

Thank you.

4.15 - One possible validation experiment could be to compare with SASTT in a downsampled situation. If you consider the same volumes, how do the techniques correlate then?

This is a very interesting idea and is related to previous R1-2 comments on the SAXS-TT vs *TomoSAXS* question (**R1.17**). We believe the revised Extended Data comparison figure we have created, and related discussion, addresses this question at the single voxel level and we refer the Reviewer to R1.17 for details.

4.16 - In the experiments, how similar were the IVDs in behavior? Why was the experiment done in displacement control instead of strain control?

There were large discrepancies in overall IVD behaviour due to differences in spinal level and size, however, the nano- and microscale relationships reported here held across all 3 samples. An ideal experiment would have had greater similarity between samples, however, there were many potential points of failure in the experimental set-up and only 3 samples successfully made it through the entire pipeline.

Strain control is a good suggestion and could have been used. However, it would have added an extra level of complexity to the overall experiment, and we were originally hoping for greater similarity between the samples than was achieved.

4.17 - The overview of the TomoSAXS algorithm is hard to follow. Could a schematic of what goes into the different modules help? Also here, the total time for computation of the data is unclear? 6-7 days + 4-5 days plus ??

Yes, this is an excellent idea. We have added a schematic Figure S7 with both an overall flowchart and a detailed sketch of the loop wise SAXS solution process in Module 4. Please also see our response to R1.6 on the solving process.

Changes made in text:

New Supplementary Figure S7

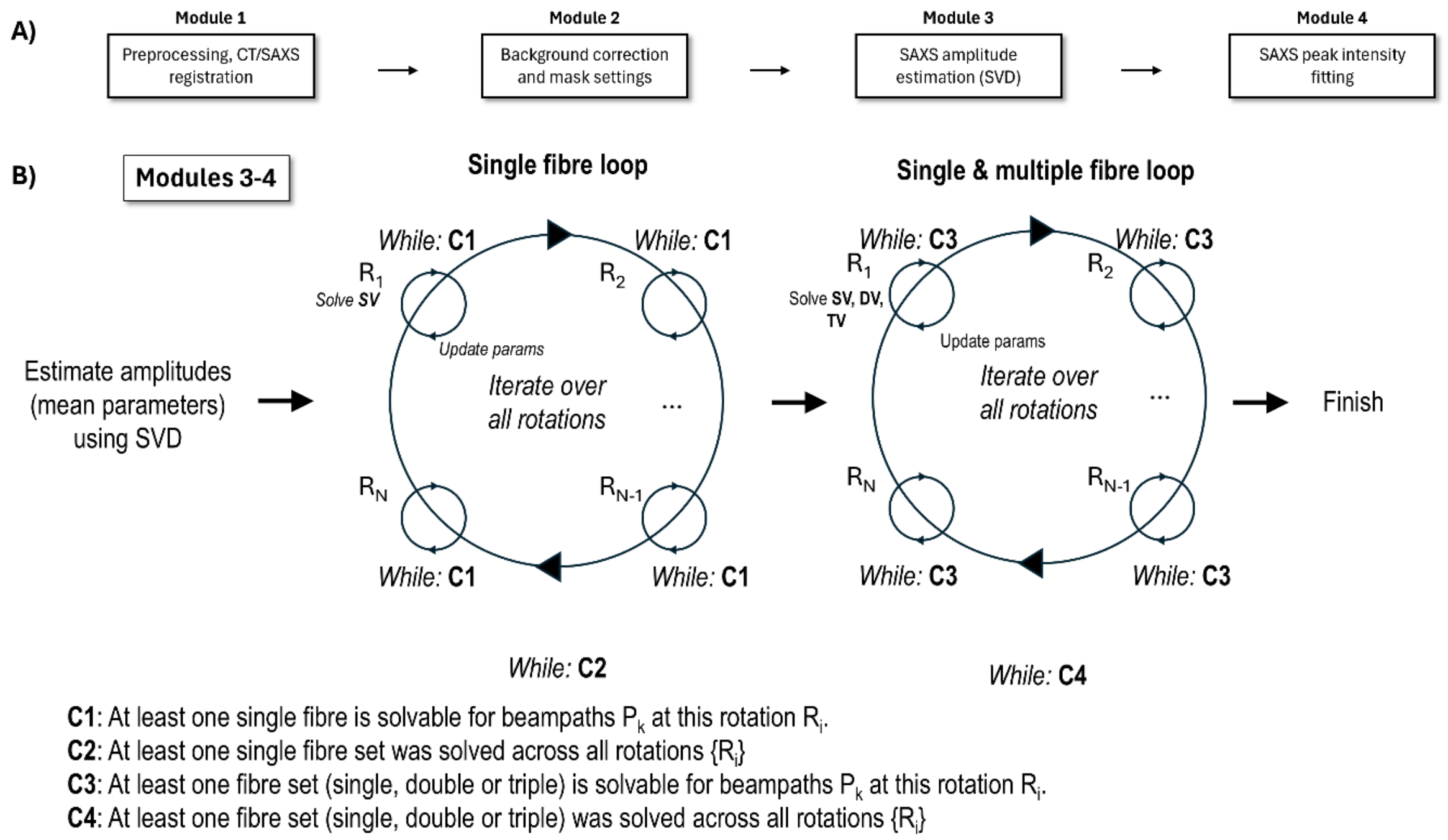

SI Figure 7: *Schematic flowchart: A) overview of the full TomoSAXS pipeline and B) detailed representation of the SAXS reconstruction loop over all beampaths and rotations (1 loop = 1 epoch). First, a single-fibre solution loop is carried out – over all beampaths and rotations – to identify single fibres which can be isolated from the remaining unsolved fibres in the scattering pattern, followed by updating the parameter lists. This loop repeats till no more single fibres can be solved. Then, a second loop iterates over all beampaths and rotations, attempting to solve for both single and multiple (double, triple) isolatable groups of fibres. The loop repeats till no more single or multiple fibres can be solved, i.e. in the last epoch before the reconstruction terminates, no fibre or group of fibres were solved. See also* ***Fig S3 C***.

4.18 - Statistical analysis: This section is not possible to assess. It is unclear what is compared and it reads does not read as a methods section. Why were the methods selected? What was compared? Please motivate the approach and explain what is presented.

While the original format followed *Nature* publishing conventions in letter, the Reviewer is right that due to the volume of information it was very hard to assess in practice.

We have now converted the statistical analysis into a more readable Table form:

Correlations and Statistical Analysis

Ordinary Least Squares (OLS) regression analysis was used to test the agreement of known versus estimated properties of digital phantom data with and without the addition of

experimental noise (Table 1). Correlations between per-fibre mean values of fibre strain, D-period, D-period variation, and D-period strain were tested using OLS regression (Table 2).

Table 1 – Ordinary least squares regression analysis of known digital phantom parameters versus estimated parameters from *TomoSAXS* with and without the addition of experimental noise.

Variable x	Variable y	Linear fit	r^2	p-value
**Known D-period (nm)**	Estimated D-period (nm) *(with noise)*	$y = 0.646x + 0.010$	0.813	0.0001
	Estimated D-period (nm) *(noise-free)*	$y = 0.780x + 15.033$	0.874	0.0001
**Known D-period variation (nm)**	Estimated D-period variation (nm) *(with noise)*	$y = 0.826x + 0.001$	0.846	0.0001
	Estimated D-period variation (nm) *(noise-free)*	$y = 0.851x + 0.392$	0.883	0.0001
**Known fibril diameter (nm)**	Estimated fibril diameter (nm) *(with noise)*	$y = 0.704x + 0.007$	0.797	0.0001
	Estimated fibril diameter (nm) *(noise-free)*	$y = 0.924x + 3.635$	0.915	0.0001

Table 2 - Summary of OLS regression analysis performed on per-fibre mean values.

Variable x	Variable y	Linear fit	r^2	p-value

**Fibre strain (%)**	Curvature (mm^{-1})	$y = 0.035x + 0.001$	0.480	0.0001
**Fibre strain (%)**	D-period strain (%)	$y = 0.347x + 0.024$	0.138	0.0001
**Initial D-period (nm)**	D-period strain (%)	$y = -1.357x + 0.040$	0.312	0.0001
**Initial D-period (nm)**	D-period variation (nm)	$y = 0.107x + 0.010$	0.089	0.0001
**D-period strain (%)**	D-period variation (nm)	$y = -0.035x + 0.005$	0.067	0.0001

Supplementary information
4.19 - Supp table 1 – first time mentioning number of samples. Unclear if they are all from the same rat or three different rats? Why did you not use the same IVD/spinal segments to also gain some idea of reproducibility? Also why did the strain vary so much?

Each of our three samples originate from 3 different rats, which is now made clear in Table S1 (thank you for bringing this to our attention). Sample preparation and alignment were conducted during our beamtime (as flash freezing prior to experimentation was not performed to ensure sample integrity), which proved to be extremely difficult and resulted in more samples prepared than were eventually usable (due to alignment of IVD versus vertebral endplates, sample movement between scanning). Samples were chosen based on which ones were well aligned with the beam. Ideally we would have used the same spinal level for all samples, but this proved impossible during the experiment for the above reasons. Strain thus varied due to the differences in IVD size and shape (reflecting their different positions along the spine), and the same displacement was applied to each sample.

4.20 - Supp note 9: It is important to assess zero strain noise. However, in this experiment you also move the sample across DLS during the experiment. Did you do any zero strain analysis including moving the sample in and out? It would give a better understanding of the uncertainties involved.

That is a good point. Unfortunately, we didn't do this during our experiment. We were very time limited, running an experiment across two beamlines, with timelines longer due to RF cavity issue at the accelerator (as mentioned in **R1.5**). We have performed analyses of the SAXS and WAXD intensity map and radiograph map for coarse assessment of whether the sample has moved. (This was used to determine suitability of samples after moving between beamlines.). This has been addressed in **R3.13**.

4.21 - Supp note 19: Includes statistics that has not been introduced in the statistics earlier. No motivation of type of data and type of statistical testing chosen.

We thank the reviewer for highlighting this. Kruskal-Wallis testing was used in this section to compare distributions between estimated values using 2D SAXS and *TomoSAXS*, as their distributions were significantly different and so precluded parametric comparisons. We have now added this as a note to this section of the supplement:

"*Kruskal-Wallis testing was used throughout this analysis to compare distributions of estimates from 2D SAXS and TomoSAXS, as their distributions were consistently different and non-normal (Extended Data Figure 6) which precluded the use of parametric statistical comparisons."*

References:

Andriotis, O. G., Nalbach, M., & Thurner, P. J. (2023). Mechanics of isolated individual collagen fibrils. *Acta Biomaterialia*, *163*(1), 35–49. https://doi.org/10.1016/j.actbio.2022.12.008

Badar, W., Ali, H., Brooker, O. N., Newham, E., Snow, T., Terrill, N. J., Tozzi, G., Fratzl, P., Knight, M. M., & Gupta, H. S. (2022). Collagen pre-strain discontinuity at the bone—Cartilage interface. *PLOS ONE*, *17*(9), e0273832. https://doi.org/10.1371/journal.pone.0273832

Badar, W., Inamdar, S. R., Fratzl, P., Snow, T., Terrill, N. J., Knight, M. M., & Gupta, H. S. (2025). Nonlinear Stress-Induced Transformations in Collagen Fibrillar Organization, Disorder and Strain Mechanisms in the Bone-Cartilage Unit. *Advanced Science*, *12*(1), 2407649. https://doi.org/10.1002/advs.202407649

Crameri, F., Shephard, G. E., & Heron, P. J. (2020). The misuse of colour in science communication. *Nature Communications 2020 11:1*, *11*(1), 5444-. https://doi.org/10.1038/s41467-020-19160-7

Crowther, R. A., Derosier, D. J., & Klug, A. (1970). RECONSTRUCTION OF A THREE-DIMENSIONAL STRUCTURE FROM PROJECTION AND ITS APPLICATION TO ELECTRON MICROSCOPY. *Proc. Roy. Soc. Loud. A*, *317*(1530), 319–340. https://doi.org/10.1098/RSPA.1970.0119

Dejea, H., Pierantoni, M., Orozco, G. A., B. Wrammerfors, E. T., Gstöhl, S. J., Schlepütz, C. M., & Isaksson, H. (2024). In Situ Loading and Time-Resolved Synchrotron-Based Phase Contrast Tomography for the Mechanical Investigation of Connective Knee Tissues: A Proof-of-Concept Study. *Advanced Science*, *11*(21), 2308811. https://doi.org/10.1002/advs.202308811

Disney, C. M., Eckersley, A., McConnell, J. C., Geng, H., Bodey, A. J., Hoyland, J. A., Lee, P. D., Sherratt, M. J., & Bay, B. K. (2019). Synchrotron tomography of intervertebral

disc deformation quantified by digital volume correlation reveals microstructural influence on strain patterns. *Acta Biomaterialia*, *92*, 290–304. https://doi.org/10.1016/j.actbio.2019.05.021

Disney, C. M., Lees, R. M., Parker, A. W., Atwood, R. C., & Burca, G. (2026). Preserving tissue integrity under the beam: high-energy, low-dose synchrotron CT for in situ imaging of bovine intervertebral discs. *Acta Biomaterialia*, *213*(1), 496–507. https://doi.org/10.1016/j.actbio.2026.02.033

Disney, C. M., Mo, J., Eckersley, A., Bodey, A. J., Hoyland, J. A., Sherratt, M. J., Pitsillides, A. A., Lee, P. D., & Bay, B. K. (2022). Regional variations in discrete collagen fibre mechanics within intact intervertebral disc resolved using synchrotron computed tomography and digital volume correlation. *Acta Biomaterialia*, *138*, 361–374. https://doi.org/10.1016/j.actbio.2021.10.012

Dusfour, G., Ambard, D., Cañadas, P., & Lefloch, S. (2021). Residual strains estimation in the annulus fibrosus through digital image correlation. *Journal of Theoretical, Computational and Applied Mechanics*, 1–15. https://doi.org/10.46298/jtcam.6971

Eppell, S. J., Smith, B. N., Kahn, H., & Ballarini, R. (2006). Nano measurements with micro-devices: mechanical properties of hydrated collagen fibrils. *Journal of The Royal Society Interface*, *3*(6), 117–121. https://doi.org/10.1098/rsif.2005.0100

Fratzl, P., Fratzl-Zelman, N., & Klaushofer, K. (1993). Collagen packing and mineralization. An x-ray scattering investigation of turkey leg tendon. *Biophysical Journal*, *64*(1), 260–266. https://doi.org/10.1016/S0006-3495(93)81362-6

Frewein, M. P. K., Mason, J., Maier, B., Cölfen, H., Medjahed, A., Burghammer, M., Allain, M., & Grünewald, T. A. (2024). Texture tomography, a versatile framework to study crystalline texture in 3D. *IUCrJ*, *11*(5), 809–820. https://doi.org/10.1107/S2052252524006547

Lyu, F.-J., Cui, H., Pan, H., MC Cheung, K., Cao, X., Iatridis, J. C., & Zheng, Z. (2021). Painful intervertebral disc degeneration and inflammation: from laboratory evidence to clinical interventions. *Bone Research*, *9*(1), 7. https://doi.org/10.1038/s41413-020-00125-x

Masic, A., Bertinetti, L., Schuetz, R., Chang, S.-W., Metzger, T. H., Buehler, M. J., & Fratzl, P. (2015). Osmotic pressure induced tensile forces in tendon collagen. *Nature Communications*, *6*(1), 5942. https://doi.org/10.1038/ncomms6942

Ng, K., Allam, N., Neshatian, M., Vaez, M., Hirvonen, L. M., Lam, E., Vitkin, A., & Bozec, L. (2025). Effects of Ionizing Radiation on the Biophysical Properties of Type I Collagen Fibrils. *PLOS ONE*, *20*(4), e0319777. https://doi.org/10.1371/journal.pone.0319777

Parmenter, A. L., Newham, E., Sharma, A., Disney, C. M., Deyhle, H., Bosi, F., Terrill, N. J., Bay, B. K., Pitsillides, A. A., Gupta, H. S., & Lee, P. D. (2025). Variations in mineral prestrain, nanostructure, and microarchitecture play a role in intervertebral disc loading. *Cell Biomaterials*, *0*(0), 100151. https://doi.org/10.1016/J.CELBIO.2025.100151

Pierantoni, M., Silva Barreto, I., Hammerman, M., Novak, V., Diaz, A., Engqvist, J., Eliasson, P., & Isaksson, H. (2023). Multimodal and multiscale characterization reveals how tendon structure and mechanical response are altered by reduced loading. *Acta Biomaterialia*, *168*, 264–276. https://doi.org/10.1016/J.ACTBIO.2023.07.021

Rosenberg, J. L., Schaible, E., Bostrom, A., Lazar, A. A., Graham, J. L., Stanhope, K. L., Ritchie, R. O., Alliston, T. N., Lotz, J. C., Havel, P. J., Acevedo, C., & Fields, A. J. (2023). Type 2 diabetes impairs annulus fibrosus fiber deformation and rotation under disc compression in the University of California Davis type 2 diabetes mellitus (UCD-T2DM) rat model. *PNAS Nexus*, *2*(12), 1–8. https://doi.org/10.1093/pnasnexus/pgad363

Schollum, M. L., Wade, K. R., Robertson, P. A., Thambyah, A., & Broom, N. D. (2018). A Microstructural Investigation of Disc Disruption Induced by Low Frequency Cyclic Loading. *Spine*, *43*(3), E132–E142. https://doi.org/10.1097/BRS.0000000000002278

Yang, B., & O'Connell, G. D. (2019). Intervertebral disc swelling maintains strain homeostasis throughout the annulus fibrosus: A finite element analysis of healthy and degenerated discs. *Acta Biomaterialia*, *100*(4), 61–74. https://doi.org/10.1016/j.actbio.2019.09.035

Referees' comments:

We thank all reviewers for their positive and constructive comments. We have addressed the specific points raised and made changes to the manuscript as request; see details below.

Referee #1 (Remarks to the Author):

The revised version of this manuscript by Newham et al., reporting on a multi-modal X-ray imaging approach combining phase contrast tomography and SAXS convincingly includes the novel method, including all necessary details and validation, as well as the results on animal and human intervertebral disc.
The comments made on the first draft have all been carefully and exhaustively answered. The balance to make this manuscript accessible to the broad audience of nature while including all necessary details has been worked on very well. New illustrations and schematics are greatly aiding the understandability. The manuscript is still very dense, and at parts not easy to digest, but this should not be taken as a critique, but is rather a result from merging novel method development with interesting findings on biological application, which of course does increase the significance and potential impact of this publication. I would like to congratulate the authors for an impressive piece of work.
While there was already significant work shown for the validation of the method in the fist version, the authors have now extended even further by introducing a tendon which due to unidirectional orientation provides an easier test case. The authors have also added discussion towards the very low dose needed compared to other approaches, which highlights even more the significance in particular towards in-situ measurements of mechanical test on radiation sensitive samples. Limitations of the method are appropriately discussed and conclusions drawn are carefully considering those.

one very minor detail to cross-check: in the rebuttal letter 1.6 II. It is stated in the answer that the solved fibers are in the range of 25-60%, in the changes made to the manuscript it is stated that 35-85% of all detectable fibers were solved, check which number is the correct one to be added to the manuscript.

We thank the reviewers for this comment – the overall percentage of traced fibres solved in the rat IVD case is 25-60 %. A significant portion of unsolved fibres were

not detectable due to sparse angular sampling meaning that ~30 % of fibres never entered the diffraction condition. Of the fibres that did enter diffraction condition, 35-85 % of fibres were solved. We have made this distinction between detected and solved fibres in the discussion as the number of detectable fibres can be improved through experimental design, whereas the number of solved fibres can be improved though computational analysis methods.

We have edited the manuscript accordingly:

*"Here, the relatively large angular step (22.5°) used for rotations in the rat IVD case (**Methods**) limited the number of fibres detectable under Ewald conditions to ~70 %, which can be improved in future by higher angular resolution (5°-10°); Supplementary Fig. 5a). In each horizontal slice around 35-85% of the detectable fibres were solved, which were distributed reasonably uniformly (but not densely) across the tissue volume (Supplementary Fig. 6). To improve spatial resolution, future work may look to improve the SAXS reconstruction pipeline by adding higher-order overlaps to increase the fraction of solvable fibres or adapting alternate global solve strategies."*

Referee #2 (Remarks to the Author):

I co-reviewed this manuscript with one of the reviewers who provided the listed reports.

Referee #3 (Remarks to the Author):

I believe the authors have addressed most of my comments satisfactorily. There are three, however, listed below that I believe require a further response:

1. Line 237 – "This can be interpreted as fibres with an initially tensile pre-strain undergoing compression upon loading, and those with an initial compressive pre-strain undergoing tension". My understanding is that fibres can only resist tension so this wording is strange to me. Is it possible for fibres to withstand compression? Do they not just buckle? Please could you expand your discussion here to explain to

other readers who may have the same assumption about fibres only resisting tension?

We thank the reviewer for this comment, and can see how this result contradicts fundamental assumptions made when modelling fibre mechanics. Fibres are generally assumed to have negligible cross-sectional width, and will therefore buckle under any compressive loading. Here, this assumption breaks down, as we are measuring strains across a length of ~67 nm in fibrils which have diameters ranging from 20 – 120 nm. Therefore, at this length scale, collagen fibrils can no longer be assumed to mechanically behave as idealised fibres, and should instead be thought of as columns.

Additionally, potential buckling of fibres is moderated by the matrix when the overall tissue is under compressive pre-strain. We have added the following to the main manuscript:

"This can be interpreted as IVD tissues with fibres under initial tensile pre-strain undergoing compression upon loading, and those with an initial compressive pre-strain undergoing tension. Although it seems counterintuitive, at the nanoscale, where strain is measured over lengths of ~67 nm in fibrils with diameters 20-120 nm, collagen fibrils can support compressive loading, with the surrounding extrafibrillar matrix providing lateral support that suppresses buckling."

2. Line 243 – "…residual stress in the IVD may result in fibrillar strains close to failure" – Do you mean fibrillar stresses close to failure in this sentence? And do you think it makes sense that residual stresses are so high? I note that this has been reported in other tissues, but it seems strange to me that the stresses are so close to failure at quite a low load. Why would this be beneficial from a physiological perspective? I would have expected these stresses to be substantially below the failure stresses, so I believe the manuscript should include additional discussion on this.

We thank the reviewer for this comment, and agree that the physiological implications of these high residual strains warrant further discussion. Although it may initially seem surprising that fibrillar residual strains approach damaging levels, we interpret this as reflecting a fundamental mechanical trade-off. The load/deformation

response of these hybrid gel/fibre materials is highly nonlinear, with effective elastic modulus changing throughout. One mechanical role of pre-strain is to allow tuning of the stiffness characteristics of the tissue in response to functional requirements. Another role is to provide capacity for the tissue to deform.

In the neutral, unstrained tissue configuration, material stiffness is low. Pre-stress, particularly in tension, moves the tissue into a higher stiffness configuration, which can be mechanically beneficial. The trade-off, however, is lower capacity for additional tension before damaging strain levels are reached. Compressive pre-strain provides large capacity for tensile deformation when tissue stiffness is less functionally significant.

It is beneficial to have high fibrillar pre-strains as this is a mechanism to increase overall IVD tissue stiffness to mechanically useful levels. Fibrillar density and arrangement, as well as IVD lamellar structure, also influence tissue stiffness, but to a limited extent without the additional effects of fibrillar strain. Unfortunately, and as demonstrated by high levels of degradation and failure, this also places the tissue closer to functional limits. The tissue is required to satisfy multiple competing demands and is constrained by the fundamental capabilities of the constituent materials. Thus, the seeming contradiction of a high-function material configuration close to failure.

We have added the following to the results:

"residual stress in the IVD may result in fibrillar strains close to failure. This may help explain why the annulus fibrosus is susceptible to microdamage at mechanical loads well below those required to induce failure in the disc[38]. Similar high pre-stresses have been reported before for tendon collagen and mineralised collagen fibrils[39,40], and may be required to moderate material stiffness to meet the challenging mechanical requirements of the IVD."

And discussion:

"High fibrillar residual stresses position the IVD in a mechanically advantageous state, but leave the IVD vulnerable to fibrillar-level damage. This correlates with clinically seen high levels of degeneration in the IVD[13-16]. Pre-strain stiffens an otherwise compliant tissue, enabling physiological load-bearing while reducing the

remaining margin before damaging strains are reached. This illustrates a fundamental trade-off in fibre-reinforced biological tissues, whereby mechanical performance is achieved at the expense of increased susceptibility to microdamage and degeneration."

3. Comment 3.12 from previous round – "20 minutes stress relaxation" – What was your definition of "stabilise" (in manuscript text) or "plateau" (in your rebuttal). I.e. less that X% change in X minutes.

We thank the reviewer for this comment. We have added the following statement to the methods:

"Samples were allowed 20 minutes stress relaxation before scanning to minimise motion, determined as the time taken for load readings to stabilise (less than 0.01 N change in 1 minute)."

Referee #4 (Remarks to the Author):

I would like to thank the authors for the extensive response to all reviewers comments and for clearly addressing all queries. The manuscript and work represents an excellent contribution to the scientific community!

There are still a number of figures in extended data where the font sizes are not readable, which would benefit from minor editing.

We thank the reviewer for this comment, and have increased the text size in extended data figures.

Referee #4 (Remarks on code availability):

I have downloaded and browsed the code, but I have not fully understood it or spend enough time to state that I have reviewed it.
